# Using Machine Learning to Improve Control for Confounding in the Dynamic Weighted Ordinary Least Squares Estimator of Optimal Adaptive Treatment Strategies

**DOI:** 10.1002/bimj.70068

**Published:** 2025-07-29

**Authors:** Kossi Clément Trenou, Miceline Mésidor, Aida Eslami, Hermann Nabi, Caroline Diorio, Denis Talbot

**Affiliations:** ^1^ Département de médecine sociale et préventive Université Laval Québec Canada; ^2^ Axe santé des populations et pratiques optimales en santé Centre de recherche du CHU de Québec – Université Laval Québec Canada; ^3^ Centre Armand‐Frappier Santé Biotechnologie Institut national de la recherche scientifique Québec Canada; ^4^ Institut Universitaire de Cardiologie et de Pneumologie de Québec – Université Laval Québec Canada; ^5^ Axe oncologie Centre de recherche du CHU de Québec – Université Laval Québec Canada

**Keywords:** adaptive treatment strategies, cross‐fitting, dynamic weighted ordinary least squares (dWOLS), machine learning, personalized medicine

## Abstract

Estimating optimal adaptive treatment strategies (ATSs) can be done in several ways, including dynamic weighted ordinary least squares (dWOLS). This approach is doubly robust as it requires modeling both the treatment and the response, but only one of those models needs to be correctly specified to obtain a consistent estimator. For estimating an average treatment effect, doubly robust methods have been shown to combine better with machine learning methods than alternatives. However, the use of machine learning within dWOLS has not yet been investigated. Using simulation studies, we evaluate and compare the performance of the dWOLS estimator when the treatment probability is estimated either using machine learning algorithms or a logistic regression model. We further investigate the use of an adaptive m‐out‐of‐n bootstrap method for producing inferences. SuperLearner performed at least as well as logistic regression in terms of bias and variance in scenarios with simple data‐generating models and often had improved performance in more complex scenarios. Moreover, the m‐out‐of‐n bootstrap produced confidence intervals with nominal coverage probabilities for parameters that were estimated with low bias. We also apply our proposed approach to the data from a breast cancer registry in Québec, Canada, to estimate an optimal ATS to personalize the use of hormonal therapy in breast cancer patients. Our method is implemented in the R software and available on GitHub https://github.com/kosstre20/MachineLearningToControlConfoundingPersonalizedMedicine.git. We recommend routine use of machine learning to model treatment within dWOLS, at least as a sensitivity analysis for the point estimates.

## 1 Introduction

Personalized medicine is a growing field in which individual information is used to recommend personal treatment. Adaptive treatment strategies (ATSs) involve tailoring an individual's treatment plan according to their characteristics. An optimal ATS is one that optimizes a clinical outcome, thereby making it suitable for personalized medicine.

Estimation of an optimal ATS is often achieved by estimating how the effect of the treatment varies according to the characteristics of the patients, that is, by estimating conditional treatment effects. When observational data are used to estimate such treatment effects, it is essential to adjust for confounding factors, since treated and untreated patients can differ according to characteristics that are predictive of the outcome. Various statistical methods can be used to estimate an optimal ATS with observational data, including reinforcement learning (Q‐learning; Watkins 1989) and dynamic weighted ordinary least squares (dWOLS; Wallace and Moodie 2015). Because Q‐learning is based on linear regression models of the outcome, it relies on stringent parametric assumptions in terms of correct model specification to adequately control confounding bias. While dWOLS shares many implementation similarities with Q‐learning, it benefits from a double robustness property. Indeed, while dWOLS requires specifying a model for both the outcome and the treatment, it is consistent if either of those models is correctly specified. As such, dWOLS requires less stringent modeling assumptions than Q‐learning to adequately control confounding. Recursive G‐estimation is another, arguably more complex, doubly robust approach for estimating optimal ATSs (Robins 2004).

When the goal is to estimate an average treatment effect, rather than an optimal ATS, doubly robust methods have been demonstrated to combine better with machine learning than alternative approaches, since statistical properties such as $\sqrt{n}$‐consistency and the ability to produce valid statistical inferences can be preserved (Balzer and Westling 2023; Naimi et al. 2023). The purpose of using machine learning methods is to limit the risk of incorrect specification of the models since the modeling is flexible and data‐adaptive, thus limiting measured confounding bias. However, few studies have used machine learning methods to control confounding in optimal ATS estimation. A notable exception is Robust Q‐learning (RQL, Ertefaie et al. 2021), an extension of Q‐learning developed to allow the use of machine learning to control confounding bias. Similar to dWOLS, RQL models both the treatment and the outcome, but it is not a doubly robust approach since it requires the model for the treatment to be consistent with a convergence rate $O(n^{-1/4})$.

The aim of this paper is to investigate, through simulation studies, whether the use of machine learning methods to model the treatment in dWOLS reduces the bias due to model misspecification in comparison to a parametric model. This approach offers a solution to enhance the robustness of dWOLS, providing a method that is conceptually simple and easy to implement, making it particularly appealing to analysts. The contribution lies in the thorough simulation experiments that illustrate the practical benefits of this method, especially in complex settings with limited sample sizes. In Section 2, we introduce some notation and briefly review the dWOLS estimator of an optimal ATS. In Section 3, we detail the approach we propose for incorporating machine learning within dWOLS. Then, in Section 4, we perform simulation studies exploring different scenarios to evaluate the performance of our proposed approach in comparison with RQL. In Section 5, we present the results of the simulation studies. In Section 6, we provide an application of the methods on real data. Finally, in Section 7, we discuss the results and perspectives for future research.

## 2 Notation and Estimation of an Optimal ATS With dWOLS

### 2.1 Notation

We consider a longitudinal study with t=1,…,T time points where the objective is to determine the optimal decision rule at each time point. Let $X_{t}$ represent the patients' characteristics at time t, $A_{t}$ the treatment received at time t, $d_{t}$ the treatment strategy at time t, and Y the final outcome to optimize. Without loss of generality, we assume that greater values of Y reflect a better outcome, noting that Y can be recoded to achieve this otherwise. We further use overbars to denote the history of a covariate up to a given time point, for example, ${\bar{A}}_{t}=\left(A_{1},\text{…},A_{t}\right)$, and underbars to denote the future values of a covariate, for example, ${\underline{A}}_{t}=\left(A_{t},\text{…},A_{T}\right)$. Using this notation, we further denote by $H_{t}=\left({\bar{A}}_{t-1},{\bar{X}}_{t}\right)$ the patient information (or history) up to time t.

To define the causal parameter of interest, we use the counterfactual framework to causal inference, wherein, for example, $Y^{{\bar{d}}_{T}}$ denotes the outcome that would have been observed under the treatment strategy ${\bar{d}}_{T}$. The optimal treatment strategy is recursively defined, from time point t=T,…,1 as

$$\begin{array}{cc} d_{t}^{opt}\left(h_{t}\right) & =\underset{a_{t}}{\text{argmax}}\;E\left[Y^{{\bar{a}}_{t-1},a_{t},{\underline{d}}_{t+1}^{opt}}-Y^{{\bar{a}}_{t-1},0,{\underline{d}}_{t+1}^{opt}}|H_{t}=h_{t}\right]\\ & \equiv \underset{a_{t}}{\text{argmax}}\;\gamma_{t}\left(a_{t},h_{t}\right). \end{array}$$

The quantity $\gamma_{t}\left(a_{t},h_{t}\right)$ is often called the blip and represents the effect of treatment $A_{t}=a_{t}$ versus $A_{t}=0$ among people with history $H_{t}=h_{t}$ assuming that future treatment decisions are optimal (i.e., assuming that ${\underline{A}}_{t+1}={\underline{d}}_{t+1}^{opt}$). The nonparametric identification of this causal effect can be achieved under the following assumptions:

i. *No interference*, signifies that the treatment of an individual i does not affect the response of other subjects j. $\left\{Y_{i}^{a_{i},a_{j}}=Y_{i}^{a_{i},a_{j}^{\prime }}=Y_{i}^{a_{i}}\right\}$.
ii. *Consistency*, implies the ability to observe the counterfactual outcome corresponding to the truly assigned treatment. $\left\{If{\bar{A}}_{t}={\bar{a}}_{t}\;\text{then}\;Y^{{\bar{a}}_{t}}=Y\right\}$.
iii. *Positivity*, which requires that everyone has a strictly positive probability of receiving all levels of the treatment of interest. $\left\{P\left[A_{t}|H_{t}=h_{t}\right]>0,t=1,\text{…},T\right\}$.
iv. *Sequential conditional exchangeability*, which requires that the treatment effect at each time point is not confounded by unobserved factors. $\left\{A_{t}∐\left(Y^{{\bar{a}}_{T}},X_{t+1}^{{\bar{a}}_{t}},\text{…},X_{T}^{{\bar{a}}_{T-1}}\right)|H_{t},t=1,\text{…},T\right\}$.

**FIGURE 1:**
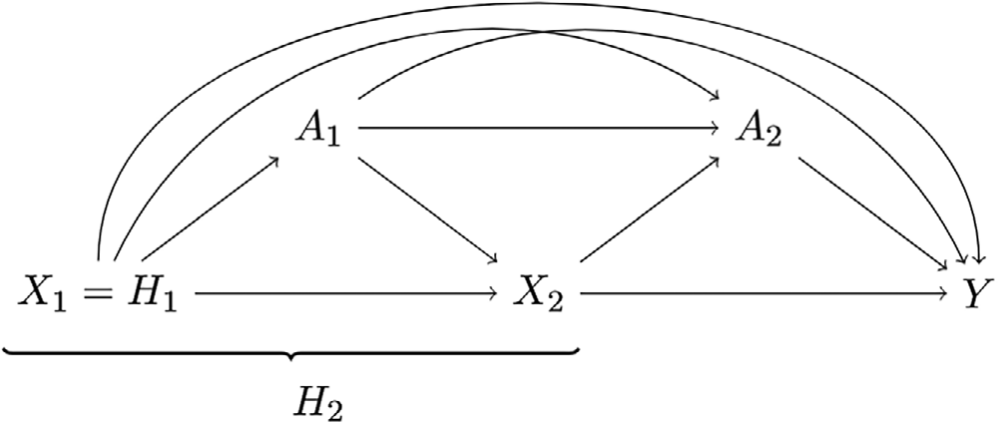
Directed acyclic graph representing the data structure with two time points. *Note*. $X_{t}$: patients' characteristics at time t; $A_{t}$: treatment received at time t; $H_{t}$: patient information (or history) up to time t; and Y: final outcome and time t=1,2.

Figure 1 presents a directed acyclic graph (DAG) that summarizes the notation and for which the sequential conditional exchangeability assumption would hold.

### 2.2 Estimation of an Optimal ATS Using dWOLS

Because the dWOLS estimator shares several implementation similarities with the Q‐learning estimator, we first present a brief overview of Q‐learning before reviewing dWOLS. The Q‐learning method is based on reinforcement learning and can be used to solve optimization problems (Watkins 1989). Q‐learning makes use of Q‐functions for estimating the optimal ATS

$$\begin{array}{cc} Q_{T}\left(h_{T},a_{T}\right) & =E[Y|H_{T}=h_{t},A_{T}=a_{t}]\;\text{and}\\ Q_{t}\left(h_{t},a_{t}\right) & =E[\underset{A_{t+1}}{\max\;}Q_{t+1}\left(H_{t+1},A_{t+1}\right)|H_{t}=h_{t},A_{t}=a_{t}],\\ & \;\;\;\;\text{for}\;t=1,\text{…},T-1,\\ d_{t}^{opt}\left(h_{t}\right) & =\underset{a_{t}}{\text{argmax}\;}Q_{t}\left(a_{t},h_{t}\right),\;\;t=1,\text{…},T. \end{array}$$

Linear models of the form $Q_{t}\left(H_{t},A_{t},\beta_{t},\psi_{t}\right)=\beta_{t}^{⊤}H_{t}+\psi_{t}^{⊤}H_{t}A_{t}$ are often used to model these Q‐functions, where the parameters are estimated using ordinary least squares. Under such models, the estimated optimal treatment strategy is $d_{t}^{opt}\left(h_{t}\right)=0$ if ${\hat{\psi }}_{t}^{⊤}h_{t}<0$, $d_{t}^{opt}\left(h_{t}\right)=1$ if ${\hat{\psi }}_{t}^{⊤}h_{t}>0$, and undefined if ${\hat{\psi }}_{t}^{⊤}h_{t}=0$. A more detailed presentation of Q‐learning can be found elsewhere (Chakraborty and Moodie 2013; Murphy 2003; Wallace and Moodie 2014, 2015; Watkins 1989).

The crucial difference between dWOLS and Q‐learning lies in how they estimate model parameters; dWOLS uses a weighted least squares estimator instead of an ordinary (unweighted) least squares estimator. To ensure double robustness of the estimator, the weights must balance treatment groups at each time point. More formally, denoting by $w(a_{t},h_{t})$ the weight at time point t attributed to a subject with covariates $\left(a_{t},h_{t}\right)$, a consistent estimator is obtained if $w\left(1,h_{t}\right)P\left(A_{t}=1|h_{t}\right)=w\left(0,h_{t}\right)P\left(A_{t}=0|h_{t}\right)$ for all $h_{t}$ (Wallace and Moodie 2015). These weights are generally constructed according to the predicted values of a parametric expectation model $E[A_{t}|H_{t}]$, such as a logistic regression in the case of a binary treatment. Various weights specification satisfy the balancing property, but the weights $w_{t}=\left|a_{t}-E\left[A_{t}|H_{t}\right]\right|$ have been observed to increase efficiency in the binary treatment case (Wallace and Moodie 2015).

## 3 Integration of Machine Learning Within the dWOLS Estimator

An intuitive approach for improving robustness of dWOLS without affecting interpretability is to use machine learning techniques to model $E[A_{t}|H_{t}]$. When estimating an average treatment effect, the use of machine learning methods for modeling the treatment probability has had variable success in simulation studies. While multiple simulation studies have observed that machine learning methods can help control confounding bias due to parametric treatment model misspecifications (Kreif et al. 2016; Wang et al. 2023), others have suggested that machine learning methods may fail to reduce bias as compared to incorrect parametric models and may even increase bias under some scenarios (Alam et al. 2019; Diop et al. 2022; Naimi et al. 2023). One potential reason for this is that machine learning methods can be prone to overfitting and thus accentuate practical positivity violations (Diop et al. 2022). To avoid this issue, we propose employing a cross‐fitting algorithm when modeling the treatment probability of dWOLS with machine learning methods.

Various cross‐fitting algorithms have been proposed (Chernozhukov et al. 2018; Zivich and Breskin 2021), but the general principle is to first randomly split the data into disjoint subsets of approximately equal size. A model for the nuisance function (here $E[A_{t}|H_{t}]$) is then fitted on one of the subset and predictions are made on the other. Next, the roles of the subsets are reversed. In the end, predictions are made for each observation using a model fitted on different observations. These predictions are then plugged into the estimator of the causal effect of interest. Because estimation and prediction are done on separate splits, the procedure avoids overfitting. In addition, because each split serves, in turn, both for estimation and for prediction, the procedure makes an efficient use of the data. The use of twofold cross‐fitting has been shown to strike a good balance between computational efficiency and performance, especially in high‐dimensional settings with limited sample sizes (Ellul et al. 2024). Moreover, studies have indicated that increasing the number of folds beyond two does not substantially improve point estimates and may only add to the computational burden without offering significant gains in accuracy (Ellul et al. 2024).

The specific cross‐fitting algorithm we propose is similar to that employed in RQL (Ertefaie et al. 2021). For t=T,…,1:

i. Randomly split the data in two disjoint subsets of approximately equal size.
ii. Use machine learning algorithms to model $E[A_{t}|H_{t}]$ in each split separately.
iii. For each split, compute the predicted treatment probabilities for all observations using the output of the model that was fitted in the other split.
iv. Using these predicted treatment probabilities, compute the weights $w_{t}$ for all observations.
v. Estimate $\psi_{t}$ using a weighted least squares estimator of the linear regression model $Q_{t}\left(H_{t},A_{t},\beta_{t},\psi_{t}\right)=\beta_{t}^{⊤}H_{t}+\psi_{t}^{⊤}H_{t}A_{t}$ for all observations together.

The other steps, notably the estimation of the optimal treatment strategy $d_{T}^{opt}\left(h_{T}\right)$ and of the pseudo‐outcomes $Q_{t}$, are done the same as described in Section 2.2.

### 3.1 Inferences

Producing inferences for the dWOLS estimator when estimating the treatment probability with machine learning is challenging. The usual nonparametric bootstrap is not expected to be consistent in this situation. Indeed, the estimator may behave differently in the bootstrap samples than in does in random samples from the data generating distribution (Coyle and van der Laan 2018). This is because some observations may be sampled more than once in the bootstrap resampling, and different replications of the same observation may be allocated to different splits in the cross‐fitting procedure, effectively creating dependence between the splits. The m‐out‐of‐n bootstrap, where m<n observations are resampled with m appropriately chosen, is consistent under milder conditions (Politis and Romano 1994; Bickel et al. 1997; Bickel and Sakov 2008). For the m‐out‐of‐n bootstrap with replacement, the key requirements are:

1. m→∞ and m/n→0 to ensure weak convergence.
2. The limiting distribution of the bootstrap statistic matches that of the true statistic for appropriately chosen m.
3. The statistic is not too affected by the number of ties.

Various methods are available for choosing m in a data‐adaptive way (see Dalitz and Lögler 2024, for a review). In the simulation study, we used the following algorithm proposed by Bickel and Sakov (2008):

1. Estimate the parameters of the blip in the full sample. Denote the estimate $\hat{\psi }$.
2. For j=0,…,K
  a. Let $m_{j}=\left\lfloor q^{j}\cdot n\right\rfloor$.
  b. For b=1,…,B
    i. Take a sample of size $m_{j}$ with replacement among the n observations.
    ii. Estimate the parameters of the blip ψ using this sample. Denote the estimate ${\hat{\psi }}_{jb}$
  (c) For each blip parameter, calculate the empirical cumulative density function of $\sqrt{m_{j}}\left({\hat{\psi }}_{jb}-\hat{\psi }\right)$. Denote it $R_{j}\left(x\right)$.
3. For each parameter, choose $j=\underset{j}{\text{argmax}}\;\underset{x}{\text{sup}}\left|R_{j}\left(x\right)-R_{j+1}\left(x\right)\right|$.

The idea of this algorithm is that the distribution of the bootstrap statistic should remain relatively stable in a neighborhood around the appropriate value of m. Based on the observations of Chakraborty et al. (2013), we chose q=0.95 and set K=14 so that the minimum value of m≈n/2.

To construct a confidence interval, the convergence rate of the estimator needs to be known or estimated. Under the assumption that the convergence rate is of the form $n^{\beta }$, we estimated the convergence rate by running a linear regression of $\log(\hat{Var}\left({\hat{\psi }}_{j}\right))$ according to $-2\log(m_{j})$ using the bootstrap samples (Bertail et al. 1999). We finally computed a 95% confidence intervals by calculating $\hat{\psi }\pm 1.96{\left(\frac{m}{n}\right)}^{\beta }\sqrt{\hat{Var}\left({\hat{\psi }}_{j}\right)}$ for the selected j (and corresponding sample size $m_{j}$). This approach assumes that the estimator is asymptotically normally distributed.

## 4 Simulation Studies

The primary aim of the simulation studies was to empirically assess the adequacy of modeling the treatment probability in dWOLS using machine learning methods and cross‐fitting for estimating the parameters of a blip. The secondary aims were to compare the performance of different machine learning methods in this procedure and to compare our proposed approach to parametric dWOLS and to RQL. To this end, five simulation studies were conducted. The first three studies are delineated as follows: (1) only the outcome, (2) only the treatment, and (3) neither the outcome nor the treatment were generated using a simple parametric model with a single covariate $X_{t}$ at each time point. The fourth study extended Study 3 by incorporating a lack of positivity. Building on Study 4, Study 5 introduced increased dimensionality with 10 covariates. Because dWOLS is a double robust estimator, unbiased estimates were expected regardless if machine learning was employed or not to model the treatment probability in Studies 1 and 2. These studies thus served to verify that machine learning would not introduce bias or increase the variance. However, in Studies 3–5, parametric dWOLS is expected to be biased, and our proposed approach was hypothesized to reduce this bias. Studies 4 and 5 were designed to investigate the performance of the methods in particularly challenging settings. Simulations with two time points were conducted.

### 4.1 Scenarios

The five simulation studies we considered were adapted from previously published work (Talbot et al. 2023; Wallace and Moodie 2015). Three scenarios were considered in each study. The simple scenarios are designed using linear terms for the treatment and/or the outcome. The medium scenarios include quadratic terms, while the complex scenarios incorporate, in addition to these terms, absolute value and trigonometric terms.

In Study 1, the outcome variable was generated according to a simple parametric equation. As for the treatment variable, three scenarios with varying degree of complexity were designed. For Study 2, the treatment variable was generated based on a simple parametric equation. As for the outcome variable, three scenarios were designed to vary the complexity of the outcome generation equations. In Studies 3–5, both the treatment's and the outcome's generating equations were of varying complexity. See Appendices 1 and 2 in the Online Supporting Information for the detailed generating equations as well as figures depicting the distribution of the treatment probabilities for Studies 1–4 and Study 5, respectively. These figures notably reveal a lack of overlap in the covariates distribution across treatment groups in Study 4, and to a lesser extent in Study 5.

When analyzing the simulated data, $A_{2}$ was modeled as a function of $X_{1}$, $X_{2}$, and $A_{1}$. $A_{1}$ was modeled as a function of $X_{1}$. Modeling was performed using the learning algorithms described in the next section. For dWOLS, the right‐hand side of the outcome model at the second time point included main terms for $X_{1}$, $X_{2}$, $A_{1}$, and $A_{2}$, as well as two‐way interactions between $A_{1}$ and $X_{1}$, $A_{1}$ and $X_{2}$, and $A_{2}$ and $X_{2}$. At the first time point, the right‐hand side included main terms for $A_{1}$, $X_{1}$ and interaction terms between these. For RQL, the same variables as described above were included when modeling the outcome at the second and first time points, respectively, but modeling was achieved using the SuperLearner method described below.

A total of 1000 replicates were performed for each scenario of each study. The sample sizes of n=300 and n=1000 were used to investigate how the performance varied according to sample size. Due to the important computational burden, the m‐out‐of‐n bootstrap was only investigated with the SuperLearner in the complex scenario of Study 4 with a sample size of n=300 and 200 replications. We have also investigated in Study 4 how the performance of dWOLS with SuperLeaner varied according to the number of folds in the cross‐fitting with the following implementations: no cross‐fitting, two‐, three‐, four‐, and fivefold cross‐fitting.

### 4.2 Algorithms

We considered some of the most popular machine learning algorithms in recent years (Balzer and Westling 2023; Naimi et al. 2023; Xiao and Sun 2021; Rose and Rizopoulos 2020). We also restricted our attention to algorithms packaged in the R software version 4.3.0 (Hastie et al. 2009; James et al. 2013). A total of six algorithms were identified, most of which were used with their default parameters.

i. Standard logistic regression (Logit). We have used the *glm* function.
ii. Random forest (RF, Breiman 2001) were implemented using the randomForest package (Liaw and Wiener 2002) with the default parameters.
iii Naive bayes model (Bayes, Webb et al. 2010) as implemented in the package e1071 (Meyer et al. 2020).
iv. Support vector machines (SVMs, Cortes and Vapnik 1995) based on the *SVM* function in the e1071 package (Meyer et al. 2020).
v. Neural networks (Neural, Ripley 2007) as implemented in the *nnet* function of the nnet package (Venables and Ripley 2021). We used three nodes in the hidden layer and set the weight decay parameter to 0.1 to control regularization.
vi. Ensemble SuperLearner (SL, van der Laan et al. 2007) as implemented in the SuperLearner package. We included the following algorithms in the SL: SL.glm (McCullagh and Nelder 1989), SL.randomForest (Breiman 2001), SL.nnet (Ripley 2007), SL.glm.interaction, and SL.svm (Cortes and Vapnik 1995) except in Studies 4 and 5. The default parameters were used.

Furthermore, SVM could not be considered in Study 4 because of software errors that occurred in this specific scenario.

### 4.3 Performance Metrics

We have evaluated the performance of the different methods of confounding adjustment using various measures (Morris et al. 2019). The estimated bias: {Bias = mean estimate-true value}; the standard deviation of the estimated blip coefficients $\left\{\sqrt{\text{variance of estimate}}\right\}$ and the root‐mean‐square error: {RMSE =$\sqrt{{\left(\text{mean estimate - true value}\right)}^{2}+\text{variance of estimate}}$}. To facilitate interpretation, we present the ratio between the RMSE of each different methods versus logistic regression (Ratio). When evaluating the m‐out‐of‐n bootstrap, we calculated the proportion of the simulation replications where the 95% confidence interval included the true value of the parameter. Furthermore, we report the maximum Monte Carlo error in each scenario: {Monte Carlo error of the Bias = $\text{standard deviation of estimate}\;/\sqrt{\text{Number of repetitions}}\}$; {Monte Carlo error of the standard deviation = $\text{standard deviation of estimate}\;/\sqrt{2(\text{Number of repetitions}-1)}\}$ and the Monte Carlo error of the coverage {Monte Carlo error of the coverage = $\sqrt{\text{coverage}(1-\text{coverage})/\text{Number of repetitions}}$}.

## 5 Results

We report the simulation results for a sample size of 300 for Studies 1–4, with results for a sample size of 1000 presented in Appendix 1 in the Online Supporting Information. Because of space constraints, the results for Study 5 for both sample sizes are provided in Appendix 2 in the Online Supporting Information, and those of the investigation of the number of folds are presented in Appendix 3. The results were similar between the sample sizes of 300 and 1000. The Monte Carlo errors were sufficiently small not to affect the interpretation of the results (see Appendices 1 and 2 in the Online Supporting Information for Studies 1–4 and Study 5, respectively).

The results of Study 1 are summarized in Table 1. Recall that the outcome is generated according to a simple parametric model and the complexity of the treatment generating model varies in this study. Because of the double robustness of dWOLS, it was expected that all methods would have low bias since the outcome is modeled according to a correctly specified parametric model. As expected, all methods for modeling the treatment had relatively low biases across scenarios, except for SVM that had a larger bias for estimating $\psi_{11}^{*}$ in the medium scenario (−0.20) and, to a lesser extent, in the complex scenario (0.11). The standard deviation of the estimates is also similar for most methods, except for RF and SVM that have noticeably increased standard deviations for at least some parameters in all scenarios. The ratio of RMSE compared to logistic regression illustrates that no method overall performed better than the logistic regression. However, Neural and SL performed as well as logistic regression. Due to a combination of slightly increased bias and slightly increased standard deviations, Bayes and RQL had a somewhat larger RMSE than the logistic regression for at least some parameters.

**TABLE 1:** Results of Study 1: Simple outcome model and varying complexity of treatment model, n = 300.

	Simple scenario				Medium scenario				Complex scenario			
Methods	First time		Second time		First time		Second time		First time		Second time	
	$\psi_{10}^{*}$	$\psi_{11}^{*}$	$\psi_{20}^{*}$	$\psi_{21}^{*}$	$\psi_{10}^{*}$	$\psi_{11}^{*}$	$\psi_{20}^{*}$	$\psi_{21}^{*}$	$\psi_{10}^{*}$	$\psi_{11}^{*}$	$\psi_{20}^{*}$	$\psi_{21}^{*}$
	Bias				Bias				Bias			
Logit	0.00	0.09	−0.01	0.01	−0.05	0.07	−0.08	0.00	0.05	−0.07	0.08	−0.01
RF	0.00	0.03	−0.03	0.04	−0.01	0.00	−0.02	0.01	0.00	−0.01	0.01	0.00
Bayes	0.01	−0.05	0.03	−0.06	−0.01	−0.02	−0.01	−0.02	0.02	0.01	−0.01	0.04
Neural	0.00	0.07	−0.03	0.06	−0.04	0.02	−0.06	0.01	0.03	−0.02	0.06	−0.02
SVM	0.02	−0.04	0.01	−0.03	−0.03	−0.20	−0.02	−0.09	0.03	0.11	0.08	0.00
SL	0.00	0.04	−0.02	0.03	−0.03	0.02	−0.05	0.02	0.02	−0.01	0.04	−0.02
RQL	0.00	−0.02	0.00	−0.01	−0.02	0.01	−0.02	0.01	−0.01	−0.03	0.04	−0.05

	Standard deviation				Standard deviation				Standard deviation			
Logit	0.17	0.30	0.16	0.17	0.17	0.26	0.15	0.15	0.16	0.29	0.15	0.14
RF	0.19	0.35	0.17	0.18	0.19	0.33	0.17	0.16	0.18	0.35	0.16	0.15
Bayes	0.17	0.32	0.16	0.18	0.16	0.28	0.16	0.16	0.16	0.29	0.15	0.15
Neural	0.17	0.30	0.16	0.16	0.16	0.26	0.15	0.15	0.16	0.28	0.15	0.14
SVM	0.18	0.36	0.18	0.20	0.18	0.39	0.19	0.21	0.17	0.35	0.18	0.17
SL	0.17	0.30	0.16	0.17	0.16	0.27	0.16	0.15	0.16	0.28	0.15	0.14
RQL	0.18	0.32	0.18	0.18	0.16	0.29	0.16	0.16	0.16	0.29	0.17	0.15

	RMSE				RMSE				RMSE			
Logit	0.17	0.31	0.16	0.17	0.17	0.27	0.17	0.15	0.17	0.30	0.17	0.14
RF	0.19	0.35	0.17	0.18	0.19	0.33	0.17	0.17	0.18	0.35	0.16	0.15
Bayes	0.17	0.32	0.17	0.19	0.16	0.28	0.16	0.16	0.16	0.29	0.15	0.15
Neural	0.17	0.31	0.16	0.17	0.16	0.27	0.16	0.15	0.16	0.28	0.16	0.14
SVM	0.18	0.37	0.18	0.20	0.18	0.44	0.19	0.23	0.17	0.37	0.19	0.17
SL	0.17	0.31	0.16	0.17	0.17	0.27	0.16	0.16	0.16	0.28	0.16	0.14
RQL	0.18	0.32	0.18	0.18	0.16	0.29	0.16	0.16	0.16	0.29	0.17	0.16

	Ratio				Ratio				Ratio			
RF	1.15	1.12	1.07	1.08	1.09	1.20	0.98	1.11	1.09	1.17	0.97	1.10
Bayes	0.98	1.03	1.04	1.14	0.93	1.02	0.91	1.06	0.95	0.97	0.90	1.11
Neural	0.97	0.99	1.01	1.05	0.95	0.97	0.94	1.01	0.95	0.95	0.93	1.01
SVM	1.06	1.18	1.11	1.21	1.05	1.59	1.09	1.53	1.04	1.25	1.14	1.25
SL	0.98	0.98	1.00	1.02	0.95	0.99	0.95	1.05	0.94	0.96	0.92	1.03
RQL	1.04	1.02	1.14	1.11	0.94	1.07	0.95	1.10	0.95	0.98	1.01	1.15

In Study 2, the treatment was generated according to a simple parametric model and the complexity of the outcome varied. Remember that it was again expected that no method for modeling the treatment would perform better than a logistic regression in this scenario. In this study, most machine learning methods had somewhat increased bias as compared to the logistic regression for at least one parameter in at least one scenario (see Table 2). Neural and Bayes were the methods for which the increase in bias was the largest, whereas RQL and SVM had the lowest bias. As in Study 1, RF and SVM sometimes had increased standard deviations as compared to other methods. Overall, SL and RQL had the performance that was most similar to a logistic regression, with RMSE ratios between 0.72 and 1.11, and 0.70 and 1.14, respectively.

**TABLE 2:** Results of Study 2: Simple treatment model and varying complexity of outcome model, n = 300.

	Simple scenario				Medium scenario				Complex scenario			
Methods	First time		Second time		First time		Second time		First time		Second time	
	$\psi_{10}^{*}$	$\psi_{11}^{*}$	$\psi_{20}^{*}$	$\psi_{21}^{*}$	$\psi_{10}^{*}$	$\psi_{11}^{*}$	$\psi_{20}^{*}$	$\psi_{21}^{*}$	$\psi_{10}^{*}$	$\psi_{11}^{*}$	$\psi_{20}^{*}$	$\psi_{21}^{*}$
	Bias				Bias				Bias			
Logit	0.00	0.09	−0.01	0.01	0.01	−0.52	0.02	−0.04	−0.01	0.34	−0.01	0.02
RF	0.00	0.03	−0.03	0.04	0.01	−0.13	0.08	−0.15	0.00	0.06	−0.06	0.10
Bayes	0.01	−0.05	0.03	−0.06	−0.03	0.13	−0.09	0.15	0.02	−0.19	0.12	−0.20
Neural	0.00	0.07	−0.03	0.06	0.00	−0.30	0.10	−0.19	0.00	0.20	−0.07	0.15
SVM	0.02	−0.04	0.01	−0.03	−0.07	0.06	0.00	−0.02	0.05	−0.10	0.04	−0.04
SL	0.00	0.04	−0.02	0.03	0.00	−0.16	0.06	−0.10	−0.01	0.09	−0.04	0.07
RQL	0.00	−0.02	0.00	−0.01	−0.02	−0.06	0.01	−0.02	0.00	−0.01	0.00	0.00

	Standard deviation				Standard deviation				Standard deviation			
Logit	0.17	0.30	0.16	0.17	0.25	0.47	0.20	0.22	0.19	0.34	0.19	0.20
RF	0.19	0.35	0.17	0.18	0.28	0.53	0.20	0.22	0.21	0.39	0.19	0.21
Bayes	0.17	0.32	0.16	0.18	0.24	0.50	0.20	0.24	0.19	0.36	0.19	0.22
Neural	0.17	0.30	0.16	0.16	0.23	0.46	0.20	0.23	0.18	0.34	0.19	0.21
SVM	0.18	0.36	0.18	0.20	0.27	0.74	0.25	0.33	0.20	0.53	0.24	0.30
SL	0.17	0.30	0.16	0.17	0.23	0.47	0.20	0.23	0.18	0.34	0.18	0.20
RQL	0.18	0.32	0.18	0.18	0.25	0.48	0.19	0.19	0.19	0.35	0.18	0.19

	RMSE				RMSE				RMSE			
Logit	0.17	0.31	0.16	0.17	0.25	0.70	0.20	0.23	0.19	0.48	0.19	0.20
RF	0.19	0.35	0.17	0.18	0.28	0.55	0.21	0.26	0.21	0.40	0.20	0.23
Bayes	0.17	0.32	0.17	0.19	0.24	0.52	0.22	0.28	0.19	0.41	0.22	0.30
Neural	0.17	0.31	0.16	0.17	0.23	0.55	0.23	0.30	0.18	0.40	0.20	0.26
SVM	0.18	0.37	0.18	0.20	0.28	0.75	0.25	0.33	0.21	0.54	0.24	0.30
SL	0.17	0.31	0.16	0.17	0.23	0.50	0.21	0.25	0.18	0.35	0.19	0.22
RQL	0.18	0.32	0.18	0.18	0.25	0.48	0.19	0.20	0.19	0.35	0.18	0.19

	Ratio				Ratio				Ratio			
RF	1.15	1.12	1.07	1.08	1.13	0.79	1.05	1.17	1.09	0.82	1.08	1.13
Bayes	0.98	1.03	1.04	1.14	0.98	0.74	1.09	1.25	0.98	0.85	1.18	1.45
Neural	0.97	0.99	1.01	1.05	0.95	0.78	1.10	1.33	0.94	0.83	1.05	1.27
SVM	1.06	1.18	1.11	1.21	1.14	1.07	1.23	1.46	1.08	1.12	1.28	1.48
SL	0.98	0.98	1.00	1.02	0.95	0.72	1.02	1.11	0.94	0.74	0.99	1.07
RQL	1.04	1.02	1.14	1.11	1.01	0.70	0.91	0.86	0.96	0.72	0.95	0.93

In Study 3, both the treatment and the outcomes were generated according to equations of varying complexities and employing machine learning was expected to reduce bias as compared to logistic regression. As shown in Table 3, RF and RQL had almost no bias for all parameters. Opposingly, SVM had very large biases for $\psi_{11}^{*}$ in the medium and complex scenarios (0.97 and 0.43, respectively). SVM and RF again had importantly increased standard deviations as compared to other methods. When looking at the RMSE ratio, Neural, SL and RQL all achieved similar or appreciably better performance than the logistic regression.

**TABLE 3:** Results of Study 3: Varying complexity of treatment and outcome models, n = 300.

	Simple scenario				Medium scenario				Complex scenario			
Methods	First time		Second time		First time		Second time		First time		Second time	
	$\psi_{10}^{*}$	$\psi_{11}^{*}$	$\psi_{20}^{*}$	$\psi_{21}^{*}$	$\psi_{10}^{*}$	$\psi_{11}^{*}$	$\psi_{20}^{*}$	$\psi_{21}^{*}$	$\psi_{10}^{*}$	$\psi_{11}^{*}$	$\psi_{20}^{*}$	$\psi_{21}^{*}$
	Bias				Bias				Bias			
Logit	0.00	0.02	−0.01	0.01	0.19	−0.08	0.19	0.04	0.14	−0.01	0.22	−0.03
RF	0.00	0.03	−0.03	0.04	0.02	−0.04	0.04	−0.03	0.01	−0.02	0.03	−0.02
Bayes	0.01	−0.05	0.03	−0.06	0.06	0.02	0.00	0.08	0.07	0.04	−0.04	0.13
Neural	0.00	0.07	−0.03	0.06	0.16	−0.18	0.14	0.01	0.11	−0.11	0.16	−0.06
SVM	0.02	−0.04	0.01	−0.03	0.06	0.97	0.02	0.27	0.08	0.43	0.21	−0.01
SL	0.00	0.04	−0.02	0.03	0.12	−0.13	0.12	−0.03	0.08	−0.06	0.11	−0.05
RQL	0.00	−0.02	0.00	−0.01	0.02	−0.06	0.04	−0.02	0.02	−0.02	0.05	−0.04

	Standard deviation				Standard deviation				Standard deviation			
Logit	0.16	0.31	0.16	0.17	0.24	0.45	0.17	0.19	0.17	0.32	0.17	0.15
RF	0.19	0.35	0.17	0.18	0.27	0.54	0.18	0.20	0.20	0.38	0.17	0.17
Bayes	0.17	0.32	0.16	0.18	0.24	0.44	0.18	0.21	0.17	0.31	0.17	0.18
Neural	0.17	0.30	0.16	0.16	0.24	0.44	0.17	0.20	0.17	0.31	0.16	0.16
SVM	0.18	0.36	0.18	0.20	0.28	0.73	0.26	0.34	0.19	0.46	0.24	0.25
SL	0.17	0.30	0.16	0.17	0.24	0.45	0.17	0.20	0.17	0.31	0.16	0.16
RQL	0.18	0.32	0.18	0.18	0.25	0.47	0.17	0.18	0.17	0.30	0.16	0.15

	RMSE				RMSE				RMSE			
Logit	0.16	0.31	0.16	0.17	0.31	0.46	0.26	0.19	0.22	0.32	0.28	0.16
RF	0.19	0.35	0.17	0.18	0.27	0.54	0.19	0.20	0.20	0.38	0.18	0.17
Bayes	0.17	0.32	0.17	0.19	0.25	0.45	0.18	0.22	0.19	0.32	0.18	0.22
Neural	0.17	0.31	0.16	0.17	0.28	0.48	0.22	0.20	0.20	0.33	0.23	0.17
SVM	0.18	0.37	0.18	0.20	0.28	1.21	0.26	0.43	0.20	0.63	0.32	0.25
SL	0.17	0.31	0.16	0.17	0.27	0.47	0.21	0.20	0.19	0.32	0.19	0.17
RQL	0.18	0.32	0.18	0.18	0.25	0.47	0.18	0.18	0.17	0.30	0.17	0.15

	Ratio				Ratio				Ratio			
RF	1.18	1.14	1.07	1.08	0.89	1.18	0.73	1.03	0.89	1.20	0.64	1.06
Bayes	1.01	1.05	1.04	1.14	0.80	0.98	0.69	1.16	0.83	0.99	0.64	1.39
Neural	1.00	1.01	1.01	1.05	0.93	1.05	0.88	1.03	0.91	1.04	0.83	1.08
SVM	1.09	1.20	1.11	1.21	0.93	2.66	1.01	2.27	0.91	1.98	1.16	1.60
SL	1.01	1.00	1.00	1.02	0.87	1.03	0.83	1.05	0.84	1.00	0.70	1.07
RQL	1.07	1.03	1.14	1.11	0.81	1.04	0.69	0.94	0.77	0.95	0.61	0.96

Study 4 is similar to Study 3 but features lack of positivity issues. RF and RQL exhibited minimal biases across all parameters and scenarios (Table 4). Conversely, the Neural model displayed greater biases for $\psi_{10}^{*}$ in the medium scenario and for $\psi_{20}^{*}$ in the complex scenario (1.04 and 0.76, respectively). RQL and RF had substantially greater standard deviations compared to other methods. In terms of RMSE ratios, most methods performed similarly to or markedly better than logistic regression in every scenario.

**TABLE 4:** Results of Study 4: Varying complexity of treatment and outcome models, and practical positivity violations, n = 300.

	Simple scenario				Medium scenario				Complex scenario			
Methods	First time		Second time		First time		Second time		First time		Second time	
	$\psi_{10}^{*}$	$\psi_{11}^{*}$	$\psi_{20}^{*}$	$\psi_{21}^{*}$	$\psi_{10}^{*}$	$\psi_{11}^{*}$	$\psi_{20}^{*}$	$\psi_{21}^{*}$	$\psi_{10}^{*}$	$\psi_{11}^{*}$	$\psi_{20}^{*}$	$\psi_{21}^{*}$
	Bias				Bias				Bias			
Logit	0.00	0.03	−0.01	0.03	1.37	−0.23	1.62	0.29	0.86	0.03	1.21	−0.19
RF	0.00	0.07	−0.06	0.11	0.09	0.16	0.16	0.09	0.03	0.09	0.06	0.03
Bayes	0.00	−0.02	0.06	−0.09	0.25	−0.01	0.03	0.05	0.21	−0.10	−0.13	0.11
Neural	0.00	0.18	−0.08	0.18	1.06	−0.52	0.79	0.20	0.67	−0.41	0.76	−0.27
SL	0.00	0.05	−0.02	0.06	0.70	0.02	0.45	0.24	0.45	0.01	0.26	−0.02
RQL	−0.01	0.02	−0.01	0.01	0.11	−0.12	0.12	0.03	0.01	0.21	0.10	−0.07

	Standard deviation				Standard deviation				Standard deviation			
Logit	0.20	0.25	0.17	0.15	0.51	0.82	0.38	0.36	0.31	0.44	0.33	0.22
RF	0.24	0.29	0.17	0.15	0.54	0.97	0.30	0.33	0.33	0.49	0.27	0.23
Bayes	0.21	0.26	0.17	0.16	0.45	0.82	0.26	0.31	0.27	0.43	0.28	0.25
Neural	0.20	0.23	0.17	0.14	0.46	0.81	0.31	0.34	0.28	0.47	0.27	0.24
SL	0.20	0.25	0.16	0.15	0.46	0.85	0.30	0.35	0.28	0.44	0.27	0.25
RQL	0.21	0.27	0.18	0.16	0.46	1.20	0.22	0.24	0.27	0.39	0.19	0.14

	RMSE				RMSE				RMSE			
Logit	0.20	0.25	0.17	0.15	1.46	0.85	1.66	0.46	0.91	0.44	1.25	0.29
RF	0.24	0.30	0.18	0.19	0.54	0.98	0.34	0.34	0.33	0.50	0.28	0.24
Bayes	0.21	0.26	0.18	0.19	0.51	0.82	0.26	0.32	0.34	0.44	0.31	0.27
Neural	0.20	0.29	0.18	0.23	1.16	0.97	0.85	0.39	0.72	0.62	0.81	0.36
SL	0.20	0.25	0.17	0.16	0.84	0.85	0.54	0.43	0.53	0.44	0.37	0.25
RQL	0.21	0.28	0.18	0.16	0.47	1.21	0.25	0.24	0.27	0.44	0.21	0.15

	Ratio				Ratio				Ratio			
RF	1.16	1.20	1.10	1.25	0.37	1.15	0.20	0.74	0.36	1.13	0.22	0.81
Bayes	1.02	1.03	1.10	1.24	0.35	0.97	0.16	0.68	0.37	1.00	0.25	0.91
Neural	1.00	1.16	1.11	1.55	0.79	1.13	0.51	0.86	0.79	1.40	0.64	1.24
SL	1.00	1.01	1.00	1.05	0.57	0.99	0.33	0.93	0.58	1.00	0.30	0.84
RQL	1.04	1.09	1.08	1.07	0.32	1.42	0.15	0.53	0.29	1.00	0.17	0.52

In Study 5 with more covariates, RF, Neural, and SL showed relatively low biases, with SL performing particularly well, as evidenced by the low bias across most parameters. SVM also exhibited relatively low bias, except for a few parameters where it showed higher bias, similar to what was observed in the previous studies. Most methods showed similar standard deviations, except for RF and SVM, which presented increased standard deviations in some cases, consistent with Studies 1–4. In terms of RMSE, SL consistently outperformed the other methods, showing results comparable to logistic regression and, in some cases, surpassing it. RF, Bayes, and Neural showed similar performance, while SVM and RQL had higher RMSE due to increased variability in some scenarios.

The coverage of the 95% confidence intervals constructed with the m‐out‐of‐n bootstrap procedure in the complex scenario of Study 4 was 61.5%, 93.0%, 81.5%, and 91.5% for $\psi_{10}$, $\psi_{11}$, $\psi_{20}$, and $\psi_{21}$, respectively. Since the bias for these parameters were 0.44, 0.06, 0.26, and −0.02, respectively, undercoverage was expected for $\psi_{10}$ and $\psi_{20}$. The mean value of m selected was around 218, 226, 220, and 220 for $\psi_{10}$, $\psi_{11}$, $\psi_{20}$, and $\psi_{21}$, respectively. The mean convergence rate was estimated to be approximately $n^{0.54}$ for all parameters. In the simulation investigating the number of splits in the cross‐fitting, either not doing cross‐fitting at all or using twofold cross‐fitting always had the best performance in terms of bias, standard deviations, and RMSE.

## 6 Application

### 6.1 Context

Hormonal therapy is currently recommended to breast cancer patients whose cancer is hormone receptor (HR)‐positive, that is, if their cancer cells express HRs. However, not all HR‐positive cancers respond to hormonal therapy as expected. Because hormonal therapy can have important side effects (Canadian Cancer Society 2024), it is crucial that hormonal therapy be targeted to patients who will truly benefit from it. In a previous study (Talbot et al. 2023), we hypothesized that obesity may be a relevant variable to consider for developing an improved ATS because obesity is associated with poorer outcomes among breast cancer patients (Calle and Kaaks 2004; Chan et al. 2014). Furthermore, considering body mass index (BMI) as a variable to personalize treatment recommendations is advantageous because it can easily be measured in clinical practice without additional costs. Our previous results suggested that hormonal therapy may be slightly more beneficial for women with HR‐positive cancer that have a BMI equal or greater than 25 ${\text{kg/m}}^{2}$ than among those with a HR‐positive cancer and a normal BMI, although confidence intervals were large (Talbot et al. 2023). However, the analyses relied on observational data that could potentially be affected by important confounding biases given the major differences between treatment groups that were observed. These biases may not have been fully controlled for by the parametric models that were used to model the outcome and the treatment. To illustrate the methods that we proposed in this paper, we conducted a reanalysis of these data using machine learning methods to model the treatment probability. Based on our simulation results, we expected this reanalysis to potentially have both reduced bias and variance, and thus provide more valid and precise effect estimates.

### 6.2 Data and Analysis

The analyzed data pertain to women diagnosed with nonmetastatic breast cancer between 1987 and 2009, who are part of the breast cancer registry maintained by the Centre des Maladies du Sein de l'Hôpital du Saint‐Sacrement du CHU de Québec – Université Laval, Québec, Canada. We conducted a comparison of the years of survival since breast cancer diagnosis (Y) between two groups of women: those who received hormonal therapy (A = 1) and those who did not (A = 0). Information on vital status was accessible until December 31, 2011 (administrative end of follow‐up) through linkage with Quebec administrative databases. In the initial data set, data on age, BMI, menopausal status, smoking status, cancer grade and stage, estrogen receptor status, progesterone receptor status, type of surgery, first‐degree family history of breast cancer, hormone replacement therapy, chemotherapy, radiotherapy, trastuzumab, and year of diagnosis were considered as potential confounders. These variables were selected based on domain knowledge by the experts within our team.

Before proceeding with the analysis, a preliminary processing of the database was conducted. Some missing data (see Table 5) were imputed to their most likely values according to contextual and clinical knowledge. When menopausal status was missing, it was imputed as “no” if age was <50 years and “yes” if age was ≥50 years. Missing values regarding chemotherapy and radiotherapy were imputed with “no” for chemotherapy and “yes” for radiotherapy. Missing data on smoking status, progesterone status, and cancer grade were treated as an “unknown” category. Observations with missing data on other variables were removed, resulting in the inclusion of 5444 individuals in the analysis. BMI was categorized as normal if below 25 ${\text{kg/m}}^{2}$ and overweight/obese if 25 ${\text{kg/m}}^{2}$ or higher. HR status was considered positive (HR+) if either or both estrogen and progesterone receptors statuses were positive, negative (HR‐) if both estrogen and progesterone receptors statuses were negative, and unknown (Unknown) if estrogen receptor was negative and progesterone receptor was unknown.

**TABLE 5:** Overview of nonmetastatic breast cancer patients from 1987 to 2010 at the Breast Cancer Registry of Centre des Maladies du Sein, Quebec, Canada, based on hormonal therapy treatment. All numbers are n (%).

	Treatment			
Variable	No hormonal therapy	Hormonal therapy	SMD	% Missing
n	1800 (33.0)	3644 (67.0)		
Age (%)			0.419	0.00
≤39 years	186 (10.3)	119 (3.3)		
40–49 years	496 (27.6)	625 (17.2)		
50–59 years	477 (26.5)	1189 (32.6)		
60–69 years	353 (19.6)	1009 (27.7)		
≥70 years	288 (16.0)	702 (19.3)		
Smoking status (%)			0.039	0.00
Never	992 (55.1)	2011 (55.2)		
Ever	739 (41.1)	1519 (41.7)		
Unknown	69 (3.8)	114 (3.1)		
BMI ≥ 25 ${\text{kg/m}}^{2}$ (%)	743 (41.3)	1773 (48.7)	0.149	0.00
Menopause status = postmenopausal (%)	1142 (63.4)	2769 (76.0)	0.276	13.25
Grade (%)			0.791	0.00
1	225 (12.5)	1055 (29.0)		
2	343 (19.1)	1406 (38.6)		
3	994 (55.2)	838 (23.0)		
Unknown	238 (13.2)	345 (9.5)		
Estrogen ‐ (%)	981 (54.5)	127 (3.5)	1.360	0.00
Progesterone (%)			0.840	0.00
+	604 (33.6)	2627 (72.1)		
−	999 (55.5)	805 (22.1)		
Unknown	197 (10.9)	212 (5.8)		
Hormone receptor status (HR) (%)			1.284	0.00
+	910 (50.6)	3564 (97.8)		
−	822 (45.7)	62 (1.7)		
Unknown	68 (3.8)	18 (0.5)		
Stage (%)			0.077	0.93
I	809 (44.9)	1671 (45.9)		
II	746 (41.4)	1569 (43.1)		
III	245 (13.6)	404 (11.1)		
Surgery (%)			0.076	0.00
Mastectomy	482 (26.8)	867 (23.8)		
Breast‐conserving	1289 (71.6)	2730 (74.9)		
None	29 (1.6)	47 (1.3)		
Family history of breast cancer = yes (%)	399 (22.2)	980 (26.9)	0.110	12.81
Hormone replacement therapy usage = yes (%)	630 (35.0)	1724 (47.3)	0.252	14.52
Chemotherapy = yes (%)	977 (54.3)	1527 (41.9)	0.250	0.28
Radiotherapy = yes (%)	1307 (72.6)	2875 (78.9)	0.147	0.82
Herceptin therapy = yes (%)	70 (3.9)	113 (3.1)	0.043	0.38
Year (%)			0.729	0.00
1985–1989	269 (14.9)	119 (3.3)		
1990–1994	384 (21.3)	328 (9.0)		
1995–1999	402 (22.3)	513 (14.1)		
2000–2004	328 (18.2)	1164 (31.9)		
2005–2009	417 (23.2)	1520 (41.7)		

Abbreviations: BMI: Body Mass Index; SMD = standardized mean difference.

Based on the results of the simulation analysis, we employed both the Logit and the SL estimator to model the treatment. For each estimator, the treatment model was adjusted by including all potential confounding factors and twofold cross‐fitting was employed for the SL. The outcome model included all potential confounding factors, and interaction terms between treatment and both HS and BMI as a composite variable with four levels (HR+ and BMI < 25 ${\text{kg/m}}^{2}$, HR+ and BMI ≥ 25 ${\text{kg/m}}^{2}$, HR− and BMI < 25 ${\text{kg/m}}^{2}$, HR− and BMI ≥ 25 ${\text{kg/m}}^{2}$). As in Talbot et al. (2023), to address censoring, we used a parametric accelerated failure time model as the outcome model. A log‐logistic distribution was selected to model the survival time, following a comparison of the goodness‐of‐fit among exponential, Weibull, log‐normal, and log‐logistic distributions, as assessed by the Bayesian information criterion (BIC; See Appendix 4 in the Online Supporting Information). The outcomes derived from this regression model are interpreted as differences in the expected log‐years of survival following breast cancer diagnosis. The covariates were classified in the same categories as those presented in Table 5. The percentile nonparametric bootstrap with 1000 replications was employed to obtain 95% confidence intervals when estimating the treatment using a logistic regression, whereas the m‐out‐of‐n bootstrap was employed to obtain 95% confidence intervals with 200 replications when using SL.

### 6.3 Results

Table 5 reveals an imbalance between patients who received (67%) and those who did not receive (33%) hormonal therapy, suggesting a potential for major confounding bias if these differences are not adequately adjusted for. Among others, patients under hormonal therapy more often had a HR positive cancer, were generally older, had greater BMI, and exhibited higher cancer grade.

The results of the ATS analysis are presented in Table 6. We observed similarities between the estimations of the Logit and SL models. In both cases, the administration of hormonal therapy showed a potential increase in survival time among HR+ women with a normal BMI; however, the confidence intervals included zero, indicating that the results were inconclusive (Logit: 0.167, 95% CI: −0.004 to 0.332; SL: 0.162, 95% CI: −0.020 to 0.343). A similar pattern was observed for HR+ women with BMI ≥ 25, where both models estimated a positive effect (Logit: 0.155, 95% CI: −0.049 to 0.335; SL: 0.158, 95% CI: −0.034 to 0.351), but the confidence intervals again spanned zero, preventing definitive conclusions. For HR− women, regardless of BMI and the model used (Logit or SL), the results were inconclusive, with 95% confidence intervals showing the possibility of both beneficial and detrimental estimated effects of hormonal therapy.

**TABLE 6:** Estimated log‐years of survival differences (and 95% confidence intervals) between hormonal therapy and no hormonal therapy among female breast cancer patients according to categories of body mass index (BMI) and hormone receptor status (HR).

Estimator	HR+, BMI < 25 ${\text{kg/m}}^{2}$	HR+, BMI ≥ 25 ${\text{kg/m}}^{2}$	HR−, BMI < 25 ${\text{kg/m}}^{2}$	HR−, BMI ≥ 25 ${\text{kg/m}}^{2}$
Logit	0.167 (−0.004, 0.332)	0.155 (−0.049, 0.335)	−0.016 (−0.630, 0.665)	−0.136 (−0.603, 0.341)
SL	0.162 (−0.020, 0.343)	0.158 (−0.034, 0.351)	−0.025 (−0.608, 0.557)	−0.056 (−0.479, 0.367)

*Note*. Logit: Standard logistic regression; SL: Ensemble SuperLearner.

## 7 Discussion

In this study, we explored an approach aimed at enhancing the robustness of estimates for optimal ATSs. Specifically, we proposed using machine learning methods for modeling the treatment probability in the dWOLS estimator of optimal ATSs. Our motivation stems from recognizing the challenge of adequately controlling confounding factors for valid estimation of an optimal ATS when using observational data. We have further proposed employing cross‐fitting techniques to avoid overfitting and an adaptive m‐out‐of‐n bootstrap procedure for inferences. To facilitate the implementation of our proposal, R functions are available via GitHub (https://github.com/kosstre20/MachineLearningToControlConfoundingPersonalizedMedicine.git), allowing practitioners to apply the proposed models in their own research and analyses.

We have evaluated the performance of our proposed approach in various simulation scenarios, where the complexity of the true data‐generating equations for the treatment and outcome varied. SL generally performed as well as, or even better than, parametric logistic regression, exhibiting lower biases and standard deviations in specific scenarios, even in the presence of a lack of positivity or overlap in covariates' distribution across treatment groups, or with more covariates. The performance of our proposed approach with SL was similar, and sometimes even superior, to that of RQL, an alternative estimator of optimal ATSs that employs machine learning together with cross‐fitting. On the other hand, SVM, naive Bayes, RF, and neural networks sometimes showed significant biases or substantially increased standard deviations. The m‐out‐of‐n bootstrap appeared as both a theoretically and empirically valid approach for obtaining inferences when using machine learning to model the treatment probability. Recognizing the computation burden of using the m‐out‐of‐n bootstrap procedure, we thus recommend routinely using SL, together with twofold cross‐fitting, for estimating the treatment model when using the dWOLS estimator of an optimal ATS, at least as a sensitivity analysis for the point estimates.

Based on these results, we used dWOLS with the treatment modeled using SL to estimate the optimal ATS in the context of hormone therapy for women with breast cancer, and compared the results with those obtained when modeling the treatment using a logistic regression. In this illustration, both methods produced similar results, suggesting that the logistic regression model for the treatment may be approximately well specified.

Some limitations of our work must also be recognized. Considering the greatly increased computational burden, we did not fine‐tune the hyperparameters of the machine learning algorithms when conducting our simulation studies, instead using the default values in most instances. Not fine‐tuning the hyperparameters is likely to have led to poorer performances and would therefore not affect our conclusion that machine learning can be useful to better control measured confounding. Regardless, we recommend users to pay attention to hyperparameters tuning in practice. Among others, guidelines for specifying an SL have recently been published and may be considered (Phillips et al. 2023). Another, and perhaps related, limitation is our inability to employ SVM in some simulation scenarios due to the errors we encountered. We also recognize that the computational burden of employing machine learning to model the treatment probability may be prohibitive in some applications. For instance, our bootstrap procedure in the real data illustration required approximately 48 h to complete.

In conclusion, our study supports that using machine learning to model treatment probabilities can improve the robustness of estimates of optimal ATS. The SuperLearner was observed to reduce bias without increasing variance, thus improving the validity of estimates of optimal ATS. Drawing on machine learning methods and providing R functions accessible via GitHub, we offer practitioners practical tools to effectively implement our approach in their research.

## Conflicts of Interest

The authors declare no conflicts of interest.

## Open Research Badges

This article has earned an Open Data badge for making publicly available the digitally‐shareable data necessary to reproduce the reported results. The data is available in the Supporting Information section.

This article has earned an open data badge “**Reproducible Research**” for making publicly available the code necessary to reproduce the reported results. The results reported in this article were reproduced partially due to data confidentiality issues.

## Supporting information


**Supporting file 1:** bimj70068‐sup‐0001‐DataCode.zip;

**Supporting file 2:** bimj70068‐sup‐0002‐SuppMat.pdf;

**Supporting file 3:** bimj70068‐sup‐0003‐SuppMat.pdf

## Data Availability

The data that support the findings of this study are available on reasonable request from the corresponding author. The data are not publicly available due to legal restrictions to respect research participant privacy and consent.
