# Supplementary material for: Using Machine Learning to Improve Control for Confounding in the Dynamic Weighted Ordinary Least Squares Estimator of Optimal Adaptive Treatment Strategies
Source: Biom J. 2025 Jul 29;67(4):e70068. doi: 10.1002/bimj.70068 (PMC12305482; doi:10.1002/bimj.70068)
Supplement: Supplementary file 1 — Supporting file 1: bimj70068‐sup‐0001‐DataCode.zip; [file BIMJ-67-e70068-s002.zip › MachineLearningToControlConfoundingPersonalizedMedicine-main/README_Supplementary_Information.pdf]

README - Supplementary Information

Using machine learning to improve control for confounding in the dynamic weighted ordinary least squares estimator of optimal adaptive treatment strategies

Authors: Kossi Clément Trenou, Miceline Mésidor, Aida Eslami, Hermann Nabi, Caroline Diorio, and Denis Talbot

Corresponding Author for Code:

For questions, comments or remarks about the code, please contact Denis Talbot, Université Laval, Québec, QC G1V 0A6, Canada (denis.talbot@fmed.ulaval.ca).

Configurations:

The code was written/evaluated in **R** with the following software versions:

```
R version 4.3.2 (2023-10-31 ucrt)
Platform: x86_64-w64-mingw32/x64 (64-bit)
Running under: Windows 10 x64 (build 19045)

Matrix products: default


locale:
[1] LC_COLLATE=English_Canada.utf8  LC_CTYPE=English_Canada.utf8
[3] LC_MONETARY=English_Canada.utf8 LC_NUMERIC=C
[5] LC_TIME=English_Canada.utf8

time zone: America/Toronto
tzcode source: internal

attached base packages:
[1] grid      splines   stats     graphics  grDevices datasets  utils     methods
[9] base

other attached packages:
 [1] openxlsx_4.2.8       dplyr_1.1.4          haven_2.5.4
 [4] survey_4.4-2         survival_3.5-7       Matrix_1.6-1.1
 [7] tableone_0.13.2      resample_0.6         earth_5.3.4
[10] plotmo_3.6.4         plotrix_3.8-4        Formula_1.2-5
[13] MASS_7.3-60          nnet_7.3-19          e1071_1.7-16
[16] SuperLearner_2.0-29  gam_1.22-5           foreach_1.5.2
[19] nnls_1.6             randomForest_4.7-1.2

loaded via a namespace (and not attached):
 [1] compiler_4.3.2    renv_1.1.4        zip_2.3.2         tidyselect_1.2.1
 [5] Rcpp_1.0.14       lattice_0.21-9    R6_2.6.1          generics_0.1.3
 [9] iterators_1.0.14  forcats_1.0.0     tibble_3.2.1      DBI_1.2.3
[13] pillar_1.10.2     rlang_1.1.6       stringi_1.8.7     cli_3.6.4
[17] magrittr_2.0.3    class_7.3-22      hms_1.1.3         lifecycle_1.0.4
[21] vctrs_0.6.5       proxy_0.4-27      glue_1.8.0        codetools_0.2-19
[25] mitools_2.4       tools_4.3.2       pkgconfig_2.0.3
```

Code execution:

First note that we ran into multiple R crashes due to 'fatal errors' when conducting this work. These errors seem to occur randomly (i.e., they are not specific to a given state of the pseudo-random number generator). We believe these are related to memory issues, as they seem to occur less frequently on computers with better performance. As such, we have recorded the state of the pseudo-random number generator for each replication in scenarios where errors occurred more frequently (i.e., in simulations using Robust Q-Learning; RQL), allowing us to restart the simulation where it ended when crashes occurred. Unfortunately, running all simulations may therefore require many manual interventions.

The Rcodes folder contains the following data and files that can be used to reproduce all analyses and figures of the manuscript.

load_packages.R

This script contains the list of R packages required to run the simulations and analyses described in this repository. It includes a function that automatically checks if each package is installed, installs it if necessary, and then loads it into the R session.

run_simulation_analysis_all.R

A script containing the functions used to compute the performance measures described in Section 4.3. These functions are called in the scripts of each study described below. These functions can also be used to summarize the intermediate (raw) results.

./Study 1/:

- `SimulationsTwoTimesStudy1.R`
  A dedicated R script performs the simulations for Study 1, as described in Section 4 of the manuscript and in Appendix 1 of the supplementary material. The script covers the Standard Logistic Regression (Logit), Random Forest (RF), Naive Bayes (Bayes), Support Vector Machines (SVM), and Ensemble SuperLearner (SL) models. The simulation results are automatically saved in the *./Intermediate_results_study1/* directory, as the full simulation may require several hours of computation. Before running this script, the user needs to indicate the working directory manually at the top of the script file and run `load_packages.R`.

- `RQL_TwoTimes_Study1.R`
  A separate script is provided for the simulations involving the Robust Q-learning (RQL) since crashes were frequent for this method. The script follows essentially the same structure as above. However, because crashes may be more frequent, it might be preferable to run a single scenario at a time instead of trying to loop over all possible scenarios. The user then needs to indicate the sample size (300 or 1000) on line 129 and the scenario ("simple", "medium" or "complex") on line 131. When crashes occur, the user should remove the comment symbol (#) from the following lines

```
line 22:     if(i < ...) next;
line 122:    load("...")
line 133:    .Random.seed = seeds[[...]];
```

and indicate in lines 22 and 133 above the iteration index at which the code previously crashed and the name of the file corresponding to the scenario in line 122 before running the again script, effectively allowing to restart the simulation at the iteration before the crash. These lines should be commented again when running a new scenario from the first iteration.

- `./Intermediate_results_study1/`
  A folder containing the simulation results generated by *SimulationsTwoTimesStudy1.R* and *RQL_TwoTimes_Study1.R* for Study 1, for both sample sizes (300 and 1000) and across all scenarios.

- `Results_SimulationsTwoTimesStudy1.R`
  An R script that processes the .RData files stored in the *Intermediate_results_study1/* subfolder to generate the tables of results. These tables summarize the performance measures corresponding to Table 1 in Section 5 of the main manuscript and Table 1 of Appendix 1 Section 1 of the supplementary material. It also produces parts of the results presented in Table 5 of the supplementary material.

The structure and purpose of simulation scripts and results' folders for Studies 2–4 mirror those described in detail for Study 1. The simulation studies are described in Section 4 of the manuscript and in Appendix 1 of the supplementary material.

./Study 2/:

- `SimulationsTwoTimesStudy2.R`

- `RQL_TwoTimes_Study2.R`
  The user needs to indicate the sample size (300 or 1000) on line 152 and the scenario ("simple", "medium" or "complex") on line 153. If a crash occurs, the user needs to delete the comment symbol (#) from the following lines

```
line 24:     if(i < ...) next;
line 145:    load("...")
line 155:    .Random.seed = seeds[[...]];
```

  and indicate in lines 24 and 155 above the iteration index at which the code previously crashed and the name of the file corresponding to the scenario in line 145 before running again the script. These lines should be commented again when running a new scenario from the first iteration.

- `./Intermediate_results_study2/`

- `Results_SimulationsTwoTimesStudy2.R`
  These tables summarize the performance measures corresponding to Table 2 in Section 5 of the main manuscript (sample size 300) and Table 2 in Appendix 1 of the supplementary material (sample size 1000) and Table 5 (Monte Carlo standard error).

./Study 3/:

- `SimulationsTwoTimesStudy3.R`

- RQL_TwoTimes_Study3.R
  The user needs to indicate the sample size (300 or 1000) on line 151 and the scenario ("simple", "medium" or "complex") on line 152. If a crash occurs, the user needs to delete the comment symbol (#) from the following lines

  ```
  line 23:     if(i < ...) next;
  line 144:    load("...")
  line 155:    .Random.seed = seeds[[...]];
  ```

  and indicate in lines 23 and 155 above the iteration index at which the code previously crashed and the name of the file corresponding to the scenario in line 144 before running again the script. These lines should be commented again when running a new scenario from the first iteration.

- ./Intermediate_results_study3/

- Results_SimulationsTwoTimesStudy3.R
  These tables summarize the performance measures corresponding to Table 3 in Section 5 of the main manuscript (sample size 300) and Table 3 in Appendix 1 of the supplementary material (sample size 1000) and Table 5 (Monte Carlo standard error).

./Study 4/:

- SimulationsTwoTimesStudy4.R

- RQL_TwoTimes_Study4.R
  The user needs to indicate the sample size (300 or 1000) on line 152 and the scenario ("simple", "medium" or "complex") on line 153. If a crash occurs, the user needs to delete the comment symbol (#) from the following lines

  ```
  line 23:     if(i < ...) next;
  line 146:    load("...")
  line 156:    .Random.seed = seeds[[...]];
  ```

  and indicate in lines 23 and 156 above the iteration index at which the code previously crashed and the name of the file corresponding to the scenario in line 146 before running again the script. These lines should be commented again when running a new scenario from the first iteration.

- ./Intermediate_results_study4/

- Results_SimulationsTwoTimesStudy4.R
  These tables summarize the performance measures corresponding to Table 4 in Section 5 of the main manuscript (sample size 300) and Table 4 in Appendix 1 of the supplementary material (sample size 1000) and Table 5 (Monte Carlo standard error).

./Plot_Positivity_Studies_1_4/

- `Boxplot_Posivity_Distribution_Studies_1_3.R`
  An R script used to generate Figure 1 (A) and Figure 2 of the supplementary material of the manuscript. The directory where to save the figure files must be manually indicated on line 36 (`figure_dir = ...`).

- `Boxplot_Posivity_Distribution_Study_4.R`
  An R script used to generate Figure 1(B) and Figures 3 to 6 in the supplementary material of the manuscript. The directory where to save the figure files must be manually indicated on line 5 (`figure_dir = ...`).

- `./Figures/`
  A folder containing the plot results generated by *Boxplot_Positivity_Distribution_Studies_1_3.R* and *Boxplot_Positivity_Distribution_Study_4.R*, as presented in the supplementary material of the manuscript.

./Study 5/:

The simulation studies for Study 5 are described in the supplementary material, Appendix 2.

- `SimulationsLargeDimensionalityTwoTimesStudy5.R`

- `RQLStudy5.R`
  The user needs to indicate the sample size (300 or 1000) on line 247 and the scenario ("simple", "medium" or "complex") on line 248. If a crash occurs, the user needs to delete the comment symbol (#) from the following lines

  ```
  line 25:     if(i < ...) next;
  line 241:    load("...")
  line 250:    .Random.seed = seeds[[...]];
  ```

  and indicate in lines 25 and 250 above the iteration index at which the code previously crashed and the name of the file corresponding to the scenario in line 241 before running again the script. These lines should be commented again when running a new scenario from the first iteration.

- `./Intermediate_results_study5/`

- `Result_SimulationsLargeDimensionalityTwoTimesStudy5.R`
  An R script processes the .RData files stored in the *./Intermediate_results_study5/* subfolder to generate the result tables included in the online supplementary materials of the manuscript. These tables summarize the performance measures corresponding to Table 6-11 in Appendix 2 of the supplementary material and Table 12 for the Monte Carlo standard error.

- `PlotStudy5.R`
  An R script used to generate Figures 7 to 14 in the supplementary material of the manuscript.

- `./Figures/`
  A folder containing the figures generated by *PlotStudy5.R*, as presented in the supplementary material of the manuscript.

./m-out-of-n bootstrap Study 4/:

- `Boostrap.R`
  This script provides the implementation of the m-out-of-n bootstrap procedure described in Section 3.1 of the main text. It is specifically designed for Study 4.

- `./Intermediate_results_Boostrap/`
  This folder contains the intermediate results generated by the *Boostrap.R* script. It includes *.RData* files summarizing the bootstrap replicates for each configuration of Study 4.

- `Results_Boostrap.R`
  This script takes the *.RData* files from the *Intermediate_results_Boostrap/* subdirectory and processes them to generate summary statistics related to the bootstrap results. These summaries correspond to the analyses presented in Section 6 of the manuscript and are used to assess the performance and coverage properties of the m-out-of-n bootstrap estimators.

./Two_to_Five_Folds_Study_4/

- `Two_to_Five_Folds_Study_4.R` A script to investigate the number of folds in cross-fitting, as described in Section 4.1 of the main text.

- `./Intermediate_results_Two_to_Five_Folds_Study_4/` A subfolder containing the intermediate `.RData` files generated by `Two_to_Five_Folds_Study_4.R`.

- `Results_Two_to_Five_Folds_Study_4.R`
  An R script processes the `.RData` files stored in the `./Intermediate_results_Two_to_Five_Folds_Study_4/` subfolder to generate the result tables presented in Appendix 3 of the supplementary materials of the manuscript (Tables 13 and 14).

./SimulationData_ApplicationScript_Estimate/:

This folder contains files pertaining to the real data analysis. For confidentiality reasons, we are not allowed to share the real data publicly. As described below, a simulated dataset that has the same structure and size as the original real dataset is however made available. All scripts described below to analyze the real data can also be executed on these simulated data, although the results will not be the same.

- `./Results_Simulation_data/`
  A subfolder containing the file *Simulation_data_Application.csv*. This is the simulated dataset that replicates several features of the real dataset. It must be loaded into each of the following scripts in order to perform the corresponding analyses: *Descriptive_statistics.R*, *Application_Logit.R*, and *Application_SL.R*.

- `Descriptive_statistics.R`
  A script for analyzing and summarizing the real data, as presented in Table 5 of the main manuscript.

- `Application_Logit.R`
  This script is applied to the real dataset using Logistic Regression (Logit) to appropriately account for censoring, as described in Section 6.2 of the main manuscript. The results

are presented in Table 15 of Appendix 4 in the supplementary material. Additionally, the script is used to compute the point estimates and confidence intervals reported in Table 6 of the main manuscript.

- `Application_SL.R`
  This script is applied to the real dataset using Ensemble SuperLearner (SL) to appropriately account for censoring, as described in Section 6.2 of the main manuscript. The results are presented in Table 15 of Appendix 4 in the supplementary material. Additionally, the script is used to compute the point estimates and confidence intervals reported in Table 6 of the main manuscript.

- `./Intermediate_Results_Application_data/`
  A subfolder containing the intermediate *.RData* files generated from the bootstrap procedure for both the Logit and Super Learner (SL) estimators, along with the execution time recorded from the corresponding scripts *Application_Logit.R* and *Application_SL.R*.
