# Supplementary figures and images for: Using Machine Learning to Improve Control for Confounding in the Dynamic Weighted Ordinary Least Squares Estimator of Optimal Adaptive Treatment Strategies
Source: Biom J. 2025 Jul 29;67(4):e70068. doi: 10.1002/bimj.70068 (PMC12305482; doi:10.1002/bimj.70068)

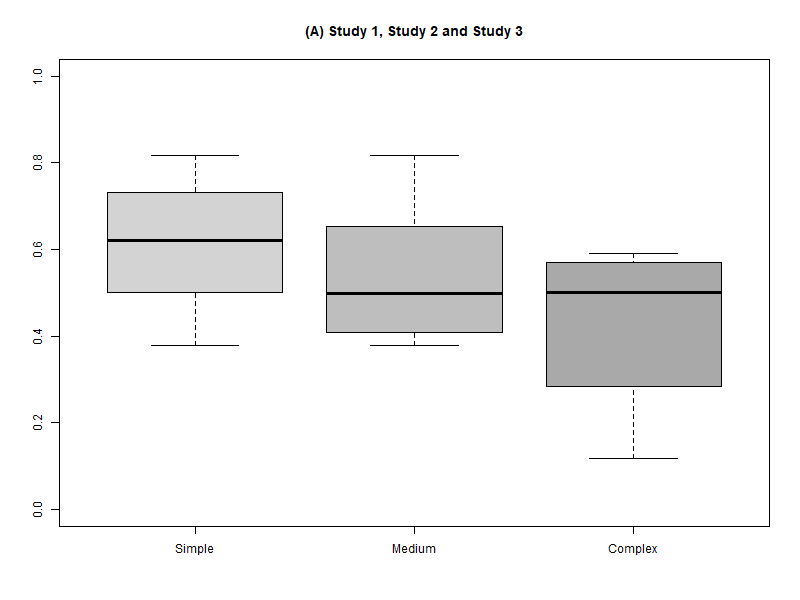

Supplement: Supplementary file 1 — Supporting file 1: bimj70068‐sup‐0001‐DataCode.zip; [file BIMJ-67-e70068-s002.zip › MachineLearningToControlConfoundingPersonalizedMedicine-main/Plot_Positivity_Studies_1_4/Figures/Boxplot_probabilities_Study1_3.png]

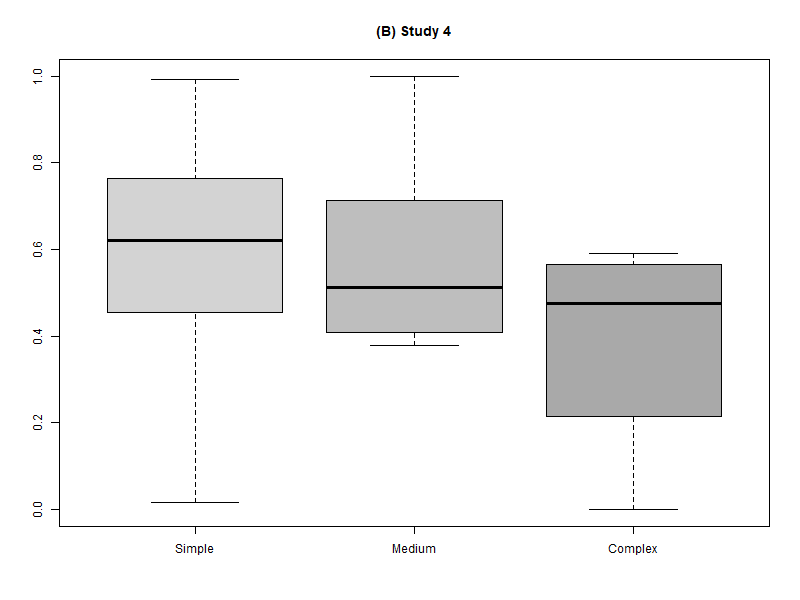

Supplement: Supplementary file 1 — Supporting file 1: bimj70068‐sup‐0001‐DataCode.zip; [file BIMJ-67-e70068-s002.zip › MachineLearningToControlConfoundingPersonalizedMedicine-main/Plot_Positivity_Studies_1_4/Figures/Boxplot_probabilities_Study_4.png]

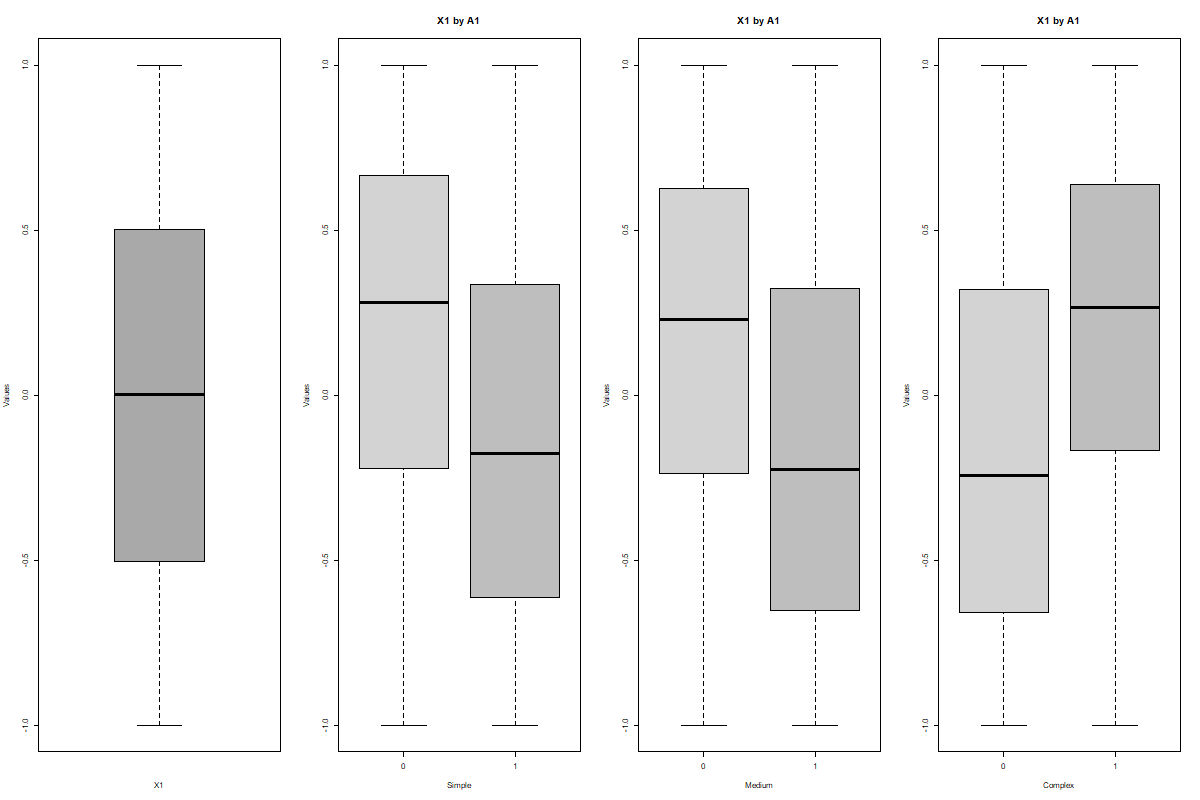

Supplement: Supplementary file 1 — Supporting file 1: bimj70068‐sup‐0001‐DataCode.zip; [file BIMJ-67-e70068-s002.zip › MachineLearningToControlConfoundingPersonalizedMedicine-main/Plot_Positivity_Studies_1_4/Figures/Distribution_X1_studies1_3_by_A.png]

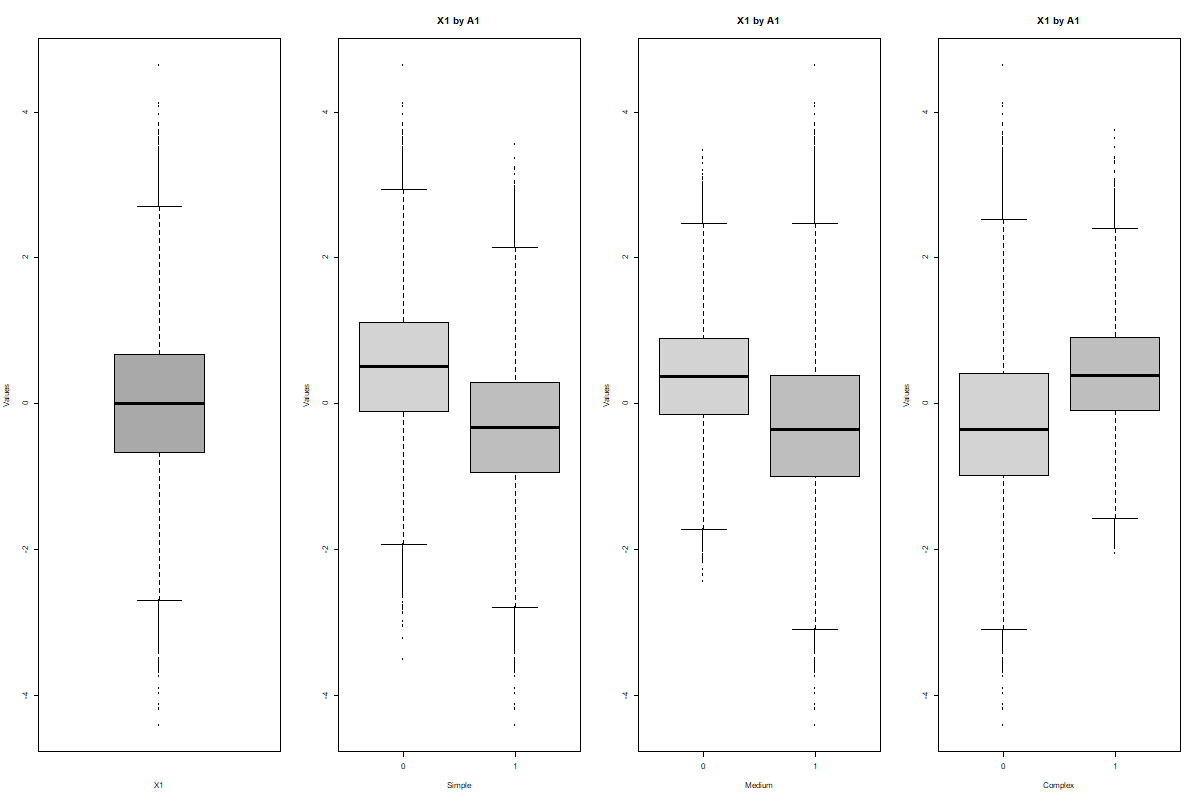

Supplement: Supplementary file 1 — Supporting file 1: bimj70068‐sup‐0001‐DataCode.zip; [file BIMJ-67-e70068-s002.zip › MachineLearningToControlConfoundingPersonalizedMedicine-main/Plot_Positivity_Studies_1_4/Figures/Distribution_X1_study_4_by_A.png]

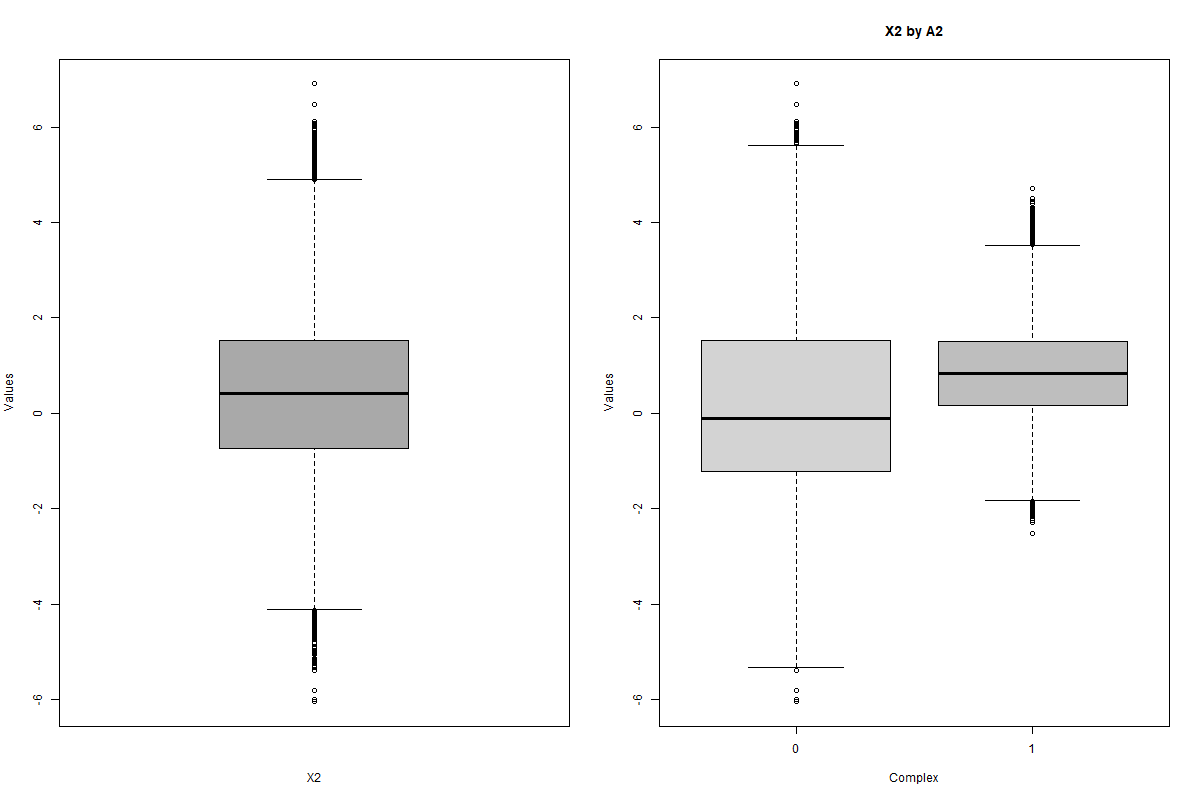

Supplement: Supplementary file 1 — Supporting file 1: bimj70068‐sup‐0001‐DataCode.zip; [file BIMJ-67-e70068-s002.zip › MachineLearningToControlConfoundingPersonalizedMedicine-main/Plot_Positivity_Studies_1_4/Figures/Distribution_X2_study_4_by_A2_complex.png]

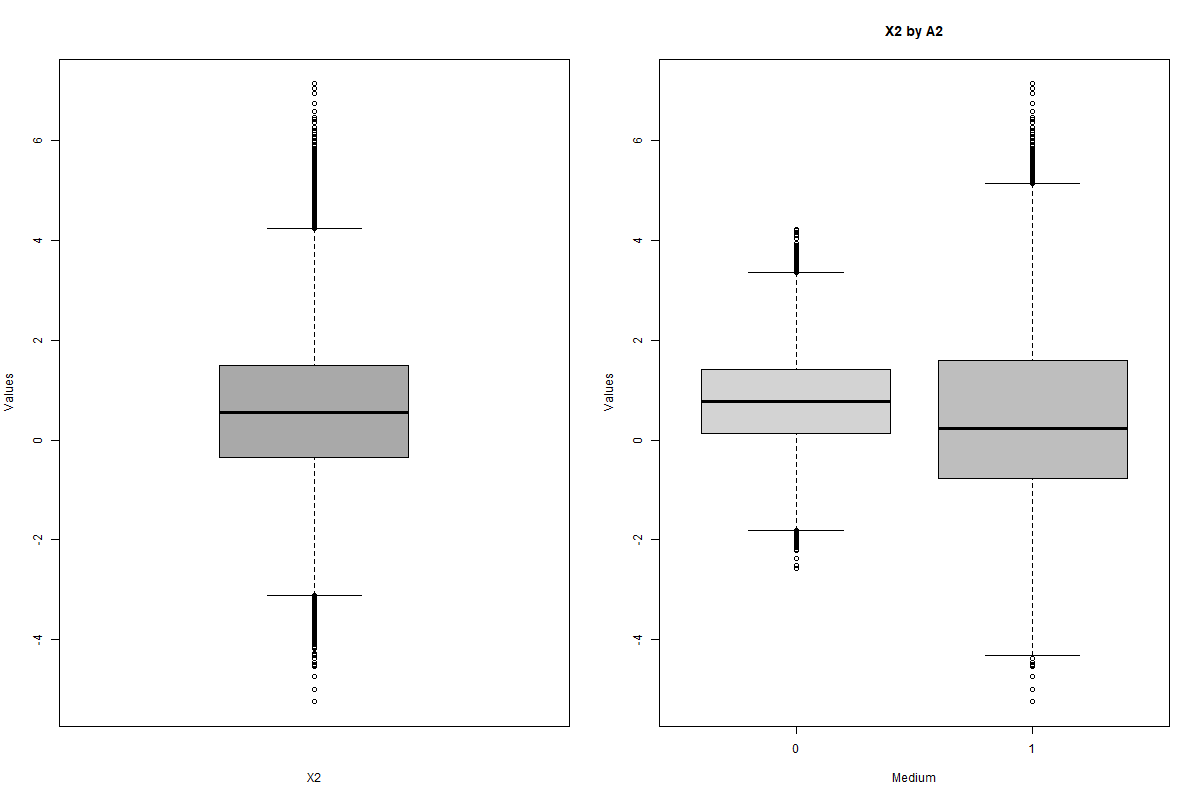

Supplement: Supplementary file 1 — Supporting file 1: bimj70068‐sup‐0001‐DataCode.zip; [file BIMJ-67-e70068-s002.zip › MachineLearningToControlConfoundingPersonalizedMedicine-main/Plot_Positivity_Studies_1_4/Figures/Distribution_X2_study_4_by_A2_medium.png]

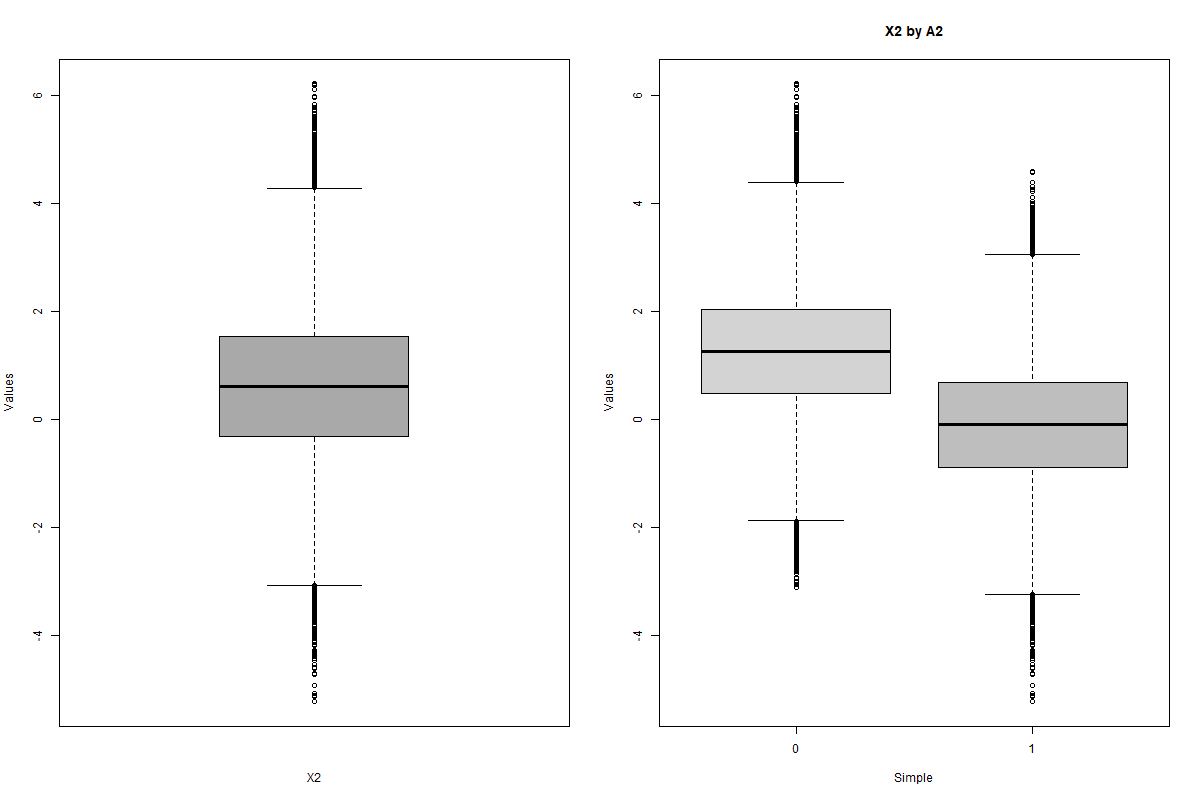

Supplement: Supplementary file 1 — Supporting file 1: bimj70068‐sup‐0001‐DataCode.zip; [file BIMJ-67-e70068-s002.zip › MachineLearningToControlConfoundingPersonalizedMedicine-main/Plot_Positivity_Studies_1_4/Figures/Distribution_X2_study_4_by_A2_simple.png]

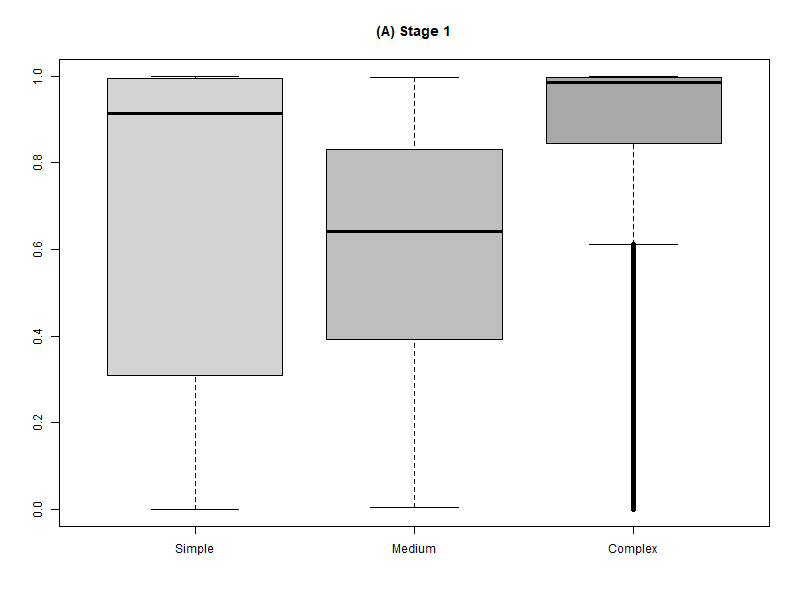

Supplement: Supplementary file 1 — Supporting file 1: bimj70068‐sup‐0001‐DataCode.zip; [file BIMJ-67-e70068-s002.zip › MachineLearningToControlConfoundingPersonalizedMedicine-main/Study 5/Figures/Distribution_A1_stage1.png]

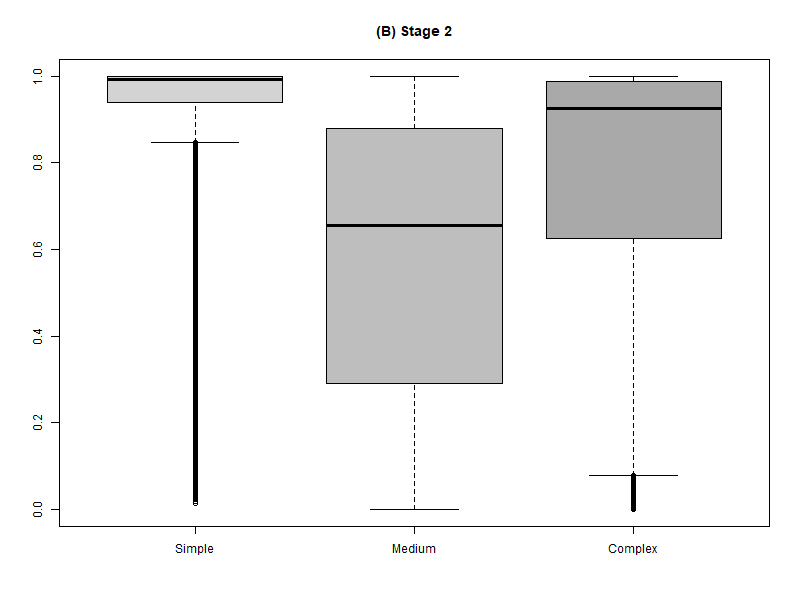

Supplement: Supplementary file 1 — Supporting file 1: bimj70068‐sup‐0001‐DataCode.zip; [file BIMJ-67-e70068-s002.zip › MachineLearningToControlConfoundingPersonalizedMedicine-main/Study 5/Figures/Distribution_A2_stage2.png]

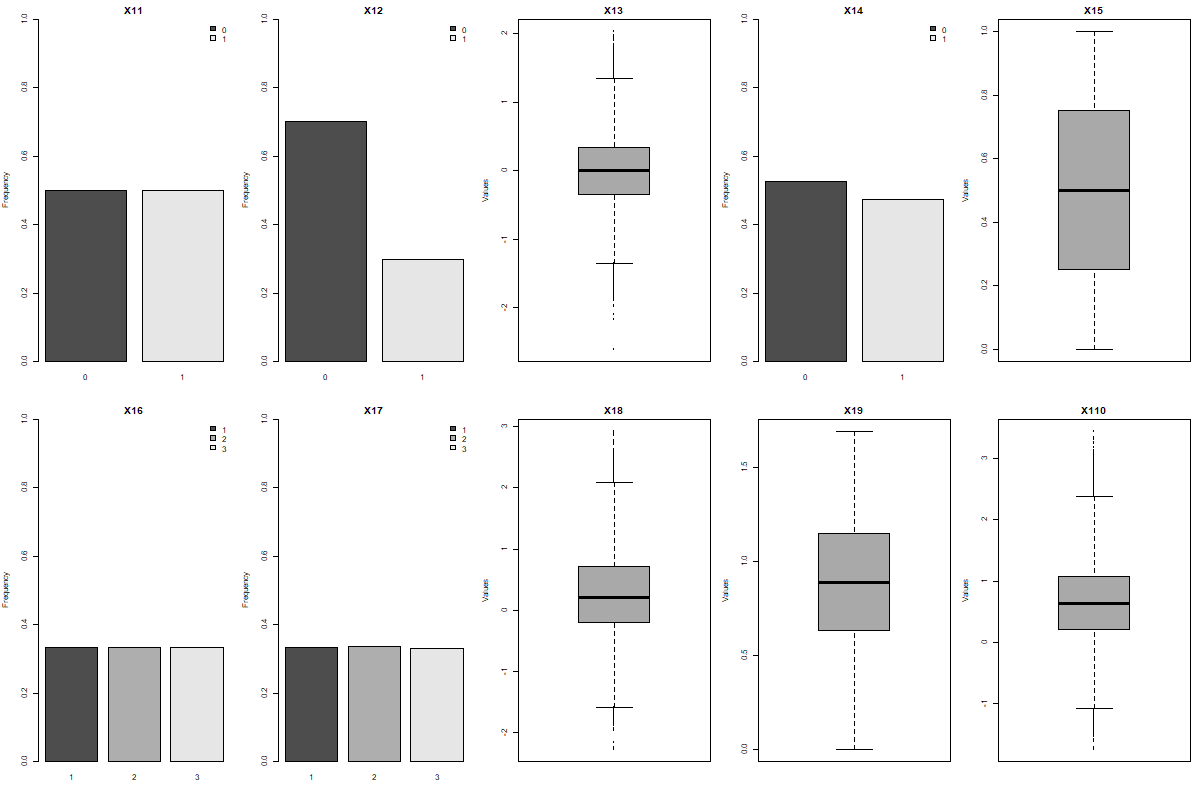

Supplement: Supplementary file 1 — Supporting file 1: bimj70068‐sup‐0001‐DataCode.zip; [file BIMJ-67-e70068-s002.zip › MachineLearningToControlConfoundingPersonalizedMedicine-main/Study 5/Figures/Distribution_X11_X10_all_scenarios.png]

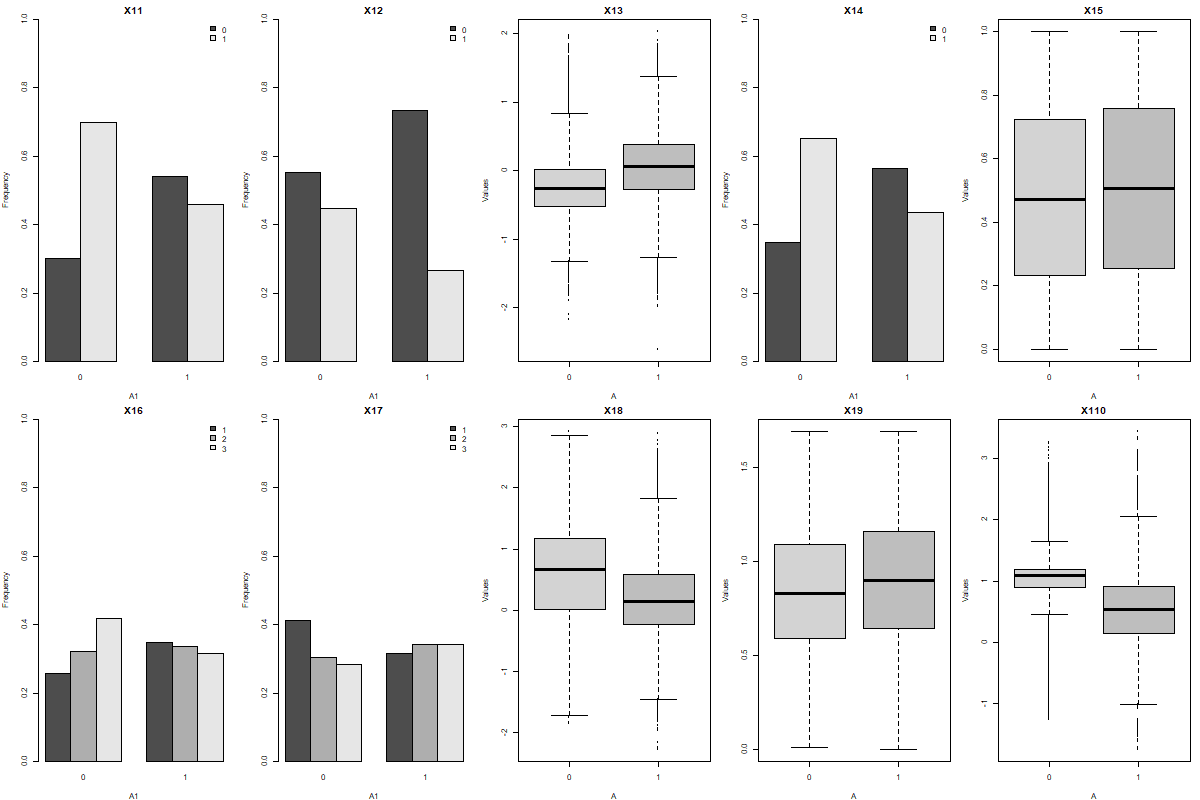

Supplement: Supplementary file 1 — Supporting file 1: bimj70068‐sup‐0001‐DataCode.zip; [file BIMJ-67-e70068-s002.zip › MachineLearningToControlConfoundingPersonalizedMedicine-main/Study 5/Figures/Distribution_X11_X10_by_A1_complex.png]

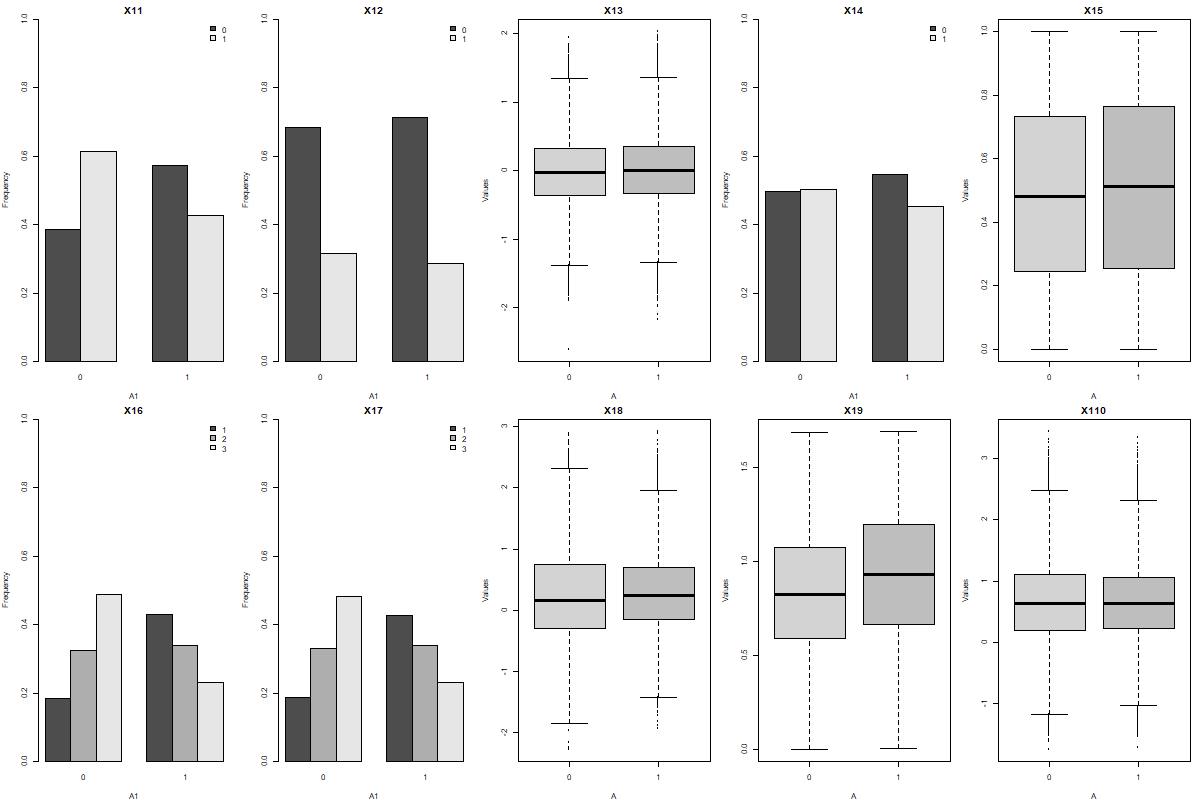

Supplement: Supplementary file 1 — Supporting file 1: bimj70068‐sup‐0001‐DataCode.zip; [file BIMJ-67-e70068-s002.zip › MachineLearningToControlConfoundingPersonalizedMedicine-main/Study 5/Figures/Distribution_X11_X10_by_A1_medium.png]

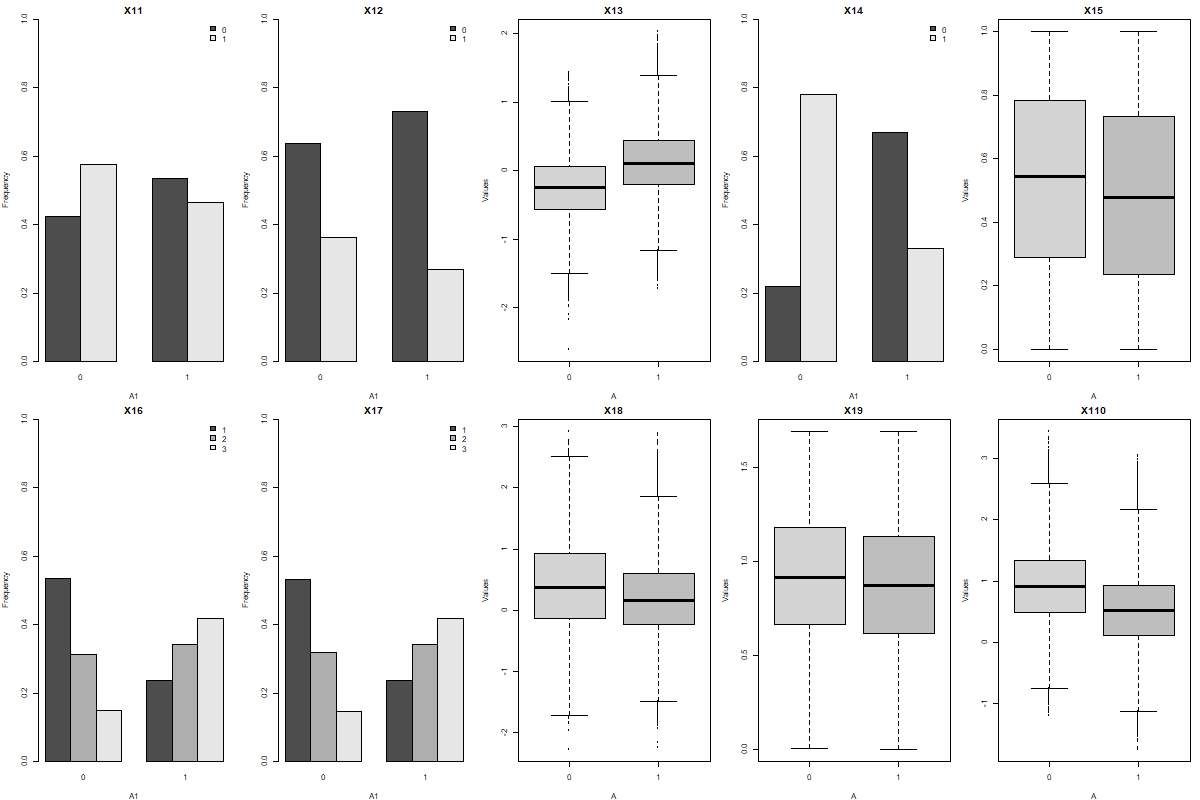

Supplement: Supplementary file 1 — Supporting file 1: bimj70068‐sup‐0001‐DataCode.zip; [file BIMJ-67-e70068-s002.zip › MachineLearningToControlConfoundingPersonalizedMedicine-main/Study 5/Figures/Distribution_X11_X10_by_A1_simple.png]

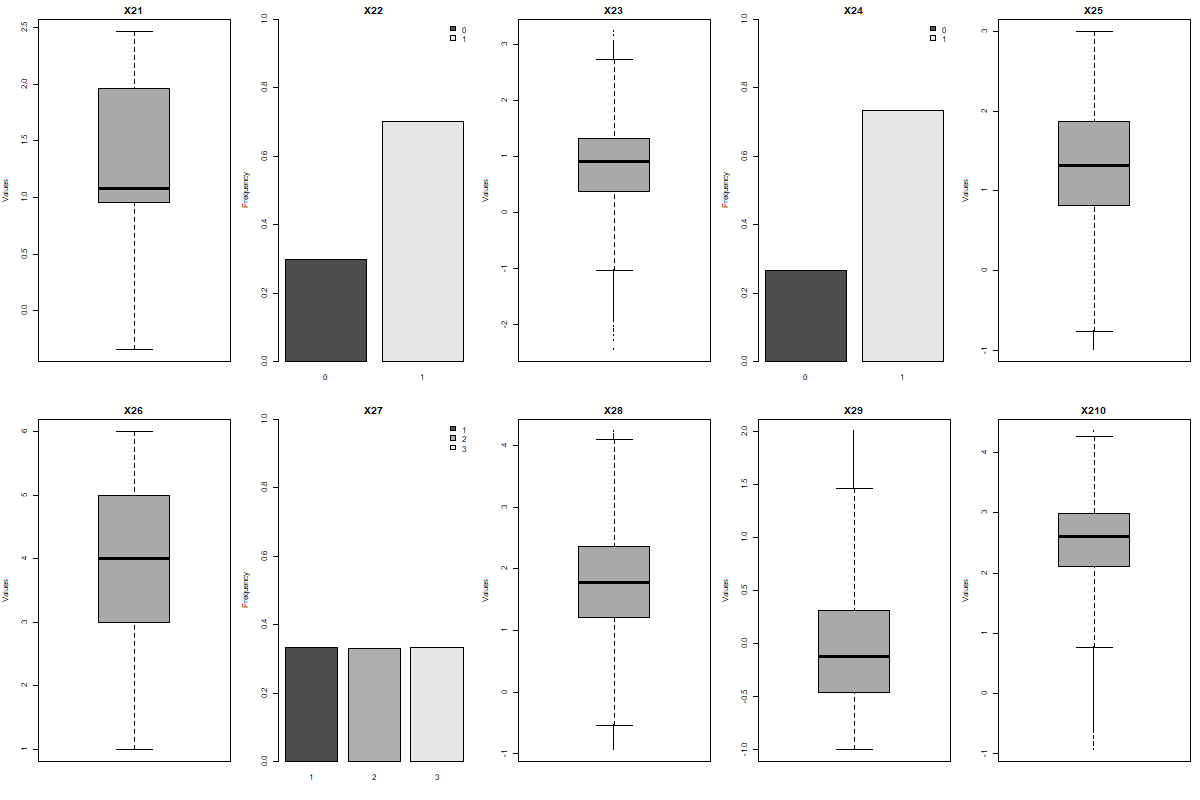

Supplement: Supplementary file 1 — Supporting file 1: bimj70068‐sup‐0001‐DataCode.zip; [file BIMJ-67-e70068-s002.zip › MachineLearningToControlConfoundingPersonalizedMedicine-main/Study 5/Figures/Distribution_X21_X210_complex.png]

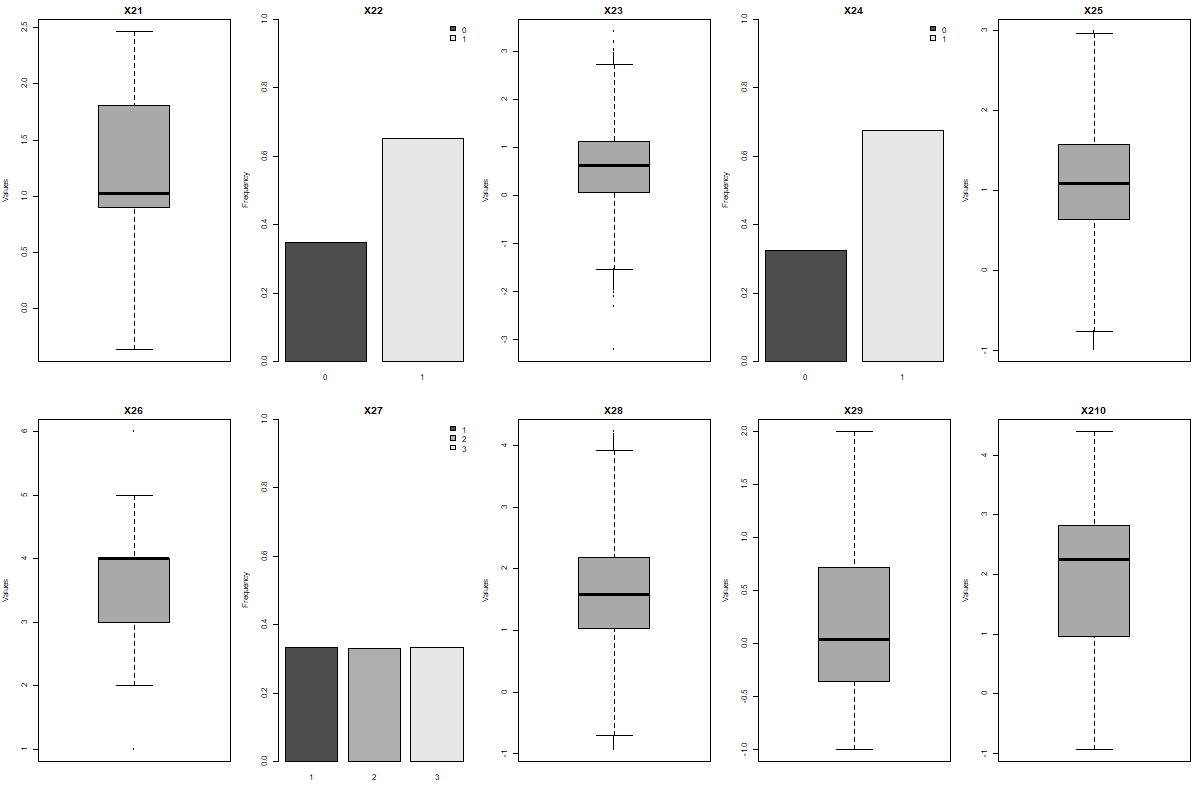

Supplement: Supplementary file 1 — Supporting file 1: bimj70068‐sup‐0001‐DataCode.zip; [file BIMJ-67-e70068-s002.zip › MachineLearningToControlConfoundingPersonalizedMedicine-main/Study 5/Figures/Distribution_X21_X210_medium.png]

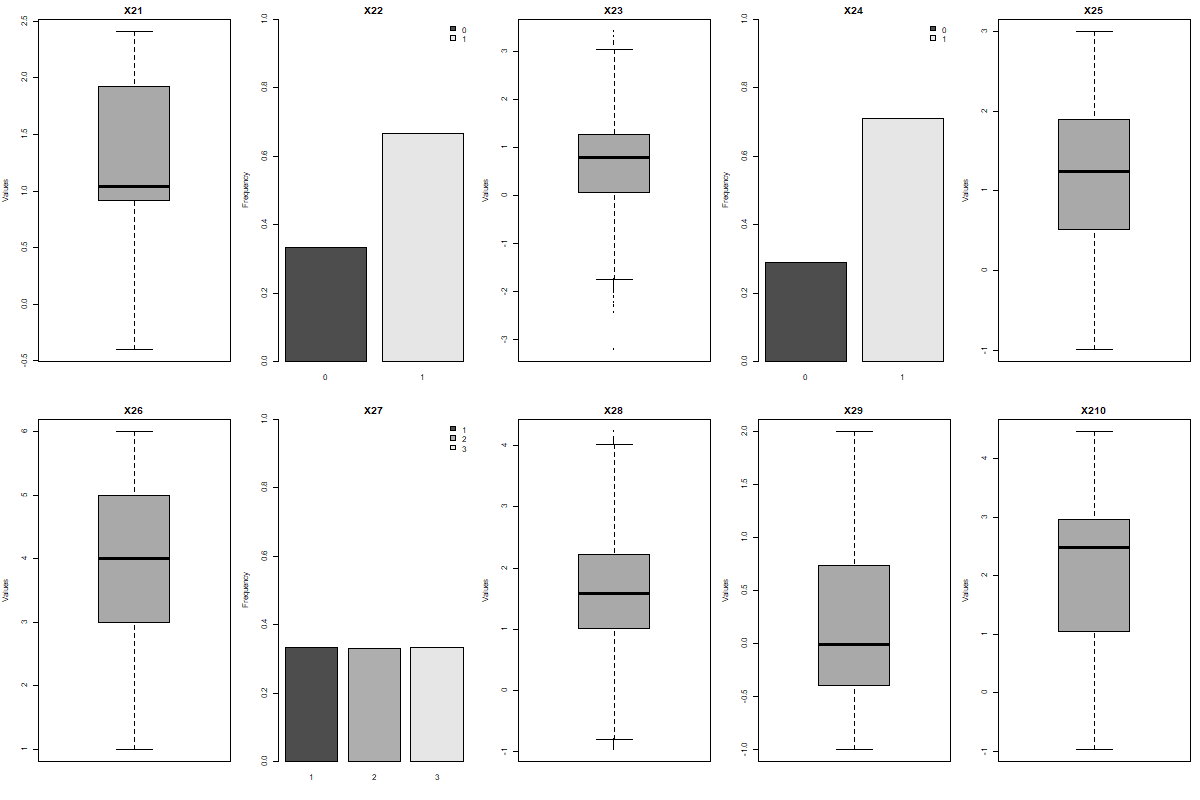

Supplement: Supplementary file 1 — Supporting file 1: bimj70068‐sup‐0001‐DataCode.zip; [file BIMJ-67-e70068-s002.zip › MachineLearningToControlConfoundingPersonalizedMedicine-main/Study 5/Figures/Distribution_X21_X210_simple.png]
