# Supplementary material for: Using Machine Learning to Improve Control for Confounding in the Dynamic Weighted Ordinary Least Squares Estimator of Optimal Adaptive Treatment Strategies
Source: Biom J. 2025 Jul 29;67(4):e70068. doi: 10.1002/bimj.70068 (PMC12305482; doi:10.1002/bimj.70068)
Supplement: Supplementary file 2 — Supporting file 2: bimj70068‐sup‐0002‐SuppMat.pdf; [file BIMJ-67-e70068-s001.pdf]

Supplementary material for "Using machine learning to improve control for confounding in the dynamic weighted ordinary least squares estimator of optimal adaptive treatment strategies" by Kossi Clément Trenou, Miceline Mésidor, Aida Eslami, Hermann Nabi, Caroline Diorio and Denis Talbot.

Appendix 1 – Additional simulation results with two time-points for Study 1-3 and Study 4

The history at the first time point was $H_1 = X_1$ in all studies and $H_2 = (X_1, A_1, X_2)$ at the second time point. Their generating equations in Studies 1-3 were $X_1 \sim \text{Uniform}(-1, 1)$ and $X_2 = X_1 + A_1 + \text{Uniform}(-1, 1)$; and in Study 4, $X_1 \sim N(0, 1)$ and $X_2 = X_1 + A_1 + N(0, 1)$. Note that generating the covariates at first time point and second time point according to a uniform distribution in Studies 1-3 prevents positivity issues by bounding the domain of these covariates and thus bounding the treatment probabilities away from 0 and 1, whereas practical positivity violations may occur in Study 4 when generating the covariates according to a normal distribution. In addition, for all studies $A_1^{opt} = I(L_1\psi_1 > 0)$; $\mu_1 = (A_1^{opt} - A1)L_1\psi_1$ and $A_2^{opt} = I(L_2\psi_2 > 0)$; $\mu_2 = (A_2^{opt} - A_2)L_2\psi_2$ where $I(\cdot)$ is the usual indicator function and $\psi_1 = \psi_2 = (-0.5, 1)$; $L_t = (1, X_t), t = 1, 2$. The other data-generating equations varied between studies and are described in the next paragraphs. At the second time point, the true values of the causal parameters are $(\psi_{20}^*, \psi_{21}^*) = (\psi_{20}, \psi_{21}) = (-0.5, 1.0)$. As will be seen shortly, because part of the effect of the treatment at the first time point passes through X_2, which is non-linearly related to the outcome in some scenarios, the true value of the effect at the first time point was determined using a Monte Carlo simulation of counterfactual outcomes in these scenarios. A sample of size 10,000,000 was used to minimize the Monte Carlo error. For each study, in scenarios where the outcome was generated according to a **simple** parametric equation, the true values of the parameters were $(\psi_{10}^*, \psi_{11}^*) = (\psi_{10}, \psi_{11}) = (0.5, 1.0)$; in scenarios where the outcome was generated according to parametric equations of **medium** complexity, the true values were $(\psi_{10}^*, \psi_{11}^*) \approx (1.5, 3.0)$; for scenarios where the outcome was generated according to **complex** parametric equations, the true values were $(\psi_{10}^*, \psi_{11}^*) \approx (0.5, 0.0)$.

In Study 1, the outcome variable was generated according to a simple parametric equation. As for the treatment variable, three scenarios with varying degree of complexity were designed. The treatment equations at the first and second time points were, respectively: **simple scenario** $A_1 \sim \text{Bernoulli}(p = \text{expit}(0.5 - X_1))$ and $A_2 \sim \text{Bernoulli}(p = \text{expit}(0.5 - X_2))$, where $\text{expit}(x) = \frac{\exp(x)}{1+\exp(x)}$; **medium scenario** $A_1 \sim \text{Bernoulli}(p = \text{expit}(-X_1 + 0.5X_1^2))$ and $A_2 \sim \text{Bernoulli}(p = \text{expit}(-X_2 + 0.5X_2^2))$; **complex scenario** $A_1 \sim \text{Bernoulli}(p = \text{expit}(1.2X_1 - 0.25X_1^2 - |X_1| + 0.5|\sin(X_1)|))$ and $A_2 \sim \text{Bernoulli}(p = \text{expit}(1.2X_2 - 0.25X_2^2 - |X_2| + 0.5|\sin(X_2)|))$. The outcome was generated as $Y \sim N(X_1 + X_2 - \mu_1 - \mu_2, 1)$.

For Study 2, the treatment variable was generated based on a simple parametric equation. As for the outcome variable, three scenarios were designed to vary the complexity of the outcome generation equations. The treatment equation at the first time was $A_1 \sim \text{Bernoulli}(p = \text{expit}(1 - 0.5X_1))$ and the second time was $A_2 \sim \text{Bernoulli}(p = \text{expit}(1 - 0.5X_2))$. The outcome equation was: **simple scenario** $Y \sim N(X_1 + X_2 - \mu_1 - \mu_2, 1)$; **medium scenario** $Y \sim N(X_1 + X_2 + X_1^2 + X_2^2 - \mu_1 - \mu_2, 1)$; **complex scenario** $Y \sim N(X_1 + X_2 - X_1X_2 - 0.1\sin(X_1) - \cos(\sin(X_1X_2)) + 0.5|\cos(X_1X_2)| - \mu_1 - \mu_2, 1)$.

In Study 3 and 4, both the treatment's and the outcome's generating equations were of varying complexity. The scenarios are defined as follows: **simple scenario** $A_1 \sim \text{Bernoulli}(p = \text{expit}(1 - 0.5X_1))$, $A_2 \sim \text{Bernoulli}(p = \text{expit}(1 - 0.5X_2))$ and $Y \sim N(X_1 + X_2 - \mu_1 - \mu_2, 1)$; **medium sce-**

nario $A_1 \sim \text{Bernoulli}(p = \text{expit}(-X_1 + 0.5X_1^2))$, $A_2 \sim \text{Bernoulli}(p = \text{expit}(-X_2 + 0.5X_2^2))$ and $Y \sim N(X_1 + X_2 + X_1^2 + X_2^2 - \mu_1 - \mu_2, 1)$; **complex scenario** $A_1 \sim \text{Bernoulli}(p = \text{expit}(1.2X_1 - 0.25X_1^2 - |X_1| + 0.5|\sin(X_1)))|$, $A_2 \sim \text{Bernoulli}(p = \text{expit}(1.2X_2 - 0.25X_2^2 - |X_2| + 0.5|\sin(X_2)|))$ and $Y \sim N(X_1 + X_2 - X_1X_2 - 0.1\sin(x_1) - \cos(\sin(X_1X_2)) + 0.5|\cos(X_1X_2)| - \mu_1 - \mu_2, 1)$.

A total of 1000 replicates were performed for each scenario of each study. Sample sizes of $n = 300$ and $n = 1000$ were used to investigate how the performance varied according to sample size. The results for the size $n = 300$ are reported in the main material.

Distribution of treatment probabilities and covariates for Studies 1-3 and Study 4

Figure 1 depicts the distribution of the treatment probabilities in each scenario of each study. In Studies 1-3, the probabilities are bounded away from 0 and 1, showcasing the lack of positivity violations. In Study 4, some probabilities are either very close to 0 or 1, illustrating the potential for practical positivity violations. The figures for Study 4 also illustrate some lack of overlap in the covariate' distribution across treatment groups.

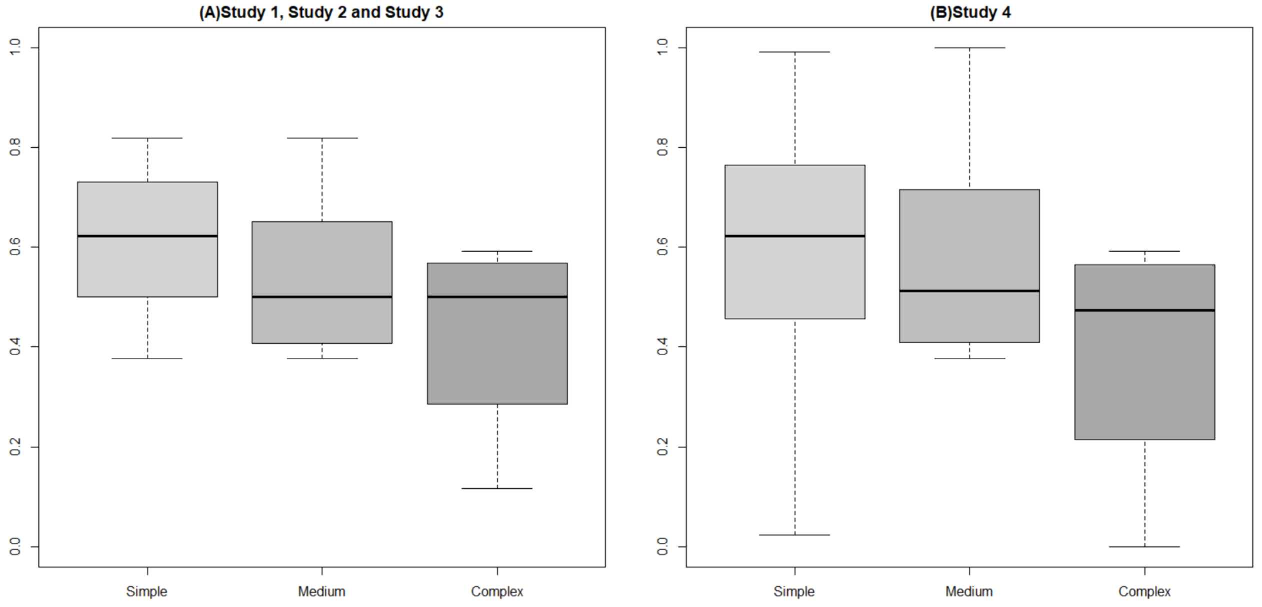

Figure 1: Distribution of the treatment probabilities (A) Studies 1-3, (B) Study 4.

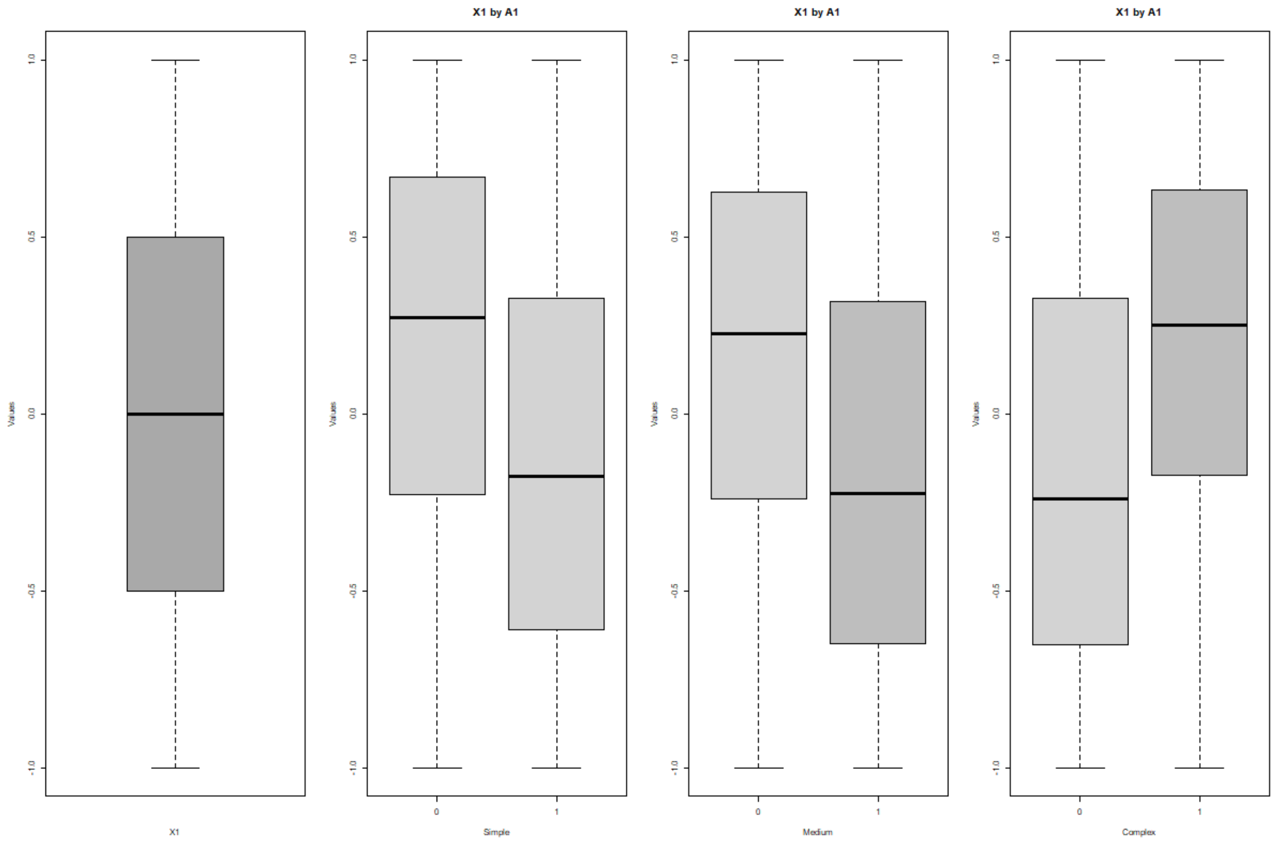

Figure 2: Study 1-3, Stage 1: Distribution of Covariates X_1 for all scenarios and Covariate by A_1 for each scenario

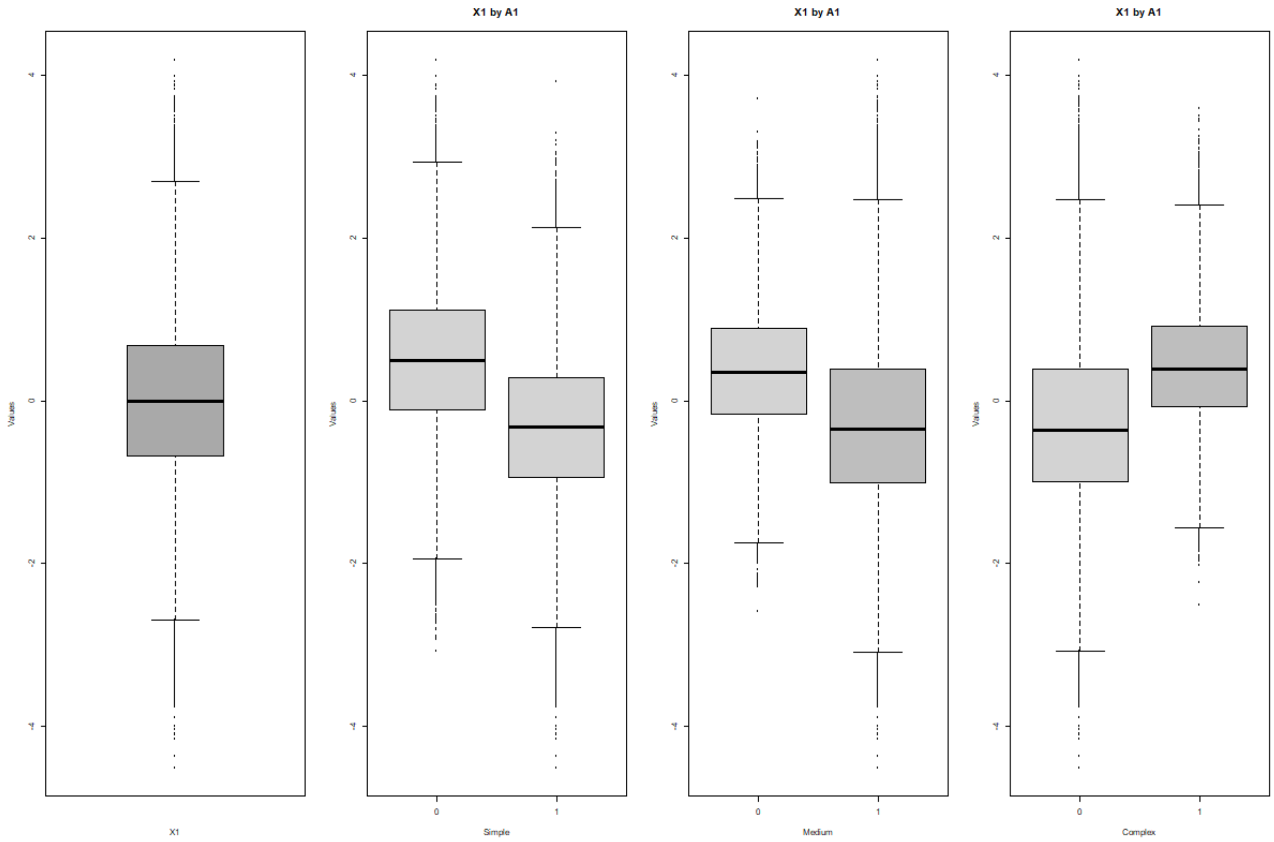

Figure 3: Study 4, Stage 1: Distribution of Covariates X_1 for all scenarios and Covariate by A_1 for each scenario

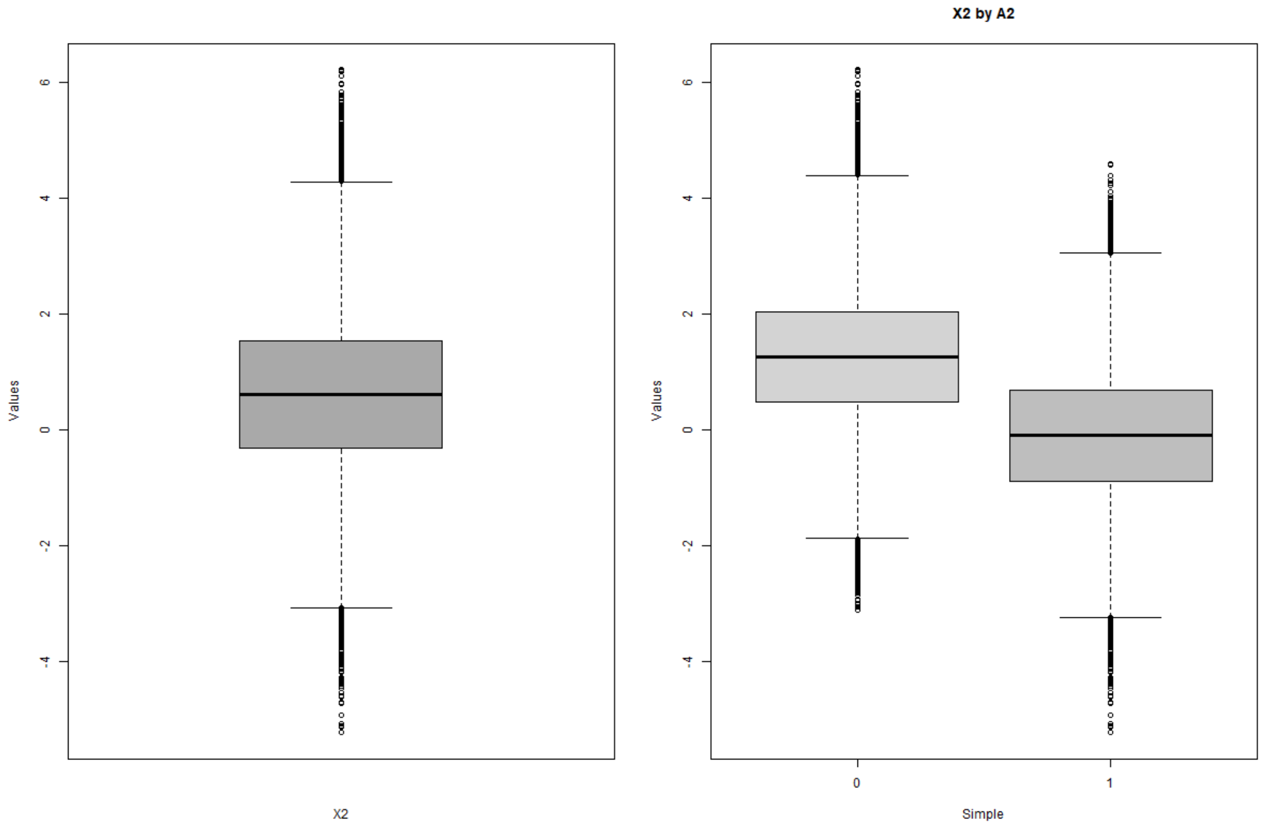

Figure 4: Study 4, Stage 2: Distribution of Covariates X_2 and Covariate X_2 by A_2, simple scenario

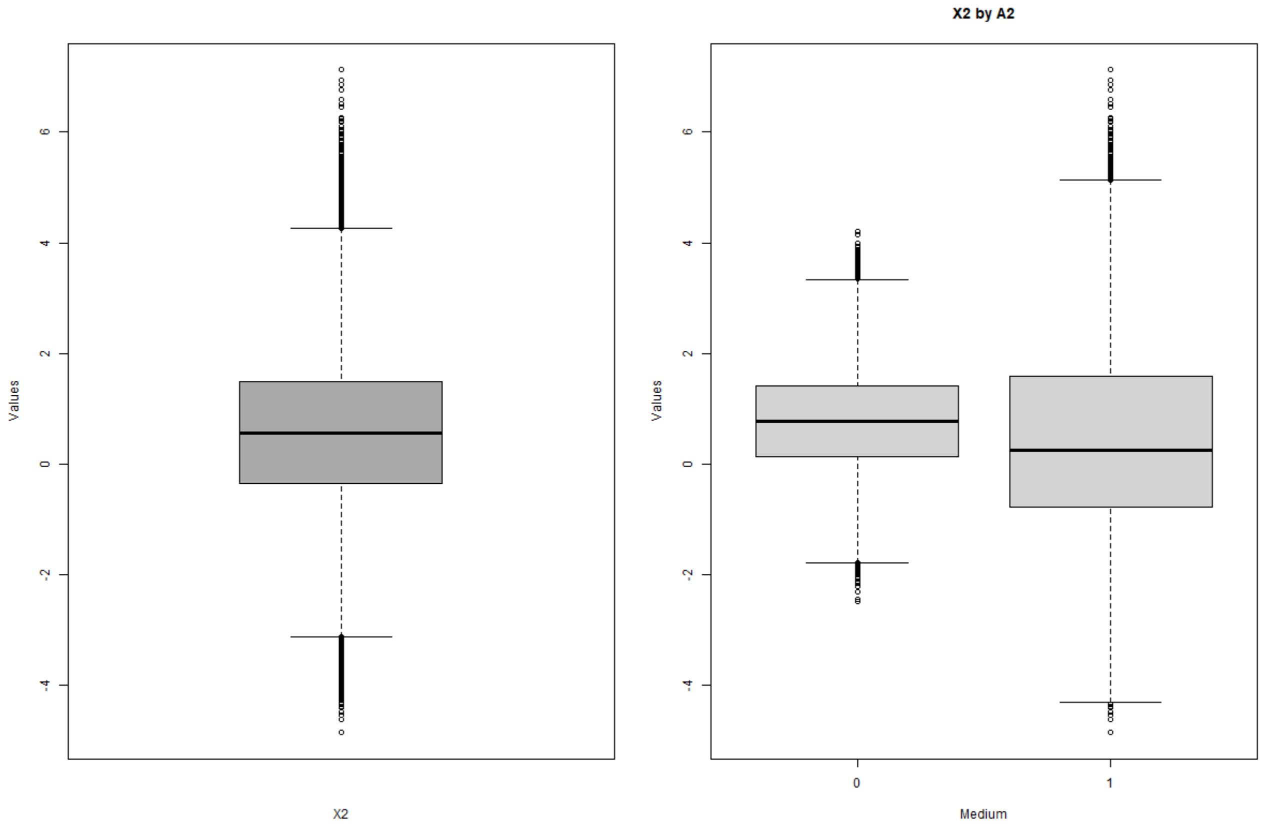

Figure 5: Study 4, Stage 2: Distribution of Covariates X_2 and Covariate X_2 by A_2, medium scenario

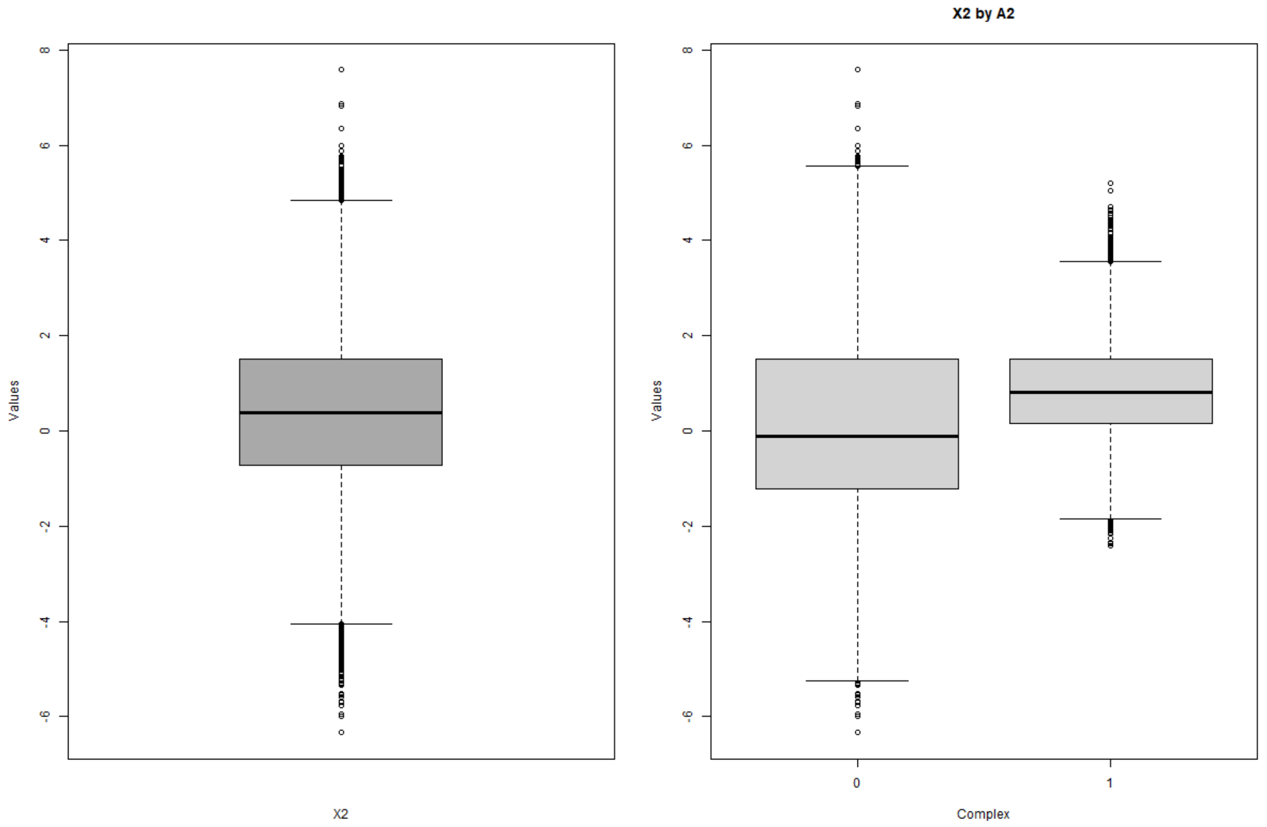

Figure 6: Study 4, Stage 2: Distribution of Covariates X_2 and Covariate X_2 by A_2, complex scenario

Performance metrics results for studies 1, 2, 3, and 4, with sample size n=1000

The results for the sample size of 1,000 are similar to those for the sample size of 300 described in the main material (Table 1, Table 2,Table 3, Table 4). As expected, most methods showed relatively low bias across all scenarios, except for SVM, which exhibited higher bias in certain cases. The standard deviations of the estimates were similar for most methods, except for RF and SVM, which showed increased standard deviations in some scenarios. In terms of performance, the RMSE ratio compared to logistic regression demonstrated that most methods, including the SuperLearner (SL), performed similarly to or even better than logistic regression. SL, in particular, performed the best among the machine learning methods, outperforming logistic regression in several scenarios. Due to a slight increase in bias and standard deviations, Bayes and RQL had a somewhat higher RMSE than logistic regression for some parameters. The results of Studies 1 to 4 are therefore consistent across both sample sizes, with Monte Carlo errors sufficiently small not to affect the interpretation of the results (Table 5).

Table 1: Results of Study 1: Simple outcome model and varying complexity of treatment model, $n =$ 1,000

	Simple scenario				Medium scenario				Complex scenario			
Methods	First time		Second time		First time		Second time		First time		Second time	
	ψ_{10}^*	ψ_{11}^*	ψ_{20}^*	ψ_{21}^*	ψ_{10}^*	ψ_{11}^*	ψ_{20}^*	ψ_{21}^*	ψ_{10}^*	ψ_{11}^*	ψ_{20}^*	ψ_{21}^*
	Bias				Bias				Bias			
Logit	-0.01	0.08	0.00	0.00	-0.06	0.08	-0.08	0.00	0.04	-0.07	0.08	-0.02
RF	0.00	0.02	-0.01	0.02	0.00	0.00	0.00	0.00	0.00	-0.01	0.01	-0.01
Bayes	0.00	-0.07	0.05	-0.08	-0.01	-0.02	0.00	-0.03	0.02	0.02	-0.01	0.04
Neural	0.00	0.05	-0.02	0.03	-0.02	0.01	-0.02	0.00	0.01	-0.01	0.03	-0.02
SVM	0.01	-0.05	0.05	-0.08	-0.02	-0.29	0.02	-0.16	0.02	0.14	0.09	-0.02
SL	0.00	0.03	-0.01	0.02	-0.02	0.01	-0.03	0.01	0.01	-0.01	0.03	-0.02
RQL	0.00	0.00	0.00	0.00	-0.01	0.01	-0.01	0.01	-0.01	-0.01	0.01	-0.02
	Standard deviation				Standard deviation				Standard deviation			
Logit	0.09	0.16	0.09	0.09	0.09	0.15	0.08	0.08	0.09	0.14	0.08	0.08
RF	0.11	0.19	0.10	0.10	0.10	0.18	0.09	0.09	0.10	0.17	0.09	0.08
Bayes	0.09	0.17	0.09	0.10	0.08	0.16	0.08	0.08	0.08	0.15	0.09	0.08
Neural	0.09	0.16	0.09	0.09	0.08	0.15	0.08	0.08	0.08	0.14	0.08	0.08
SVM	0.10	0.19	0.10	0.11	0.09	0.22	0.11	0.13	0.09	0.19	0.11	0.11
SL	0.09	0.16	0.09	0.09	0.08	0.15	0.08	0.08	0.08	0.15	0.08	0.08
RQL	0.09	0.17	0.09	0.09	0.09	0.15	0.08	0.08	0.09	0.14	0.09	0.08
	RMSE				RMSE				RMSE			
Logit	0.09	0.18	0.09	0.09	0.10	0.17	0.11	0.08	0.10	0.16	0.12	0.08
RF	0.11	0.19	0.10	0.10	0.10	0.18	0.09	0.09	0.10	0.17	0.09	0.08
Bayes	0.09	0.18	0.11	0.13	0.08	0.16	0.08	0.09	0.08	0.15	0.09	0.09
Neural	0.09	0.17	0.09	0.10	0.09	0.15	0.08	0.08	0.08	0.14	0.09	0.08
SVM	0.10	0.20	0.11	0.14	0.10	0.36	0.11	0.20	0.09	0.23	0.14	0.11
SL	0.09	0.16	0.09	0.09	0.09	0.15	0.09	0.08	0.08	0.15	0.09	0.08
RQL	0.09	0.17	0.09	0.09	0.09	0.15	0.08	0.08	0.09	0.15	0.09	0.08
	Ratio				Ratio				Ratio			
RF	1.14	1.08	1.09	1.10	0.98	1.06	0.79	1.12	1.05	1.10	0.76	1.08
Bayes	0.98	1.02	1.14	1.39	0.82	0.93	0.75	1.10	0.88	0.93	0.74	1.14
Neural	0.97	0.94	1.01	1.05	0.83	0.90	0.76	1.01	0.87	0.92	0.75	1.00
SVM	1.07	1.13	1.24	1.46	0.94	2.14	0.96	2.49	0.99	1.47	1.18	1.43
SL	0.97	0.92	1.00	1.01	0.85	0.91	0.78	1.04	0.87	0.92	0.75	1.02
RQL	0.95	0.94	0.93	0.99	0.88	0.89	0.75	1.04	0.93	0.92	0.73	1.04

Table 2: Results of Study 2: Simple treatment model and varying complexity of outcome model, $n =$ 1,000

	Simple scenario				Medium scenario				Complex scenario			
Methods	First time		Second time		First time		Second time		First time		Second time	
	ψ_{10}^*	ψ_{11}^*	ψ_{20}^*	ψ_{21}^*	ψ_{10}^*	ψ_{11}^*	ψ_{20}^*	ψ_{21}^*	ψ_{10}^*	ψ_{11}^*	ψ_{20}^*	ψ_{21}^*
	Bias				Bias				Bias			
Logit	0.00	0.08	-0.01	0.01	0.02	-0.49	0.01	-0.02	-0.01	0.33	0.00	0.00
RF	0.00	0.02	-0.01	0.02	0.00	-0.07	0.05	-0.08	0.00	0.03	-0.03	0.04
Bayes	0.01	-0.07	0.04	-0.08	-0.02	0.20	-0.13	0.21	0.02	-0.23	0.14	-0.24
Neural	0.00	0.04	-0.02	0.04	0.00	-0.16	0.06	-0.13	0.00	0.11	-0.05	0.10
SVM	0.01	-0.05	0.04	-0.07	-0.06	0.00	-0.07	0.06	0.06	-0.08	0.09	-0.12
SL	0.00	0.02	-0.01	0.02	0.00	-0.08	0.03	-0.06	0.00	0.05	-0.02	0.04
RQL	0.00	0.00	0.00	0.00	0.00	-0.01	0.00	0.00	0.00	0.00	0.00	0.00
	Standard deviation				Standard deviation				Standard deviation			
Logit	0.09	0.16	0.09	0.09	0.13	0.25	0.11	0.12	0.10	0.19	0.10	0.11
RF	0.11	0.19	0.09	0.10	0.15	0.31	0.11	0.12	0.12	0.22	0.11	0.11
Bayes	0.09	0.17	0.09	0.09	0.13	0.28	0.10	0.13	0.10	0.20	0.10	0.12
Neural	0.09	0.16	0.09	0.09	0.12	0.26	0.10	0.12	0.09	0.19	0.10	0.11
SVM	0.10	0.20	0.10	0.11	0.15	0.49	0.14	0.19	0.12	0.37	0.14	0.17
SL	0.09	0.16	0.09	0.09	0.13	0.26	0.10	0.12	0.10	0.19	0.10	0.11
RQL	0.09	0.17	0.09	0.09	0.13	0.25	0.09	0.10	0.10	0.18	0.09	0.10
	RMSE				RMSE				RMSE			
Logit	0.09	0.18	0.09	0.09	0.14	0.55	0.11	0.12	0.10	0.38	0.10	0.11
RF	0.11	0.19	0.10	0.10	0.15	0.32	0.11	0.14	0.12	0.23	0.11	0.12
Bayes	0.09	0.18	0.10	0.12	0.13	0.34	0.16	0.25	0.11	0.31	0.18	0.27
Neural	0.09	0.17	0.09	0.09	0.12	0.30	0.12	0.18	0.09	0.22	0.11	0.15
SVM	0.10	0.20	0.11	0.13	0.16	0.49	0.16	0.20	0.13	0.38	0.17	0.21
SL	0.09	0.17	0.09	0.09	0.13	0.27	0.11	0.13	0.10	0.19	0.10	0.12
RQL	0.09	0.17	0.09	0.09	0.13	0.25	0.09	0.10	0.10	0.18	0.09	0.10
	Ratio				Ratio				Ratio			
RF	1.14	1.06	1.09	1.13	1.08	0.58	1.09	1.18	1.12	0.60	1.06	1.12
Bayes	0.98	1.02	1.15	1.39	0.98	0.62	1.55	2.04	1.04	0.81	1.72	2.47
Neural	0.97	0.94	1.00	1.08	0.91	0.55	1.13	1.45	0.92	0.58	1.06	1.35
SVM	1.06	1.14	1.25	1.48	1.19	0.88	1.51	1.67	1.26	1.00	1.61	1.93
SL	0.97	0.93	1.01	1.04	0.93	0.49	1.01	1.10	0.94	0.51	1.00	1.08
RQL	0.97	0.94	0.99	1.06	0.97	0.44	0.86	0.80	0.95	0.47	0.89	0.87

Table 3: Results of Study 3: Varying complexity of treatment and outcome models, n = 1,000

	Simple scenario				Medium scenario				Complex scenario			
Methods	First time		Second time		First time		Second time		First time		Second time	
	ψ^*_{10}	ψ^*_{11}	ψ^*_{20}	ψ^*_{21}	ψ^*_{10}	ψ^*_{11}	ψ^*_{20}	ψ^*_{21}	ψ^*_{10}	ψ^*_{11}	ψ^*_{20}	ψ^*_{21}
	Bias				Bias				Bias			
Logit	0.00	0.01	0.00	0.00	0.18	-0.06	0.19	0.04	0.14	0.00	0.22	-0.04
RF	0.00	0.02	-0.01	0.02	0.01	-0.01	0.02	-0.02	0.01	-0.01	0.02	-0.01
Bayes	0.00	-0.07	0.05	-0.08	0.05	0.05	-0.01	0.08	0.07	0.05	-0.05	0.13
Neural	0.00	0.05	-0.02	0.03	0.09	-0.07	0.08	0.00	0.06	-0.06	0.09	-0.05
SVM	0.01	-0.05	0.05	-0.08	0.03	1.36	-0.08	0.44	0.07	0.56	0.24	-0.05
SL	0.00	0.03	-0.01	0.02	0.09	-0.06	0.08	-0.02	0.05	-0.04	0.07	-0.04
RQL	0.00	0.00	0.00	0.00	0.01	-0.01	0.01	0.00	0.00	-0.01	0.01	-0.01
	standard deviation				standard deviation				Standard deviation			
Logit	0.09	0.16	0.09	0.09	0.13	0.26	0.10	0.11	0.09	0.17	0.10	0.08
RF	0.11	0.19	0.10	0.10	0.15	0.29	0.10	0.10	0.11	0.19	0.09	0.09
Bayes	0.09	0.17	0.09	0.10	0.13	0.26	0.10	0.11	0.09	0.17	0.10	0.09
Neural	0.09	0.16	0.09	0.09	0.12	0.26	0.09	0.10	0.09	0.16	0.09	0.08
SVM	0.10	0.19	0.10	0.11	0.15	0.43	0.19	0.26	0.10	0.29	0.18	0.21
SL	0.09	0.16	0.09	0.09	0.12	0.26	0.09	0.10	0.09	0.16	0.09	0.09
RQL	0.09	0.17	0.09	0.09	0.12	0.25	0.09	0.09	0.09	0.15	0.09	0.08
	RMSE				RMSE				RMSE			
Logit	0.09	0.16	0.09	0.09	0.22	0.26	0.21	0.11	0.17	0.17	0.24	0.09
RF	0.11	0.19	0.10	0.10	0.15	0.29	0.10	0.11	0.11	0.19	0.10	0.09
Bayes	0.09	0.18	0.11	0.13	0.14	0.26	0.10	0.14	0.11	0.17	0.11	0.16
Neural	0.09	0.17	0.09	0.10	0.15	0.27	0.12	0.10	0.11	0.17	0.13	0.10
SVM	0.10	0.20	0.11	0.14	0.15	1.43	0.21	0.51	0.13	0.62	0.30	0.21
SL	0.09	0.16	0.09	0.09	0.15	0.27	0.12	0.11	0.10	0.17	0.11	0.10
RQL	0.09	0.17	0.09	0.09	0.12	0.25	0.09	0.09	0.09	0.16	0.09	0.08
	Ratio				Ratio				Ratio			
RF	1.17	1.17	1.09	1.10	0.66	1.11	0.47	0.95	0.64	1.13	0.40	0.97
Bayes	1.01	1.11	1.14	1.39	0.61	0.99	0.45	1.25	0.66	1.03	0.44	1.71
Neural	0.99	1.02	1.01	1.05	0.68	1.00	0.56	0.90	0.64	1.04	0.53	1.04
SVM	1.10	1.23	1.24	1.46	0.68	5.38	0.96	4.54	0.76	3.72	1.22	2.28
SL	1.00	1.00	1.00	1.01	0.68	1.01	0.57	0.95	0.61	0.99	0.46	1.04
RQL	0.98	1.02	0.93	0.99	0.56	0.95	0.41	0.77	0.55	0.92	0.36	0.87

Table 4: Results of Study 4 : Varying complexity of treatment and outcome models, and practical positivity violations, n = 1,000

	Simple scenario				Medium scenario				Complex scenario			
Methods	First time		Second time		First time		Second time		First time		Second time	
	ψ_{10}^*	ψ_{11}^*	ψ_{20}^*	ψ_{21}^*	ψ_{10}^*	ψ_{11}^*	ψ_{20}^*	ψ_{21}^*	ψ_{10}^*	ψ_{11}^*	ψ_{20}^*	ψ_{21}^*
	Bias				Bias				Bias			
Logit	0.00	0.01	-0.01	0.01	1.37	-0.19	1.62	0.32	0.87	0.09	1.21	-0.18
RF	0.00	0.04	-0.04	0.06	0.02	0.07	0.08	0.02	0.02	0.03	0.03	0.01
Bayes	0.01	-0.06	0.08	-0.14	0.20	-0.02	-0.01	0.02	0.18	-0.08	-0.15	0.09
Neural	0.00	0.13	-0.06	0.13	0.65	-0.32	0.43	0.13	0.41	-0.28	0.50	-0.22
SL	0.00	0.02	-0.01	0.02	0.29	0.11	0.16	0.27	0.22	0.04	0.15	0.00
RQL	0.00	-0.01	0.00	0.01	0.04	-0.14	0.06	0.03	0.01	0.12	0.07	-0.04
	Standard deviation				Standard deviation				Standard deviation			
Logit	0.11	0.14	0.09	0.08	0.27	0.45	0.20	0.19	0.17	0.25	0.16	0.12
RF	0.13	0.16	0.09	0.08	0.29	0.54	0.15	0.16	0.17	0.27	0.13	0.12
Bayes	0.11	0.14	0.09	0.09	0.24	0.45	0.14	0.17	0.14	0.22	0.13	0.13
Neural	0.11	0.13	0.09	0.07	0.24	0.44	0.15	0.16	0.14	0.23	0.13	0.12
SL	0.11	0.14	0.09	0.08	0.26	0.50	0.20	0.33	0.16	0.26	0.15	0.16
RQL	0.11	0.14	0.10	0.09	0.22	0.54	0.11	0.10	0.15	0.22	0.10	0.07
	RMSE				RMSE				RMSE			
Logit	0.11	0.14	0.09	0.08	1.40	0.49	1.63	0.37	0.89	0.26	1.22	0.21
RF	0.13	0.16	0.10	0.10	0.29	0.54	0.17	0.16	0.18	0.27	0.14	0.12
Bayes	0.11	0.15	0.12	0.16	0.32	0.45	0.14	0.17	0.22	0.23	0.20	0.16
Neural	0.11	0.18	0.11	0.15	0.69	0.54	0.46	0.21	0.43	0.36	0.51	0.25
SL	0.11	0.14	0.09	0.08	0.39	0.51	0.25	0.42	0.28	0.26	0.21	0.16
RQL	0.11	0.14	0.10	0.09	0.23	0.56	0.12	0.11	0.15	0.25	0.12	0.08
	Ratio				Ratio				Ratio			
RF	1.20	1.21	1.11	1.29	0.21	1.11	0.10	0.44	0.20	1.03	0.11	0.57
Bayes	1.03	1.13	1.32	2.03	0.23	0.92	0.09	0.46	0.25	0.89	0.17	0.76
Neural	1.00	1.34	1.19	1.90	0.49	1.11	0.28	0.56	0.48	1.39	0.42	1.18
SL	1.01	1.00	0.99	1.02	0.28	1.04	0.15	1.14	0.31	1.01	0.17	0.75
RQL	1.06	1.02	1.07	1.08	0.16	1.14	0.07	0.29	0.16	0.95	0.09	0.38

Table 5: Monte Carlo standard error of Studies 1-4

Methods	Simple scenario				Medium scenario				Complex scenario			
	Bias		Standard deviation		Bias		Standard deviation		Bias		Standard deviation	
n	300	1000	300	1000	300	1000	300	1000	300	1000	300	1000
Study 1: Simple outcome model and varying complexity of treatment model												
Logit	0.01	0.00	0.01	0.00	0.01	0.00	0.01	0.00	0.01	0.00	0.01	0.00
RF	0.01	0.01	0.01	0.00	0.01	0.01	0.01	0.00	0.01	0.01	0.01	0.00
Bayes	0.01	0.01	0.01	0.00	0.01	0.00	0.01	0.00	0.01	0.00	0.01	0.00
Neural	0.01	0.01	0.01	0.00	0.01	0.00	0.01	0.00	0.01	0.00	0.01	0.00
SVM	0.01	0.01	0.01	0.00	0.01	0.01	0.01	0.00	0.01	0.01	0.01	0.00
SL	0.01	0.01	0.01	0.00	0.01	0.00	0.01	0.00	0.01	0.00	0.01	0.00
RQL	0.01	0.01	0.01	0.00	0.01	0.00	0.01	0.00	0.01	0.00	0.01	0.00
Study 2: Simple treatment model and varying complexity of outcome model												
Logit	0.01	0.01	0.01	0.00	0.01	0.01	0.01	0.01	0.01	0.01	0.01	0.00
RF	0.01	0.01	0.01	0.00	0.02	0.01	0.01	0.01	0.01	0.01	0.01	0.01
Bayes	0.01	0.01	0.01	0.00	0.02	0.01	0.01	0.01	0.01	0.01	0.01	0.00
Neural	0.01	0.01	0.01	0.00	0.01	0.01	0.01	0.01	0.01	0.01	0.01	0.00
SVM	0.01	0.01	0.01	0.00	0.02	0.02	0.02	0.01	0.02	0.01	0.01	0.01
SL	0.01	0.01	0.01	0.00	0.02	0.01	0.01	0.01	0.01	0.01	0.01	0.00
RQL	0.01	0.01	0.01	0.00	0.02	0.01	0.01	0.01	0.01	0.01	0.01	0.00
Study 3: Varying complexity of treatment and outcome models												
Logit	0.01	0.01	0.01	0.00	0.01	0.01	0.01	0.01	0.01	0.01	0.01	0.00
RF	0.01	0.01	0.01	0.00	0.02	0.01	0.01	0.01	0.01	0.01	0.01	0.00
Bayes	0.01	0.01	0.01	0.00	0.01	0.01	0.01	0.01	0.01	0.01	0.01	0.00
Neural	0.01	0.01	0.01	0.00	0.01	0.01	0.01	0.01	0.01	0.01	0.01	0.00
SVM	0.01	0.01	0.01	0.00	0.02	0.01	0.02	0.01	0.01	0.01	0.01	0.01
SL	0.01	0.01	0.01	0.00	0.01	0.01	0.01	0.01	0.01	0.01	0.01	0.00
RQL	0.01	0.01	0.01	0.00	0.01	0.01	0.01	0.01	0.01	0.00	0.01	0.00
Study 4 : Varying complexity of treatment and outcome models, and practical positivity violations												
Logit	0.01	0.00	0.01	0.00	0.03	0.01	0.02	0.01	0.01	0.01	0.01	0.01
RF	0.01	0.01	0.01	0.00	0.03	0.02	0.02	0.01	0.01	0.01	0.01	0.01
Bayes	0.01	0.00	0.01	0.00	0.03	0.01	0.02	0.01	0.01	0.01	0.01	0.00
Neural	0.01	0.00	0.00	0.00	0.03	0.01	0.02	0.01	0.01	0.01	0.01	0.01
SL	0.01	0.00	0.01	0.00	0.03	0.02	0.02	0.01	0.01	0.01	0.01	0.01
RQL	0.01	0.00	0.01	0.00	0.04	0.02	0.03	0.01	0.01	0.01	0.01	0.00

Appendix 2 – Additional simulation study 5

Based on Study 4, Study 5 includes 10 covariates at each time point. The objective is to evaluate our approach in a higher dimensional setting. The history at the first time point was $H_1 = (X_{11}, X_{12}, X_{13}, X_{14}, X_{15}, X_{16}, X_{17}, X_{18}, X_{19}, X_{110})$, and $H_2 = (X_{11}, X_{12}, X_{13}, X_{14}, X_{15}, X_{16}, X_{17}, X_{18}, X_{19}, X_{110}, A_1, X_{21}, X_{22}, X_{23}, X_{24}, X_{25}, X_{26}, X_{27}, X_{28}, X_{29}, X_{210})$ at the second time point, for all scenarios. The data generating equation for all scenarios were:

- First time point: $X_{11} \sim \text{Bernoulli}(p = 0.5)$, $X_{12} \sim \text{Bernoulli}(p = 0.05{+}0.5X_{11})$, $X_{13} \sim N(0, 0.5)$, $X_{14} \sim \text{Bernoulli}(p = \text{expit}(-0.4 + X_{12}))$, $X_{15} \sim \text{Uniform}(0, 1)$, $X_{16} \sim \text{Discrete uniform}\{1, 2, 3\}$, $X_{17} \sim \text{Discrete uniform}\{1, 2, 3\}$, $X_{18} \sim X_{11}X_{14} + N(0, 0.5)$, $X_{19} \sim \log(X_{15} + 1) + \text{Uniform}(0, 1)$, $X_{110} \sim -X_{13} + X_{12}X_{15} + \text{Uniform}(0, 1)$.

- Second time point: $X_{21} \sim X_{11} + A_1 + N(0, 0.1)$, $X_{22} \sim \text{Bernoulli}(p = \text{expit}(-0.1 + 0.3X_{11} + A_1))$, $X_{23} \sim X_{13} + A_1 + N(0, 0.2)$, $X_{24} \sim \text{Bernoulli}(p = \text{expit}(-0.4 + X_{22} + A_1))$, $X_{25} \sim X_{15} + A_1 + \text{Uniform}(-1, 1)$, $X_{26} \sim X_{16} + A_1 + \text{Discrete uniform}\{0, 1, 2\}$, $X_{27} \sim \text{Discrete uniform}\{1, 2, 3\}$, $X_{28} \sim |X_{21} - X_{24}| + \sin(X_{23} + A_1) + \text{Uniform}(0, 1)$, $X_{29} \sim \cos(X_{25} + X_{15} + A_1) + \text{Uniform}(0, 1)$, $X_{210} \sim 0.5X_{23} + 0.5X_{21} + A_1 + \text{Uniform}(0, 1)$.

In addition, for all scenarios in this study, ψ_1 =(-3,1,-0.2,0.3,1,0.5,1,-0.2,0.3,1,0.5), ψ_2 =(-0.5,1,-0.2,0.3,1,0.5,1,-0.2,0.3,1,0.5) and L_t=$(1, X_{t1}, X_{t2}, X_{t3}, X_{t4}, X_{t5}, X_{t6}, X_{t7}, X_{t8}, X_{t9}, X_{t10})$,

$t = 1, 2$. At the second time point, the true values of the causal parameters are $(\psi^*_{20}, \psi^*_{21}, \psi^*_{22}, \psi^*_{23}, \psi^*_{24}, \psi^*_{25}, \psi^*_{26}, \psi^*_{27}, \psi^*_{28}, \psi^*_{29}, \psi^*_{210}) = (-0.5, 1, -0.2, 0.3, 1, 0.5, 1, -0.2, 0.3, 1, 0.5)$. The true value of the effect at the first time point was determined using a Monte Carlo simulation of counterfactual outcomes in these scenarios. In the **simple scenario**, the true values of the parameters were $(\psi^*_{10}, \psi^*_{11}, \psi^*_{12}, \psi^*_{13}, \psi^*_{14}, \psi^*_{15}, \psi^*_{16}, \psi^*_{17}, \psi^*_{18}, \psi^*_{19}, \psi^*_{110}) = (\psi_{10}, \psi_{11}, \psi_{12}, \psi_{13}, \psi_{14}, \psi_{15}, \psi_{16}, \psi_{17}, \psi_{18}, \psi_{19}, \psi_{110}) \approx$ (2.41, 1.95, -0.2, -0.93, 1.0, 1.59, 1.0, -0.2, 0.3, 0.96, 0.5); in the **medium scenario**, the true values were $(\psi^*_{10}, \psi^*_{11}, \psi^*_{12}, \psi^*_{13}, \psi^*_{14}, \psi^*_{15}, \psi^*_{16}, \psi^*_{17}, \psi^*_{18}, \psi^*_{19}, \psi^*_{110}) \approx (-10.61, -1.42, -0.19, 3.09, 1.01, 1.35, 0.01, -0.19, 0.3, 1.01, 0.51)$ and for the **complex scenario**, the true values were $(\psi^*_{10}, \psi^*_{11}, \psi^*_{12}, \psi^*_{13}, \psi^*_{14}, \psi^*_{15}, \psi^*_{16}, \psi^*_{17}, \psi^*_{18}, \psi^*_{19}, \psi^*_{110}) \approx (-4.35, 1.21, -0.2, -1.9, 1.0, 0.98, -1.0, -0.2, 0.3, 0.99, 0.5)$.

The scenarios are defined as follows: **Simple scenario:** $A_1 \sim \text{Bernoulli}(p = \text{expit}(-4 - 1.5X_{11} + 5.5X_{13} - 5.5X_{14} - 1.5X_{15} + 2.5X_{16} + 2.5X_{17} + X_{18} - 0.5X_{19} + X_{110}))$, $A_2 \sim \text{Bernoulli}(p = \text{expit}(15 - X_{21} - X_{23} - 3.5X_{24} + X_{25} - 2.5X_{26} - X_{27} - X_{28} - 0.5X_{29} - X_{210}))$, and $Y \sim \text{N}(X_{11} + X_{12} + X_{13} + X_{14} + X_{15} + X_{16} + X_{17} + X_{18} + X_{19} + X_{110} + X_{21} + X_{22} + X_{23} + X_{24} + X_{25} + X_{26} + X_{27} + X_{28} + X_{29} + X_{210} - \mu_1 - \mu_2, 1)$;

Medium scenario: $A_1 \sim \text{Bernoulli}(p = \text{expit}(5 - 1.5X_{11} + 0.5X_{12} - 0.5X_{14} + 0.5X_{13} - 1.5X_{15} - X_{16} - X_{17} + X_{18} - 0.5X_{19} + X_{110} + 0.5X^2_{13} + X^2_{15} - 0.5X^2_{18} + X^2_{19} - 0.5X^2_{110}))$, $A_2 \sim \text{Bernoulli}(p = \text{expit}(5 - X_{21} - X_{23} - X_{24} + X_{25} - 0.5X_{26} - X_{27} - X_{28} - 0.5X_{29} - X_{210} + 0.5X^2_{21} + 0.5X^2_{23} + 0.5X^2_{25} - 0.1X^2_{26} - 0.5X^2_{28} + X^2_{29} - 0.5X^2_{210}))$, and $Y \sim \text{N}(X_{11} + X_{13} - X_{14} + X_{15} - X_{16} + X_{17} - X_{18} - 0.5X_{19} - X_{110} + X^2_{13} + 0.5X^2_{15} - 0.5X^2_{16} + 0.5X^2_{17} - 0.5X^2_{18} + 0.25X^2_{19} - 0.25X^2_{110} - X_{21} - X_{23} - 1.5X_{24} + X_{25} - X_{26} + X_{27} - X_{28} - 0.5X_{29} - X_{210} + 0.5X^2_{21} + 0.25X^2_{23} + 0.25X^2_{25} - 0.5X^2_{26} + 0.5X^2_{27} - 0.5X^2_{28} + 0.5X^2_{29} - 0.25X^2_{210} - \mu_1 - \mu_2, 1)$;

Complex scenario: $A_1 \sim \text{Bernoulli}(p = \text{expit}(4 - 1.5X_{11} + 0.5X_{12} + \sin(X_{13}) - X_{14} + \cos(X_{15}) - X_{16} + \sin(\cos(X^2_{13})) + |\sin(X^2_{15})| - \cos(X^2_{17}) - \log(X^2_{18}) + X^2_{19} - \exp(\tan(X^2_{110}))))$, $A_2 \sim \text{Bernoulli}(p = \text{expit}(-0.5X_{21} + \sin(X_{23}) - X_{24} + \cos(X_{25}) + X^2_{21} + \sin(\cos(X^2_{23})) + |\sin(X^2_{25})| - 0.1X^2_{26} - \cos(X^2_{27}) - \log(X^2_{28}) + X^2_{29} - \exp(\tan(X^2_{210}))))$, and $Y \sim \text{N}(1.5 + 1.5X_{11} + \sin(X_{13}) - 1.5X_{14} + \cos(X_{15}) + 1.5\sin(\cos(X^2_{13})) + |\sin(X^2_{15})| - 0.1X^2_{16} + \cos(X^2_{17}) - 0.5\log(X^2_{18} + 1) + 0.5X^2_{19} - \cos(X^2_{110}) - X_{21} + \sin(X_{23}) - X_{24} + \cos(X_{25}) + 0.5X^2_{21} + 1.5\sin(\cos(X^2_{23})) - |\sin(X^2_{25})| - 0.1X^2_{26} + \cos(X^2_{27}) - 0.25\log(X^2_{28} + 1) + 0.5X^2_{29} - 1.5\cos(X^2_{210}) - \mu_1 - \mu_2, 1)$.

Distribution of treatment probabilities and covariates for study 5

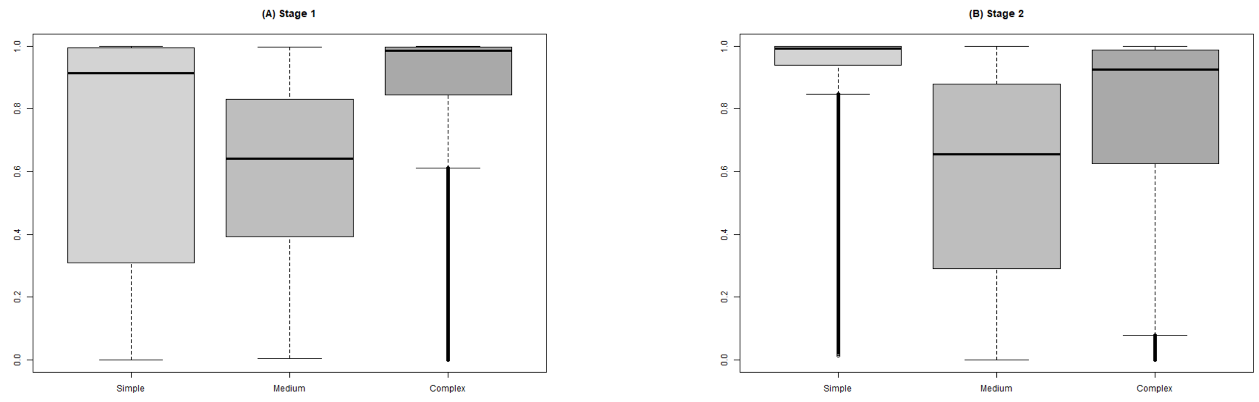

Figure 7: Distribution of the treatment probabilities: Study 5, (A) Stage 1, (B) Stage 2

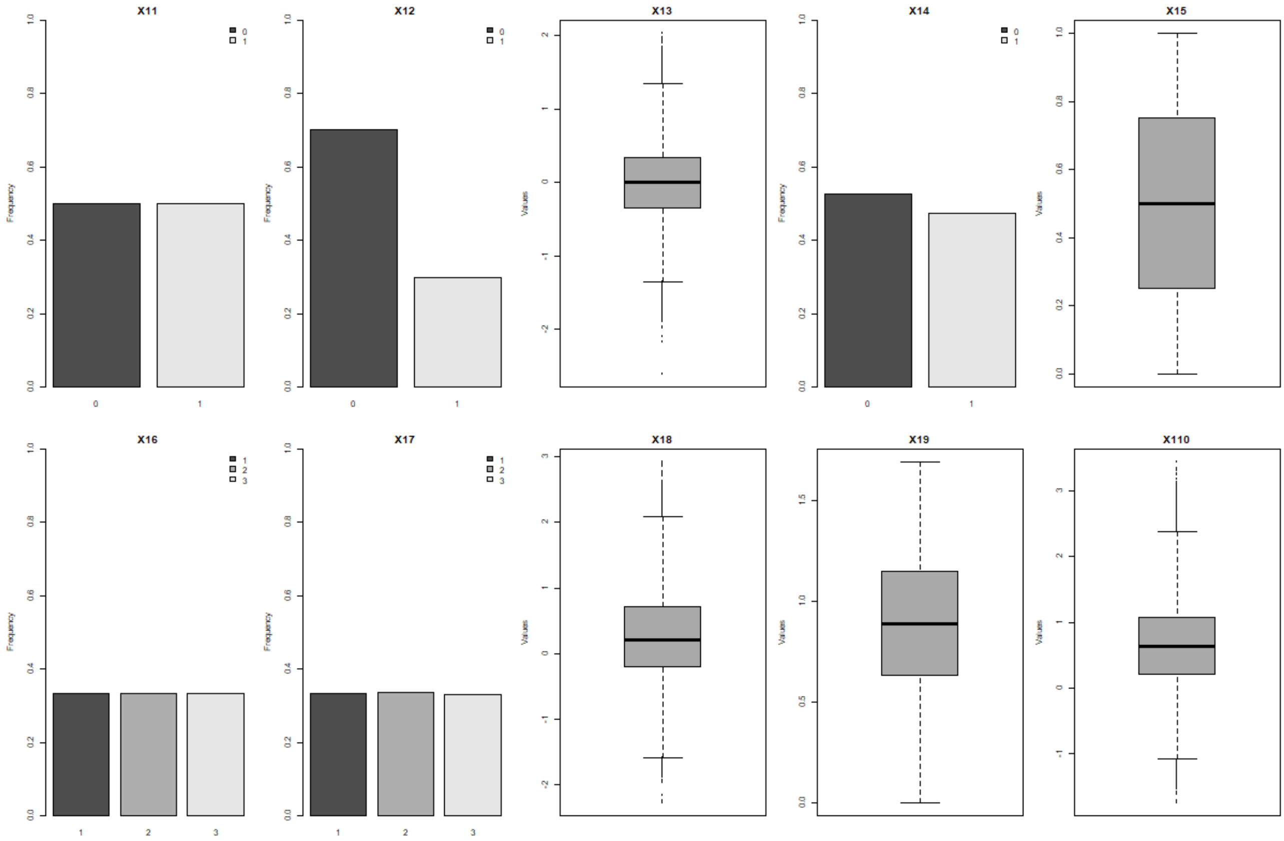

Figure 8: Distribution of Covariates: Study 5, Stage 1, for all scenarios

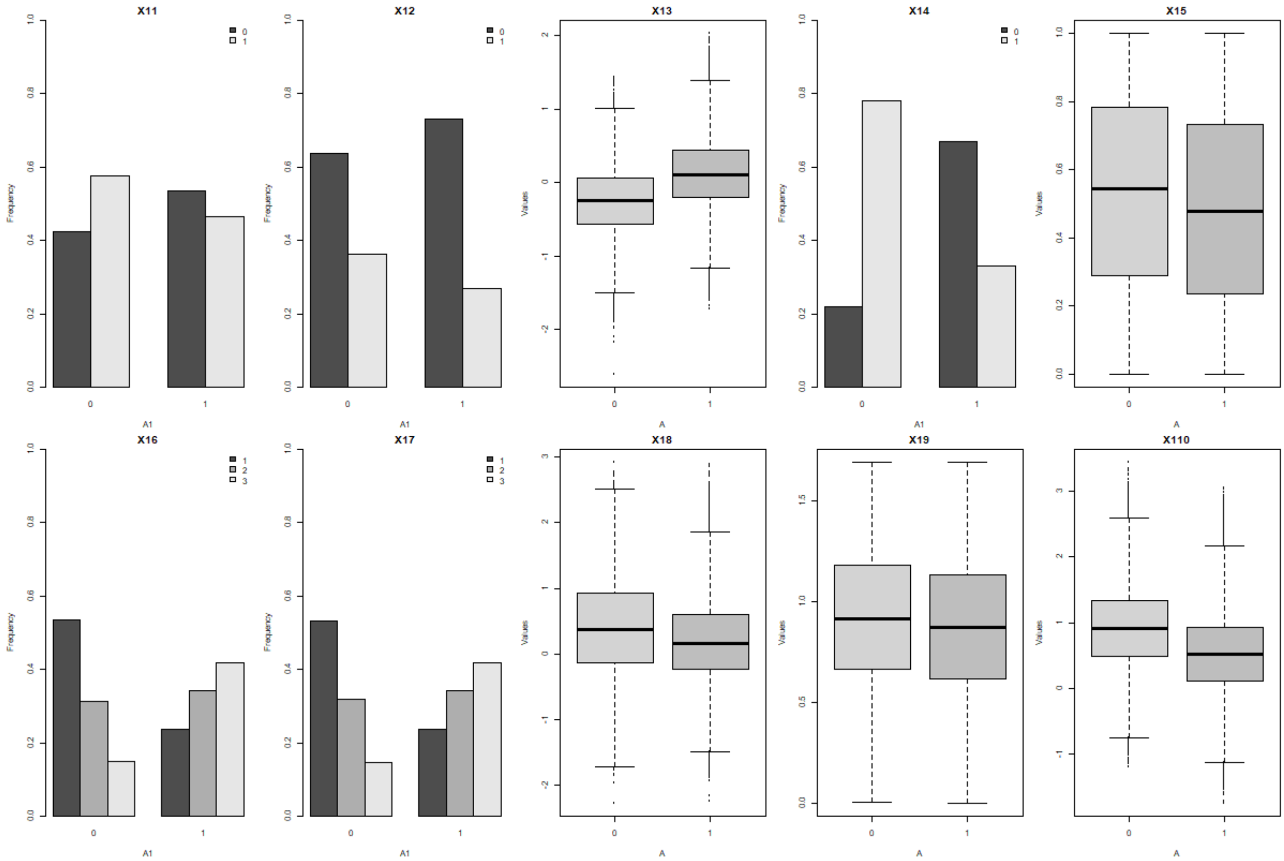

Figure 9: Study 5, Stage 1, Distribution of Covariates by A1, scenario simple

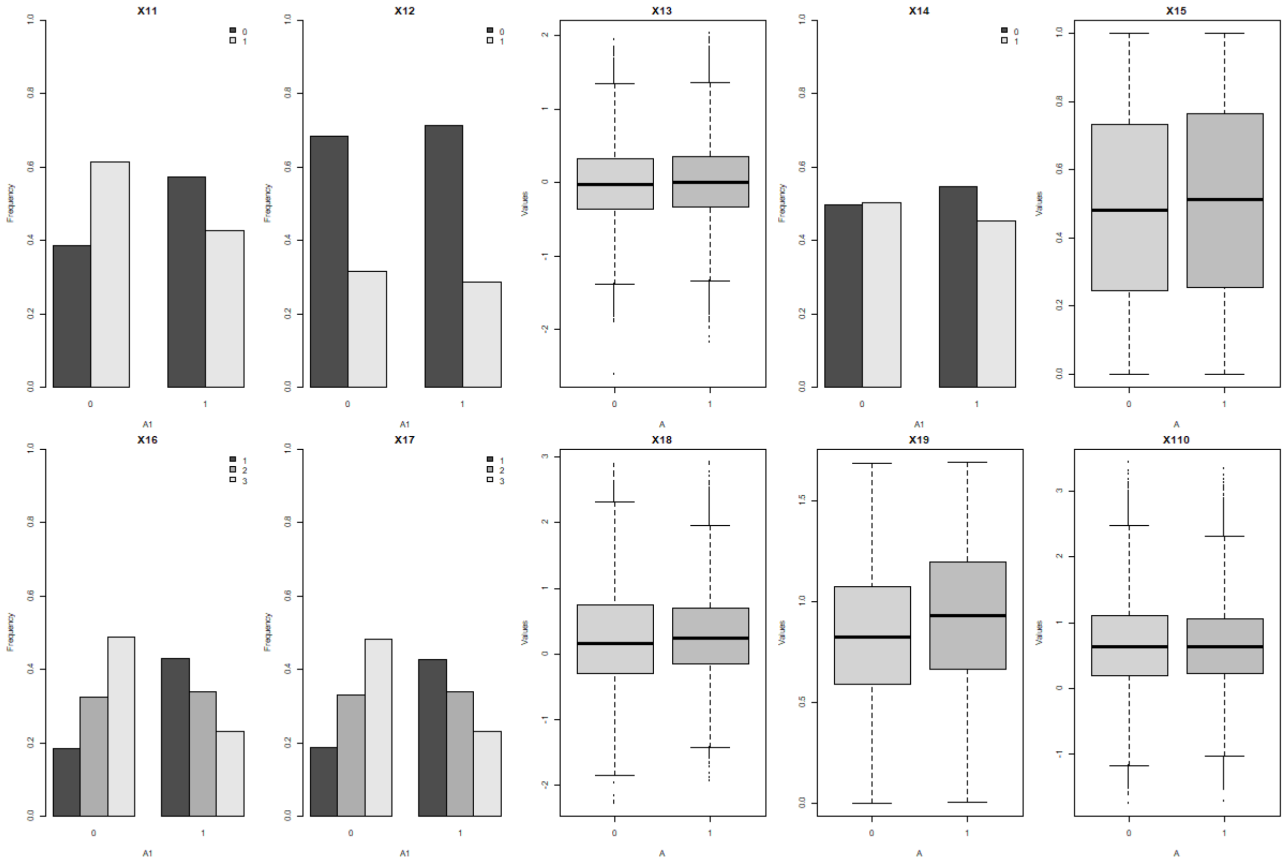

Figure 10: Study 5, Stage 1, Distribution of Covariates by A1, scenario medium

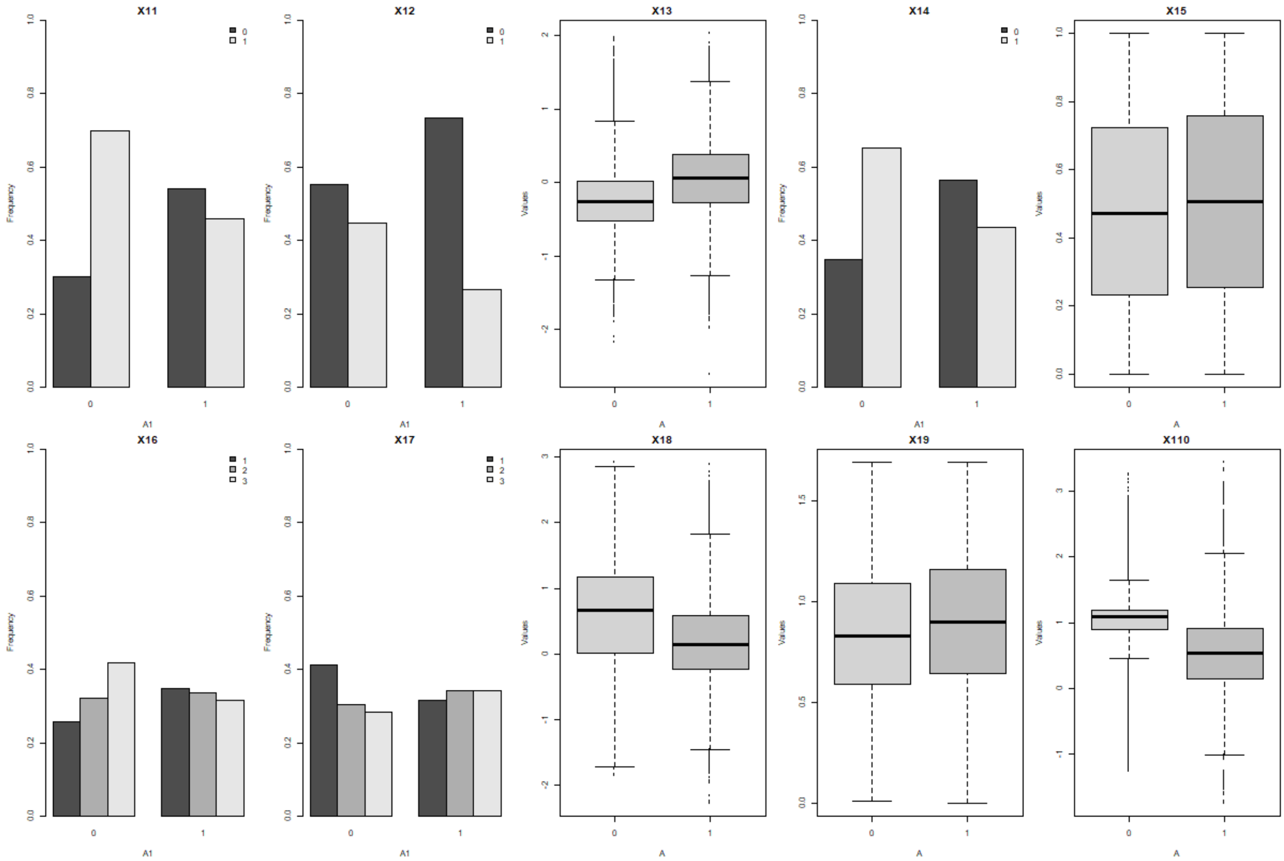

Figure 11: Study 5, Stage 1, Distribution of Covariates by A1, scenario complex

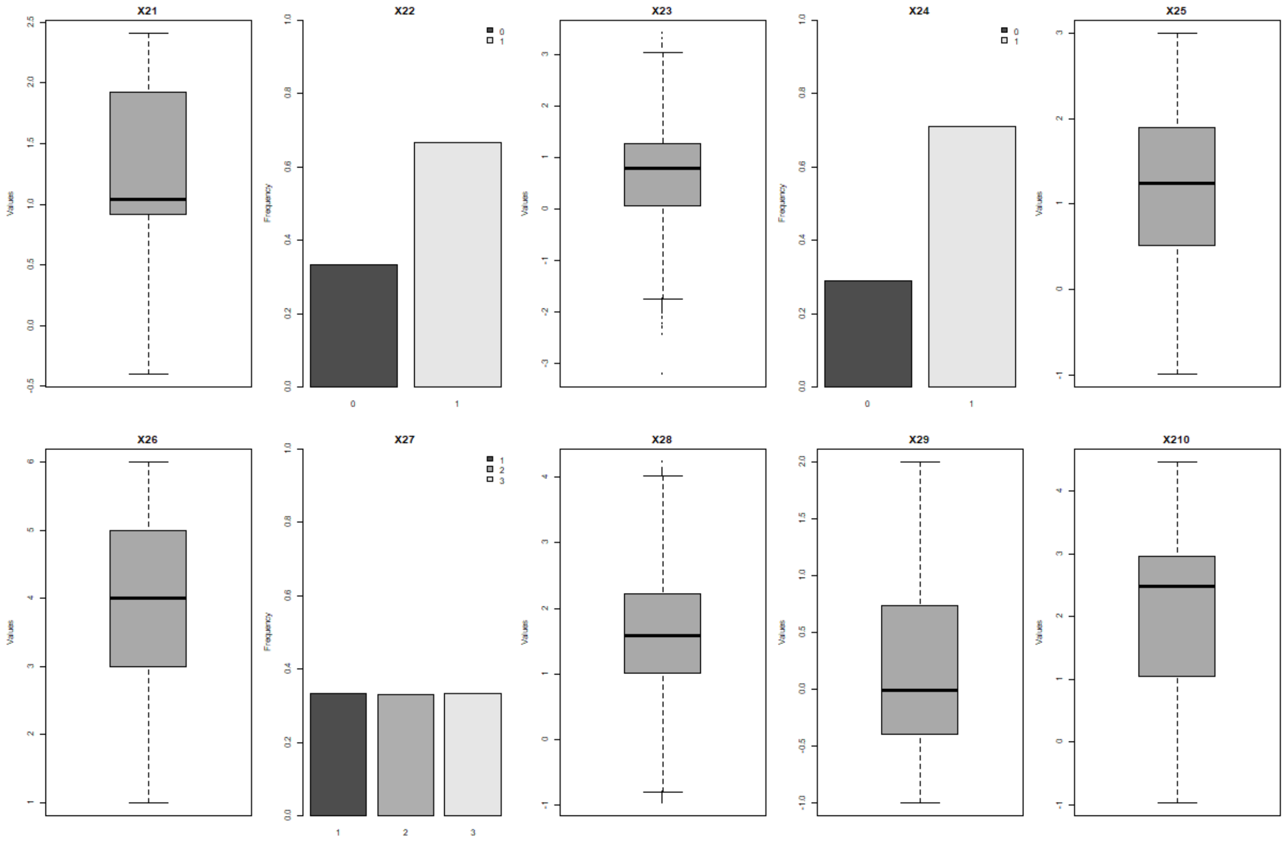

Figure 12: Distribution of Covariates: Study 5, Stage 2, scenario simple

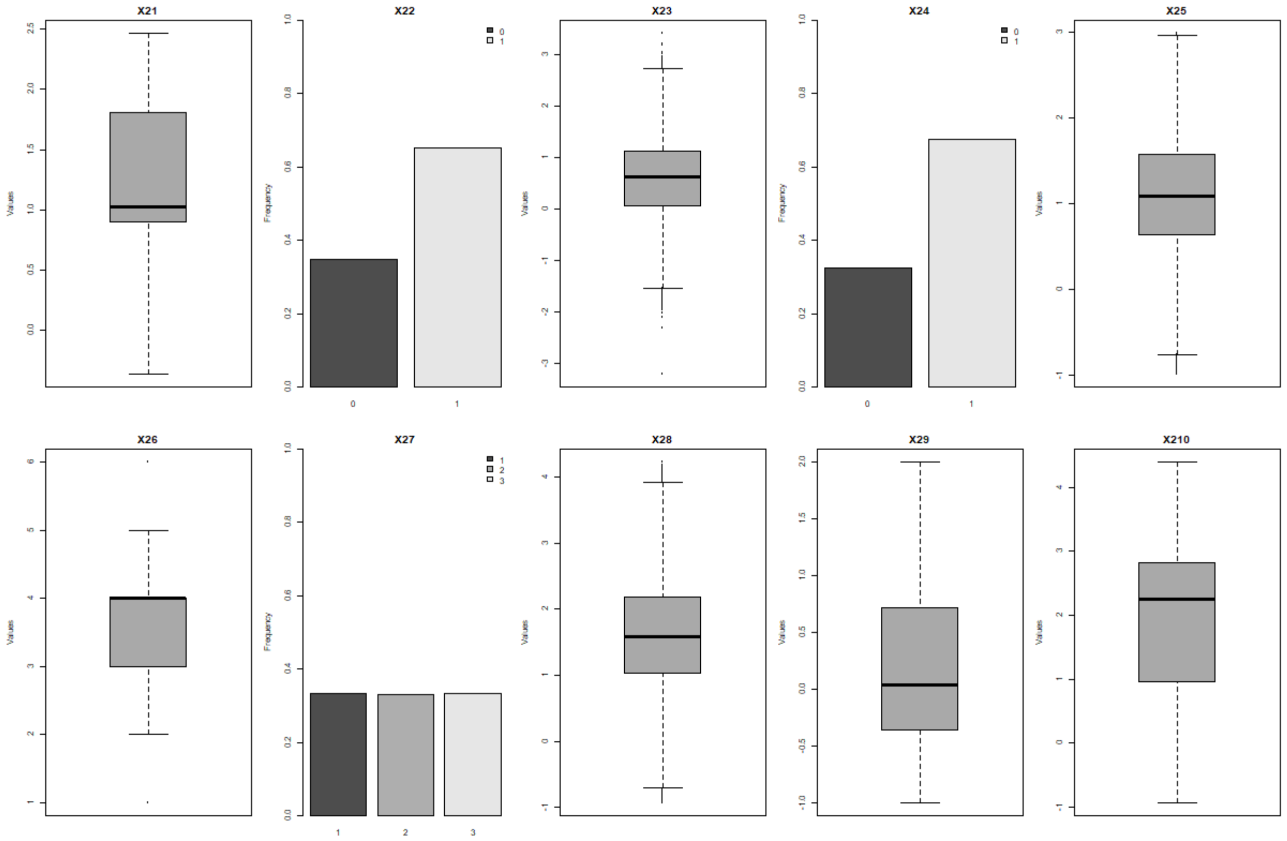

Figure 13: Distribution of Covariates: Study 5, Stage 2, scenario medium

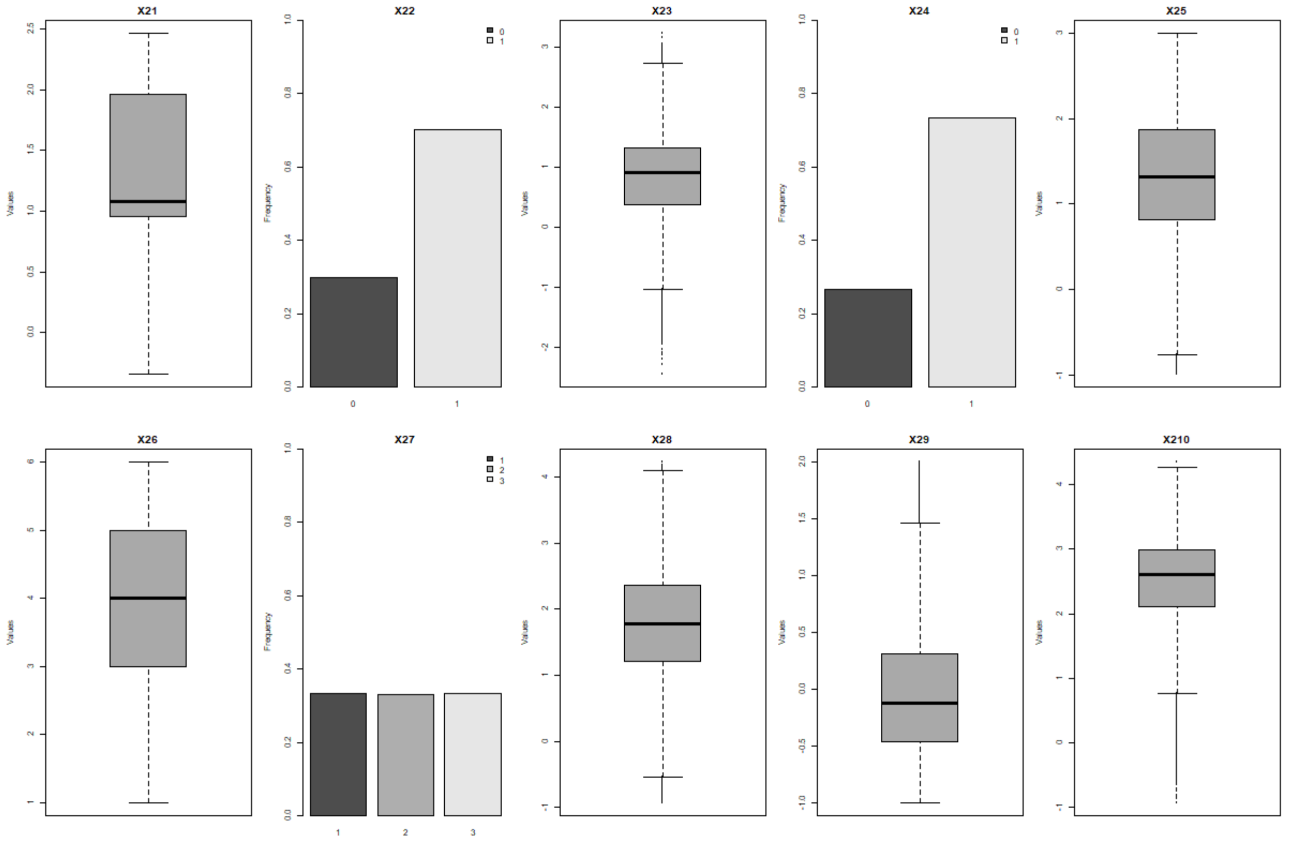

Figure 14: Distribution of Covariates: Study 5, Stage 2, scenario complex

Performance metrics results for study 5

The results for Study 5 were similar for $n = 300$ and $n = 1000$. The results of Study 5 for $n = 300$ are summarized in Tables 6, 7, and 8.

In the simple scenario, most methods exhibited relatively low bias across parameters, though some variations were observed. The Logit showed moderate biases for certain parameters, but no extreme deviations were noted. RF and SL demonstrated stable performance with minimal bias, while Bayes and Neural showed slight variations across parameters. The RQL method, however, exhibited higher biases, particularly for parameters like ψ_{20}^* and ψ_{21}^*.

In the medium scenario, biases increased across many methods, with Logit showing notable biases for multiple parameters, such as ψ_{10}^* (0.64). RF and SL maintained relatively low biases, while Bayes and Neural exhibited a moderate increase in bias compared to the simple scenario. SVM and RQL had more pronounced bias values.

The complex scenario saw a further increase in bias for most methods, with Logit and RQL showing the largest deviations. Notably, Logit had a bias of 1.86 for ψ_{16}^*, while RQL demonstrated significant biases for parameters like ψ_{20}^* and ψ_{21}^*. Despite the increase in bias across methods, RF, SL, and Bayes maintained relatively lower biases compared to Logit and RQL.

Regarding the standard deviations, RF and SVM exhibited noticeably higher variability in all scenarios. Logit and Bayes also had larger standard deviations, particularly in the medium and complex scenarios. SL and Neural showed more consistent standard deviations across scenarios, with SL demonstrating the most stable performance.

In terms of RMSE, SL performed consistently well across all scenarios, maintaining lower RMSE values comparable to Logit and outperforming the other methods in most cases. RF, Neural, and Bayes had similar RMSE values, slightly higher than SL. RQL had a noticeably higher RMSE, especially in the complex scenario, reflecting its higher biases and greater variability.

Table 6: Results of Study 5 : Simple scenario, $n = 300$

	First time											Second time										
Methods	ψ^*_{10}	ψ^*_{11}	ψ^*_{12}	ψ^*_{13}	ψ^*_{14}	ψ^*_{15}	ψ^*_{16}	ψ^*_{17}	ψ^*_{18}	ψ^*_{19}	ψ^*_{110}	ψ^*_{20}	ψ^*_{21}	ψ^*_{22}	ψ^*_{23}	ψ^*_{24}	ψ^*_{25}	ψ^*_{26}	ψ^*_{27}	ψ^*_{28}	ψ^*_{29}	ψ^*_{210}
	Bias																					
Logit	-0.20	0.19	0.03	0.08	0.03	0.04	0.11	-0.05	-0.05	-0.04	-0.03	-0.19	0.08	-0.04	0.05	-0.04	-0.01	0.05	0.01	0.02	0.02	-0.04
RF	-0.30	0.15	-0.02	-0.01	-0.09	0.11	0.18	-0.02	0.00	-0.02	0.04	-0.42	0.17	0.00	0.02	0.02	0.06	0.07	-0.01	0.03	0.06	-0.08
Bayes	-1.02	0.48	0.13	0.51	-0.10	0.05	0.39	0.01	-0.05	-0.17	-0.03	0.10	0.19	0.04	0.17	-0.11	-0.07	0.09	0.06	-0.09	-0.21	-0.24
Neural	-0.27	0.18	-0.03	-0.01	-0.07	0.09	0.16	-0.01	-0.01	-0.04	0.03	-0.51	0.22	0.00	0.03	0.02	0.05	0.09	-0.01	0.04	0.06	-0.11
SVM	-0.37	0.17	-0.04	-0.04	-0.09	0.07	0.21	0.01	0.01	-0.04	0.05	-0.46	0.24	-0.03	0.02	0.03	0.06	0.09	-0.01	0.04	0.05	-0.12
SL	-0.20	0.14	-0.02	0.03	-0.04	0.13	0.12	-0.01	0.00	-0.05	0.04	-0.41	0.17	0.00	0.03	0.02	0.05	0.07	-0.01	0.04	0.06	-0.08
RQL	-0.67	0.72	-0.02	-0.19	0.43	0.06	0.07	-0.19	-0.08	0.00	0.01	-2.17	0.14	0.02	0.25	0.55	-0.20	0.23	0.03	0.20	0.35	0.00
	Standard deviation																					
Logit	3.35	2.15	1.64	3.76	2.09	2.94	1.55	1.01	1.06	1.97	1.83	6.79	1.54	1.17	1.70	1.72	1.11	1.10	0.94	1.01	1.15	1.60
RF	1.59	1.15	0.88	1.31	0.84	1.40	0.53	0.43	0.57	0.99	0.93	2.31	0.75	0.52	0.70	0.74	0.51	0.35	0.34	0.46	0.55	0.74
Bayes	4.58	4.50	2.16	4.47	1.70	3.14	2.35	0.98	1.32	2.29	2.02	15.23	4.51	2.73	4.08	4.57	3.04	1.99	1.74	3.23	2.92	4.20
Neural	1.80	1.24	1.02	1.52	0.96	1.55	0.61	0.49	0.65	1.13	1.06	2.51	0.79	0.57	0.76	0.79	0.55	0.39	0.37	0.49	0.60	0.80
SVM	1.68	1.22	0.92	1.41	0.86	1.47	0.58	0.44	0.60	1.03	0.97	2.59	0.86	0.60	0.81	0.82	0.56	0.41	0.38	0.51	0.62	0.87
SL	2.13	1.33	1.22	1.74	1.14	1.79	0.68	0.58	0.74	1.34	1.27	2.38	0.77	0.54	0.72	0.76	0.53	0.36	0.35	0.48	0.58	0.76
RQL	4.01	2.46	2.13	3.35	2.67	2.84	1.63	1.34	1.33	2.08	2.23	5.26	2.07	0.90	1.20	2.15	1.50	0.74	0.66	0.76	0.93	1.27
	RMSE																					
Logit	3.36	2.16	1.64	3.76	2.09	2.94	1.56	1.01	1.07	1.97	1.83	6.79	1.54	1.17	1.70	1.72	1.11	1.10	0.94	1.01	1.15	1.60
RF	1.62	1.16	0.88	1.31	0.84	1.41	0.56	0.43	0.57	0.99	0.93	2.35	0.77	0.52	0.70	0.74	0.51	0.35	0.34	0.46	0.56	0.75
Bayes	4.69	4.53	2.16	4.50	1.71	3.14	2.38	0.98	1.32	2.30	2.02	15.23	4.51	2.73	4.08	4.57	3.04	1.99	1.74	3.23	2.92	4.21
Neural	1.82	1.25	1.02	1.52	0.96	1.55	0.63	0.49	0.65	1.13	1.06	2.56	0.82	0.57	0.76	0.79	0.55	0.40	0.37	0.49	0.61	0.81
SVM	1.73	1.23	0.92	1.41	0.87	1.47	0.62	0.44	0.60	1.03	0.97	2.63	0.89	0.60	0.81	0.82	0.57	0.42	0.38	0.52	0.62	0.88
SL	2.14	1.34	1.22	1.74	1.14	1.79	0.69	0.58	0.74	1.34	1.27	2.41	0.79	0.54	0.73	0.76	0.53	0.36	0.35	0.48	0.59	0.76
RQL	4.07	2.56	2.13	3.35	2.71	2.84	1.63	1.36	1.33	2.08	2.23	5.69	2.08	0.90	1.23	2.22	1.51	0.78	0.66	0.78	0.99	1.27
	Ratio																					
RF	0.48	0.54	0.54	0.35	0.40	0.48	0.36	0.42	0.54	0.50	0.51	0.35	0.50	0.45	0.41	0.43	0.46	0.32	0.36	0.46	0.49	0.47
Bayes	1.40	2.10	1.32	1.20	0.82	1.07	1.53	0.97	1.24	1.16	1.10	2.24	2.93	2.33	2.40	2.65	2.74	1.81	1.84	3.20	2.54	2.62
Neural	0.54	0.58	0.62	0.40	0.46	0.53	0.41	0.48	0.61	0.57	0.58	0.38	0.53	0.49	0.45	0.46	0.50	0.36	0.40	0.49	0.53	0.51
SVM	0.51	0.57	0.56	0.38	0.42	0.50	0.40	0.43	0.56	0.52	0.53	0.39	0.58	0.51	0.48	0.47	0.51	0.38	0.40	0.51	0.54	0.55
SL	0.64	0.62	0.74	0.46	0.54	0.61	0.44	0.57	0.70	0.68	0.69	0.36	0.51	0.46	0.43	0.44	0.48	0.33	0.37	0.47	0.51	0.48
RQL	1.21	1.19	1.30	0.89	1.30	0.96	1.05	1.34	1.25	1.05	1.22	0.84	1.35	0.77	0.72	1.29	1.37	0.71	0.70	0.78	0.86	0.79

Table 7: Results of Study 5 : Medium scenario, $n = 300$

	First time											Second time										
Methods	ψ^*_{10}	ψ^*_{11}	ψ^*_{12}	ψ^*_{13}	ψ^*_{14}	ψ^*_{15}	ψ^*_{16}	ψ^*_{17}	ψ^*_{18}	ψ^*_{19}	ψ^*_{110}	ψ^*_{20}	ψ^*_{21}	ψ^*_{22}	ψ^*_{23}	ψ^*_{24}	ψ^*_{25}	ψ^*_{26}	ψ^*_{27}	ψ^*_{28}	ψ^*_{29}	ψ^*_{210}
	Bias																					
Logit	0.64	0.35	0.10	-0.42	-0.02	0.00	0.04	-0.03	0.06	0.01	-0.02	-0.25	0.15	0.07	-0.19	-0.03	-0.14	0.15	-0.01	0.31	-0.18	0.05
RF	0.18	0.48	0.04	-0.60	0.06	-0.07	0.31	-0.07	0.00	0.22	0.00	-1.47	0.15	0.04	-0.27	0.01	-0.10	0.54	-0.08	0.44	-0.19	0.02
Bayes	1.22	0.57	0.05	-0.12	-0.03	-0.12	0.24	-0.03	-0.07	0.14	-0.02	-1.42	0.09	0.02	0.10	0.03	-0.13	0.58	-0.06	0.35	-0.43	0.12
Neural	0.25	0.43	0.05	-0.59	0.03	-0.07	0.21	-0.06	0.04	0.13	0.01	-1.11	0.14	0.01	-0.27	-0.03	-0.13	0.43	-0.06	0.41	-0.16	0.01
SVM	0.22	0.57	0.06	-0.72	0.00	0.00	0.17	0.01	0.01	0.10	0.01	-1.64	0.08	0.02	-0.48	0.01	-0.11	0.52	-0.07	0.54	-0.11	0.09
SL	0.42	0.44	0.06	-0.63	0.02	-0.09	0.19	-0.03	0.03	0.14	0.00	-1.39	0.12	0.02	-0.30	0.01	-0.11	0.51	-0.07	0.44	-0.19	0.05
RQL	-0.36	-0.26	0.01	0.55	-0.06	0.35	0.12	-0.05	-0.02	-0.19	0.02	-0.12	-0.07	0.06	-0.06	-0.52	0.36	0.12	-0.34	-0.37	0.34	-0.06
	Standard deviation																					
Logit	5.43	3.17	2.28	3.49	1.72	3.67	1.07	1.03	1.42	2.82	2.43	4.90	2.60	1.49	2.22	1.74	2.20	0.80	1.08	1.81	1.94	2.64
RF	3.83	1.83	1.88	2.54	1.46	2.82	0.83	0.82	1.14	2.26	1.98	2.52	1.07	0.80	1.19	0.91	1.02	0.45	0.53	0.79	1.00	1.32
Bayes	6.15	3.00	2.27	3.78	1.74	4.05	1.13	1.02	1.40	2.81	2.43	6.77	2.78	2.11	2.99	2.16	3.04	1.03	1.33	2.19	2.87	3.46
Neural	4.07	1.96	1.96	2.68	1.53	3.00	0.88	0.88	1.20	2.39	2.07	2.65	1.15	0.85	1.24	0.97	1.09	0.47	0.57	0.84	1.08	1.40
SVM	4.27	2.04	2.09	2.82	1.61	3.17	0.94	0.90	1.27	2.55	2.22	2.85	1.21	0.90	1.33	1.00	1.10	0.49	0.60	0.87	1.09	1.45
SL	4.20	1.99	2.00	2.72	1.55	3.06	0.91	0.90	1.23	2.47	2.13	2.61	1.10	0.82	1.23	0.94	1.07	0.46	0.55	0.82	1.05	1.37
RQL	5.68	3.33	2.47	7.46	1.81	3.75	1.55	1.22	2.05	3.36	2.95	3.97	1.54	2.44	3.07	2.12	1.58	2.16	0.92	1.73	1.38	3.36
	RMSE																					
Logit	5.47	3.19	2.28	3.52	1.72	3.67	1.07	1.03	1.42	2.82	2.43	4.91	2.61	1.49	2.23	1.74	2.20	0.81	1.08	1.84	1.95	2.64
RF	3.84	1.89	1.88	2.61	1.46	2.82	0.89	0.83	1.14	2.27	1.98	2.92	1.08	0.80	1.22	0.91	1.03	0.70	0.54	0.90	1.02	1.32
Bayes	6.27	3.05	2.27	3.78	1.74	4.05	1.16	1.02	1.40	2.82	2.43	6.92	2.78	2.11	2.99	2.16	3.04	1.19	1.33	2.22	2.90	3.46
Neural	4.08	2.01	1.96	2.74	1.53	3.00	0.91	0.88	1.20	2.39	2.07	2.87	1.16	0.85	1.27	0.97	1.10	0.64	0.57	0.93	1.10	1.40
SVM	4.27	2.12	2.09	2.91	1.61	3.17	0.95	0.90	1.27	2.55	2.22	3.29	1.21	0.90	1.41	1.00	1.10	0.72	0.60	1.02	1.09	1.45
SL	4.22	2.04	2.00	2.79	1.55	3.06	0.93	0.90	1.23	2.48	2.13	2.96	1.11	0.82	1.27	0.94	1.07	0.69	0.56	0.94	1.06	1.37
RQL	5.69	3.34	2.47	7.48	1.81	3.76	1.55	1.23	2.05	3.37	2.95	3.97	1.54	2.44	3.07	2.18	1.62	2.17	0.98	1.77	1.42	3.36
	Ratio																					
RF	0.70	0.59	0.82	0.74	0.85	0.77	0.83	0.80	0.80	0.81	0.82	0.60	0.41	0.54	0.55	0.53	0.47	0.86	0.50	0.49	0.52	0.50
Bayes	1.15	0.96	0.99	1.08	1.01	1.10	1.08	0.99	0.99	1.00	1.00	1.41	1.07	1.42	1.34	1.25	1.38	1.46	1.23	1.21	1.49	1.31
Neural	0.75	0.63	0.86	0.78	0.89	0.82	0.85	0.85	0.85	0.85	0.85	0.59	0.45	0.57	0.57	0.56	0.50	0.78	0.53	0.51	0.56	0.53
SVM	0.78	0.66	0.92	0.83	0.94	0.86	0.89	0.88	0.89	0.91	0.91	0.67	0.46	0.60	0.63	0.58	0.50	0.88	0.56	0.56	0.56	0.55
SL	0.77	0.64	0.88	0.79	0.90	0.83	0.87	0.88	0.87	0.88	0.88	0.60	0.42	0.55	0.57	0.54	0.49	0.84	0.52	0.51	0.55	0.52
RQL	1.04	1.05	1.08	2.13	1.05	1.02	1.46	1.19	1.45	1.20	1.22	0.81	0.59	1.64	1.38	1.25	0.73	2.67	0.90	0.96	0.73	1.27

Table 8: Results of Study 5 : Complex scenario, $n = 300$

	First time											Second time										
Methods	ψ^*_{10}	ψ^*_{11}	ψ^*_{12}	ψ^*_{13}	ψ^*_{14}	ψ^*_{15}	ψ^*_{16}	ψ^*_{17}	ψ^*_{18}	ψ^*_{19}	ψ^*_{110}	ψ^*_{20}	ψ^*_{21}	ψ^*_{22}	ψ^*_{23}	ψ^*_{24}	ψ^*_{25}	ψ^*_{26}	ψ^*_{27}	ψ^*_{28}	ψ^*_{29}	ψ^*_{210}
	Bias																					
Logit	-0.24	0.06	0.11	0.01	0.02	0.14	1.86	-0.01	-0.04	-0.02	-0.14	-0.18	0.15	-0.02	-0.06	-0.20	0.01	0.03	-0.01	-0.17	0.11	0.19
RF	-0.70	0.11	0.04	-0.02	0.04	0.17	1.93	0.01	0.02	0.07	0.06	-0.48	0.18	-0.03	-0.03	-0.18	0.00	0.07	0.04	-0.14	0.13	0.15
Bayes	-0.22	-0.02	0.14	0.32	0.00	0.15	1.88	0.01	-0.11	-0.09	0.03	-0.39	0.49	0.00	-0.41	-0.48	0.06	0.04	0.03	-0.48	-0.05	0.41
Neural	-0.53	0.14	0.08	-0.12	0.05	0.18	1.90	-0.03	-0.01	0.03	-0.04	-0.25	0.15	-0.02	-0.02	-0.17	0.01	0.04	0.00	-0.15	0.11	0.16
SVM	-0.84	0.24	0.06	-0.39	0.10	0.22	1.97	-0.02	0.06	0.11	-0.16	-0.34	0.14	-0.02	-0.05	-0.17	0.00	0.05	0.01	-0.14	0.12	0.17
SL	-0.68	0.11	0.05	-0.03	0.04	0.18	1.92	0.01	0.02	0.06	0.06	-0.42	0.17	-0.02	-0.03	-0.17	0.00	0.06	0.03	-0.14	0.13	0.15
RQL	-0.21	0.10	0.10	-0.20	-0.08	0.13	1.84	-0.01	0.04	0.09	-0.30	0.21	0.01	-0.01	0.01	-0.36	-0.02	0.01	0.07	-0.19	0.16	0.12
	Standard deviation																					
Logit	1.91	1.06	1.10	1.45	0.90	1.68	0.46	0.44	0.66	1.31	1.30	1.53	0.74	0.56	0.69	0.76	0.42	0.23	0.32	0.55	0.53	0.70
RF	1.91	1.05	1.07	1.41	0.91	1.66	0.47	0.44	0.64	1.28	1.24	1.46	0.69	0.53	0.65	0.70	0.40	0.22	0.31	0.50	0.51	0.65
Bayes	2.49	1.38	1.35	1.79	1.10	2.11	0.62	0.54	0.82	1.56	1.59	2.70	1.16	0.76	0.95	1.16	0.55	0.31	0.42	0.91	0.74	1.04
Neural	1.83	1.04	1.05	1.39	0.87	1.63	0.45	0.43	0.63	1.26	1.22	1.55	0.75	0.58	0.71	0.75	0.43	0.23	0.33	0.55	0.56	0.70
SVM	1.80	1.01	0.99	1.34	0.85	1.57	0.44	0.40	0.60	1.21	1.18	1.62	0.77	0.58	0.73	0.77	0.45	0.24	0.33	0.56	0.57	0.75
SL	1.91	1.06	1.07	1.41	0.91	1.66	0.47	0.44	0.64	1.28	1.24	1.48	0.70	0.54	0.66	0.71	0.41	0.22	0.31	0.51	0.51	0.66
RQL	2.36	1.40	1.34	1.80	1.13	2.13	0.56	0.54	0.79	1.61	1.49	1.74	0.85	0.65	0.89	0.86	0.48	0.25	0.37	0.61	0.57	0.99
	RMSE																					
Logit	1.92	1.06	1.10	1.45	0.90	1.68	1.91	0.44	0.66	1.31	1.31	1.54	0.76	0.56	0.69	0.78	0.42	0.23	0.32	0.57	0.54	0.72
RF	2.03	1.06	1.07	1.41	0.91	1.67	1.98	0.44	0.64	1.28	1.24	1.53	0.71	0.53	0.65	0.72	0.40	0.23	0.31	0.52	0.52	0.66
Bayes	2.50	1.38	1.36	1.82	1.10	2.11	1.98	0.54	0.83	1.56	1.59	2.73	1.26	0.76	1.04	1.26	0.55	0.31	0.42	1.02	0.74	1.12
Neural	1.90	1.05	1.05	1.39	0.88	1.64	1.95	0.43	0.63	1.26	1.22	1.57	0.77	0.58	0.71	0.77	0.43	0.23	0.33	0.57	0.57	0.72
SVM	1.99	1.04	1.00	1.40	0.86	1.59	2.02	0.40	0.60	1.21	1.19	1.66	0.78	0.59	0.74	0.79	0.45	0.24	0.33	0.58	0.59	0.77
SL	2.03	1.06	1.07	1.41	0.91	1.67	1.98	0.44	0.64	1.28	1.24	1.54	0.72	0.54	0.66	0.73	0.41	0.23	0.31	0.53	0.53	0.68
RQL	2.37	1.40	1.34	1.81	1.13	2.14	1.93	0.54	0.79	1.62	1.52	1.75	0.85	0.65	0.89	0.93	0.48	0.25	0.37	0.64	0.59	1.00
	Ratio																					
RF	1.06	1.00	0.97	0.98	1.01	0.99	1.04	0.99	0.97	0.98	0.95	0.99	0.94	0.94	0.93	0.92	0.95	0.97	0.96	0.91	0.96	0.92
Bayes	1.30	1.30	1.23	1.25	1.23	1.26	1.03	1.22	1.26	1.19	1.22	1.77	1.66	1.35	1.49	1.60	1.30	1.33	1.31	1.78	1.37	1.55
Neural	0.99	0.99	0.95	0.96	0.98	0.98	1.02	0.96	0.95	0.96	0.94	1.02	1.01	1.03	1.02	0.98	1.01	1.00	1.01	0.99	1.04	0.99
SVM	1.04	0.98	0.90	0.97	0.96	0.94	1.06	0.91	0.91	0.92	0.91	1.08	1.03	1.04	1.06	1.01	1.05	1.05	1.03	1.01	1.08	1.06
SL	1.06	1.01	0.97	0.98	1.02	0.99	1.04	0.99	0.97	0.98	0.95	1.00	0.94	0.96	0.95	0.93	0.96	0.97	0.96	0.92	0.98	0.93
RQL	1.24	1.33	1.22	1.25	1.26	1.27	1.01	1.21	1.20	1.23	1.16	1.14	1.12	1.15	1.28	1.18	1.13	1.08	1.16	1.11	1.09	1.38

Table 9: Results of Study 5 : Simple scenario, $n = 1000$

	First time											Second time										
Methods	ψ^*_{10}	ψ^*_{11}	ψ^*_{12}	ψ^*_{13}	ψ^*_{14}	ψ^*_{15}	ψ^*_{16}	ψ^*_{17}	ψ^*_{18}	ψ^*_{19}	ψ^*_{110}	ψ^*_{20}	ψ^*_{21}	ψ^*_{22}	ψ^*_{23}	ψ^*_{24}	ψ^*_{25}	ψ^*_{26}	ψ^*_{27}	ψ^*_{28}	ψ^*_{29}	ψ^*_{210}
	Bias																					
Logit	-0.10	0.04	0.02	0.06	-0.02	-0.04	0.03	0.00	0.00	0.09	-0.01	-0.03	0.02	0.00	-0.01	0.01	0.01	0.00	0.00	0.00	0.00	0.00
RF	-0.17	0.06	0.02	-0.03	-0.05	0.00	0.11	-0.01	0.02	0.06	0.00	-0.30	0.10	-0.01	-0.01	0.03	0.03	0.05	0.01	0.02	0.02	-0.02
Bayes	0.08	-0.01	0.07	0.06	-0.14	-0.09	0.04	0.01	-0.02	-0.05	-0.09	0.04	0.02	0.00	0.02	0.01	0.00	-0.01	0.01	-0.03	0.00	-0.02
Neural	-0.17	0.13	0.01	0.00	-0.04	-0.02	0.09	0.00	0.00	0.08	0.00	-0.40	0.15	-0.01	0.00	0.05	0.03	0.07	0.01	0.04	0.03	-0.05
SVM	-0.31	0.13	0.01	-0.07	-0.07	-0.01	0.18	0.01	0.02	0.06	0.01	-0.47	0.21	-0.02	0.02	0.05	0.04	0.09	0.02	0.04	0.03	-0.09
SL	-0.11	0.08	0.01	0.04	-0.02	-0.03	0.05	0.00	-0.01	0.09	0.00	-0.23	0.07	-0.01	-0.01	0.04	0.02	0.03	0.01	0.02	0.01	-0.01
RQL	-0.11	0.18	0.01	0.00	0.04	0.00	0.15	-0.01	0.01	-0.03	-0.01	-1.33	0.05	-0.05	0.12	0.27	-0.10	0.26	0.06	0.09	0.16	0.04
	Standard deviation																					
Logit	1.19	0.79	0.69	1.20	0.92	1.07	0.47	0.44	0.45	0.84	0.73	1.69	0.47	0.36	0.45	0.52	0.32	0.28	0.23	0.28	0.37	0.46
RF	0.79	0.55	0.48	0.71	0.48	0.73	0.27	0.24	0.30	0.57	0.50	1.00	0.34	0.25	0.31	0.32	0.22	0.16	0.15	0.19	0.26	0.34
Bayes	1.22	1.39	0.56	1.23	0.59	1.05	0.61	0.31	0.37	0.73	0.62	3.06	1.38	0.75	1.07	1.04	0.72	0.50	0.47	0.80	0.83	0.88
Neural	0.95	0.65	0.58	0.89	0.63	0.90	0.33	0.31	0.37	0.70	0.61	1.17	0.38	0.29	0.36	0.38	0.26	0.19	0.17	0.22	0.30	0.38
SVM	0.78	0.55	0.46	0.68	0.45	0.70	0.26	0.23	0.29	0.56	0.48	1.04	0.37	0.27	0.34	0.32	0.24	0.18	0.17	0.21	0.27	0.36
SL	1.09	0.72	0.66	1.06	0.79	1.01	0.40	0.38	0.43	0.80	0.70	1.17	0.39	0.30	0.36	0.38	0.26	0.19	0.18	0.23	0.31	0.39
RQL	1.61	1.04	0.88	1.60	1.12	1.39	0.62	0.57	0.58	1.09	0.99	2.33	0.64	0.52	0.67	0.69	0.46	0.41	0.31	0.39	0.51	0.62
	RMSE																					
Logit	1.20	0.79	0.69	1.20	0.92	1.07	0.47	0.44	0.45	0.84	0.73	1.69	0.47	0.36	0.45	0.52	0.32	0.28	0.23	0.28	0.37	0.46
RF	0.81	0.55	0.48	0.71	0.48	0.73	0.29	0.24	0.30	0.58	0.50	1.05	0.35	0.25	0.31	0.32	0.22	0.16	0.15	0.19	0.26	0.34
Bayes	1.22	1.39	0.56	1.24	0.61	1.05	0.61	0.31	0.37	0.73	0.63	3.06	1.38	0.75	1.07	1.04	0.72	0.50	0.47	0.80	0.83	0.88
Neural	0.96	0.66	0.58	0.89	0.63	0.90	0.35	0.31	0.37	0.71	0.61	1.23	0.41	0.29	0.36	0.38	0.26	0.20	0.17	0.22	0.31	0.38
SVM	0.84	0.57	0.46	0.68	0.46	0.70	0.32	0.23	0.29	0.56	0.48	1.14	0.42	0.27	0.34	0.33	0.24	0.20	0.17	0.21	0.27	0.37
SL	1.10	0.72	0.66	1.06	0.79	1.01	0.40	0.38	0.43	0.81	0.70	1.19	0.40	0.30	0.36	0.39	0.26	0.19	0.18	0.23	0.31	0.39
RQL	1.62	1.06	0.88	1.60	1.12	1.39	0.64	0.57	0.58	1.09	0.99	2.68	0.64	0.52	0.68	0.75	0.47	0.49	0.32	0.40	0.54	0.63
	Ratio																					
RF	0.68	0.70	0.69	0.59	0.52	0.69	0.62	0.56	0.66	0.69	0.69	0.62	0.75	0.70	0.70	0.61	0.68	0.57	0.67	0.69	0.70	0.74
Bayes	1.02	1.75	0.81	1.03	0.67	0.99	1.29	0.70	0.83	0.87	0.86	1.81	2.91	2.06	2.39	1.98	2.23	1.75	2.05	2.86	2.24	1.93
Neural	0.80	0.83	0.83	0.74	0.69	0.84	0.73	0.70	0.82	0.84	0.83	0.73	0.86	0.81	0.80	0.73	0.79	0.70	0.76	0.79	0.82	0.84
SVM	0.70	0.71	0.66	0.57	0.50	0.66	0.67	0.52	0.63	0.67	0.66	0.67	0.90	0.73	0.76	0.62	0.73	0.69	0.73	0.75	0.74	0.82
SL	0.92	0.91	0.95	0.88	0.86	0.94	0.84	0.86	0.94	0.96	0.95	0.71	0.84	0.84	0.81	0.74	0.81	0.67	0.79	0.81	0.83	0.86
RQL	1.35	1.34	1.27	1.34	1.22	1.30	1.35	1.29	1.28	1.30	1.35	1.59	1.35	1.44	1.52	1.43	1.45	1.71	1.38	1.41	1.46	1.37

Table 10: Results of Study 5 : Medium scenario, $n = 1000$

Methods	First time ψ^*_{10}	ψ^*_{11}	ψ^*_{12}	ψ^*_{13}	ψ^*_{14}	ψ^*_{15}	ψ^*_{16}	ψ^*_{17}	ψ^*_{18}	ψ^*_{19}	ψ^*_{110}	Second time ψ^*_{20}	ψ^*_{21}	ψ^*_{22}	ψ^*_{23}	ψ^*_{24}	ψ^*_{25}	ψ^*_{26}	ψ^*_{27}	ψ^*_{28}	ψ^*_{29}	ψ^*_{210}
Bias																						
Logit	0.37	0.10	-0.03	-0.09	0.00	-0.10	-0.02	-0.02	0.02	0.01	0.06	0.24	0.02	-0.02	-0.03	0.01	-0.08	0.06	0.01	0.07	-0.13	-0.03
RF	0.31	0.43	-0.07	-0.29	0.01	-0.11	0.24	-0.09	-0.02	0.08	0.08	-1.26	0.11	-0.02	-0.14	0.05	-0.08	0.49	-0.07	0.34	-0.15	-0.04
Bayes	1.55	0.12	-0.01	0.44	-0.01	-0.41	0.27	-0.04	-0.16	0.02	-0.02	-0.96	0.17	0.01	0.67	0.05	-0.11	0.59	-0.06	-0.07	-0.59	-0.01
Neural	0.29	0.31	-0.05	-0.26	0.01	-0.12	0.10	-0.05	0.02	0.03	0.07	-0.59	0.11	-0.03	-0.11	0.02	-0.10	0.28	-0.01	0.25	-0.13	-0.08
SVM	0.16	0.56	-0.03	-0.52	-0.01	-0.05	0.13	0.01	0.00	0.01	0.07	-1.60	0.08	-0.02	-0.40	0.02	-0.08	0.51	-0.04	0.48	-0.07	-0.01
SL	0.45	0.33	-0.04	-0.26	0.00	-0.12	0.09	-0.03	0.01	0.02	0.06	-0.68	0.08	-0.03	-0.09	0.05	-0.10	0.31	-0.01	0.26	-0.15	-0.02
RQL	0.33	0.10	0.01	-0.10	0.03	0.12	0.03	-0.04	0.05	-0.07	-0.05	-0.26	-0.02	-0.01	-0.07	-0.08	0.00	0.12	-0.11	0.07	0.07	0.10
Standard deviation																						
Logit	2.29	1.14	1.15	1.61	0.87	1.73	0.49	0.51	0.71	1.38	1.22	1.69	0.64	0.53	0.73	0.53	0.59	0.27	0.31	0.49	0.60	0.77
RF	1.95	0.95	1.03	1.39	0.77	1.52	0.42	0.45	0.61	1.20	1.08	1.24	0.48	0.40	0.54	0.43	0.44	0.20	0.23	0.36	0.46	0.59
Bayes	3.39	1.62	1.09	1.95	0.85	1.99	0.55	0.49	0.68	1.29	1.17	3.87	1.47	1.07	1.63	1.14	1.65	0.60	0.67	1.12	1.36	1.86
Neural	2.14	1.05	1.09	1.52	0.83	1.64	0.47	0.49	0.68	1.30	1.16	1.46	0.56	0.46	0.64	0.48	0.48	0.24	0.27	0.43	0.50	0.67
SVM	2.06	1.02	1.10	1.47	0.82	1.63	0.46	0.47	0.66	1.30	1.15	1.26	0.49	0.38	0.59	0.42	0.43	0.21	0.23	0.36	0.47	0.60
SL	2.11	1.05	1.11	1.52	0.84	1.65	0.46	0.49	0.68	1.32	1.17	1.37	0.55	0.45	0.61	0.46	0.47	0.22	0.26	0.40	0.50	0.66
RQL	2.29	1.15	1.16	1.58	0.90	1.77	0.51	0.52	0.72	1.32	1.22	1.59	0.68	0.52	0.72	0.53	0.60	0.26	0.33	0.49	0.62	0.78
RMSE																						
Logit	2.32	1.15	1.15	1.61	0.87	1.74	0.49	0.52	0.71	1.38	1.22	1.71	0.64	0.53	0.73	0.53	0.59	0.28	0.31	0.50	0.61	0.77
RF	1.97	1.04	1.03	1.42	0.77	1.52	0.49	0.45	0.61	1.20	1.08	1.77	0.50	0.40	0.56	0.43	0.44	0.53	0.24	0.49	0.48	0.59
Bayes	3.73	1.63	1.09	1.99	0.85	2.04	0.61	0.49	0.70	1.29	1.17	3.99	1.48	1.07	1.76	1.14	1.65	0.84	0.67	1.12	1.48	1.86
Neural	2.16	1.10	1.09	1.55	0.83	1.65	0.48	0.49	0.68	1.30	1.17	1.57	0.57	0.46	0.65	0.48	0.49	0.37	0.27	0.50	0.52	0.68
SVM	2.07	1.16	1.11	1.56	0.82	1.63	0.48	0.47	0.66	1.30	1.15	2.04	0.49	0.39	0.71	0.42	0.44	0.55	0.23	0.60	0.48	0.60
SL	2.16	1.10	1.11	1.54	0.84	1.65	0.47	0.49	0.68	1.32	1.17	1.53	0.56	0.45	0.62	0.46	0.48	0.38	0.26	0.47	0.52	0.66
RQL	2.31	1.15	1.16	1.58	0.90	1.77	0.51	0.52	0.72	1.32	1.22	1.61	0.68	0.52	0.72	0.54	0.60	0.29	0.34	0.49	0.63	0.78
Ratio																						
RF	0.85	0.91	0.89	0.88	0.88	0.88	0.99	0.88	0.86	0.87	0.89	1.04	0.78	0.75	0.77	0.81	0.75	1.92	0.78	0.99	0.79	0.77
Bayes	1.61	1.41	0.94	1.24	0.98	1.17	1.23	0.96	0.97	0.93	0.96	2.34	2.32	2.03	2.41	2.14	2.78	3.03	2.16	2.26	2.42	2.42
Neural	0.93	0.96	0.95	0.96	0.95	0.95	0.96	0.96	0.95	0.94	0.95	0.92	0.90	0.86	0.89	0.90	0.83	1.33	0.87	1.00	0.85	0.88
SVM	0.89	1.01	0.96	0.97	0.93	0.94	0.96	0.92	0.93	0.94	0.94	1.20	0.77	0.73	0.98	0.79	0.74	2.00	0.75	1.20	0.78	0.78
SL	0.93	0.95	0.96	0.95	0.96	0.95	0.95	0.95	0.95	0.95	0.96	0.90	0.87	0.85	0.85	0.87	0.82	1.37	0.83	0.95	0.85	0.85
RQL	1.00	1.00	1.01	0.98	1.03	1.02	1.03	1.01	1.01	0.95	1.00	0.94	1.07	0.98	0.99	1.02	1.01	1.03	1.11	0.99	1.03	1.02

Table 11: Results of Study 5 : Complex scenario, $n = 1000$

Methods	First time ψ^*_{10}	ψ^*_{11}	ψ^*_{12}	ψ^*_{13}	ψ^*_{14}	ψ^*_{15}	ψ^*_{16}	ψ^*_{17}	ψ^*_{18}	ψ^*_{19}	ψ^*_{110}	Second time ψ^*_{20}	ψ^*_{21}	ψ^*_{22}	ψ^*_{23}	ψ^*_{24}	ψ^*_{25}	ψ^*_{26}	ψ^*_{27}	ψ^*_{28}	ψ^*_{29}	ψ^*_{210}
Bias																						
Logit	-0.12	0.08	0.04	0.13	0.04	0.07	1.84	-0.02	-0.06	-0.05	-0.13	-0.04	0.15	-0.01	0.01	-0.14	-0.01	0.02	-0.01	-0.15	0.09	0.10
RF	-0.55	0.10	-0.01	0.03	0.04	0.06	1.89	0.01	-0.02	0.07	0.03	-0.31	0.16	-0.02	0.03	-0.10	-0.02	0.06	0.03	-0.11	0.11	0.05
Bayes	0.20	-0.14	0.06	0.57	0.01	0.03	1.81	0.02	-0.18	-0.08	0.03	-0.31	0.62	-0.01	-0.44	-0.48	0.07	0.03	0.02	-0.54	-0.10	0.37
Neural	-0.55	0.12	0.01	0.08	0.06	0.10	1.87	-0.01	-0.05	-0.02	0.21	-0.07	0.16	-0.02	0.02	-0.13	-0.02	0.03	-0.01	-0.15	0.10	0.09
SVM	-0.95	0.29	0.03	-0.50	0.11	0.21	1.98	-0.01	0.10	0.15	-0.19	-0.18	0.14	-0.01	-0.01	-0.10	0.00	0.05	0.01	-0.12	0.10	0.07
SL	-0.53	0.09	-0.01	0.03	0.04	0.06	1.89	0.01	-0.02	0.07	0.03	-0.22	0.16	-0.02	0.03	-0.11	-0.01	0.05	0.02	-0.12	0.10	0.06
RQL	-0.47	0.10	0.02	-0.26	0.06	0.16	1.85	0.01	-0.03	0.22	-0.22	0.20	0.08	-0.02	0.01	-0.29	0.00	0.03	0.04	-0.17	0.17	0.05
Standard deviation																						
Logit	0.93	0.51	0.51	0.69	0.43	0.81	0.24	0.22	0.32	0.64	0.62	0.71	0.35	0.28	0.34	0.33	0.21	0.11	0.15	0.24	0.26	0.35
RF	0.97	0.55	0.54	0.73	0.48	0.87	0.25	0.24	0.34	0.67	0.63	0.68	0.32	0.27	0.32	0.32	0.20	0.10	0.14	0.23	0.25	0.33
Bayes	1.15	0.64	0.58	0.85	0.51	0.99	0.29	0.27	0.39	0.76	0.75	1.37	0.60	0.40	0.51	0.61	0.27	0.15	0.20	0.48	0.37	0.54
Neural	0.92	0.52	0.51	0.70	0.44	0.81	0.24	0.22	0.33	0.64	0.61	0.74	0.36	0.29	0.36	0.35	0.22	0.11	0.15	0.25	0.27	0.36
SVM	0.86	0.48	0.45	0.63	0.41	0.76	0.22	0.19	0.30	0.57	0.55	0.75	0.36	0.29	0.36	0.34	0.22	0.11	0.15	0.24	0.28	0.36
SL	0.97	0.55	0.54	0.74	0.48	0.88	0.25	0.24	0.35	0.68	0.63	0.68	0.33	0.27	0.32	0.32	0.20	0.10	0.14	0.23	0.25	0.33
RQL	1.29	0.73	0.69	0.94	0.61	1.12	0.32	0.30	0.40	0.88	0.78	0.75	0.40	0.30	0.34	0.38	0.23	0.12	0.17	0.27	0.28	0.35
RMSE																						
Logit	0.94	0.52	0.51	0.70	0.43	0.81	1.85	0.22	0.33	0.64	0.63	0.72	0.38	0.28	0.34	0.36	0.21	0.11	0.15	0.28	0.28	0.36
RF	1.11	0.56	0.54	0.73	0.48	0.88	1.91	0.24	0.34	0.68	0.63	0.74	0.36	0.27	0.32	0.33	0.20	0.12	0.15	0.25	0.27	0.33
Bayes	1.17	0.65	0.59	1.02	0.51	0.99	1.83	0.27	0.42	0.76	0.75	1.41	0.86	0.40	0.67	0.78	0.28	0.15	0.20	0.72	0.39	0.65
Neural	1.07	0.53	0.51	0.70	0.45	0.82	1.89	0.22	0.33	0.64	0.64	0.74	0.39	0.29	0.36	0.37	0.22	0.12	0.15	0.29	0.29	0.37
SVM	1.28	0.56	0.45	0.81	0.42	0.79	1.99	0.19	0.31	0.59	0.58	0.77	0.38	0.29	0.36	0.36	0.22	0.12	0.15	0.27	0.29	0.37
SL	1.11	0.56	0.54	0.74	0.48	0.88	1.91	0.24	0.35	0.68	0.64	0.72	0.36	0.27	0.32	0.33	0.20	0.12	0.15	0.26	0.27	0.34
RQL	1.37	0.74	0.69	0.97	0.62	1.13	1.88	0.30	0.41	0.91	0.81	0.77	0.41	0.30	0.34	0.48	0.23	0.12	0.17	0.32	0.32	0.36
Ratio																						
RF	1.18	1.09	1.07	1.05	1.11	1.08	1.03	1.09	1.05	1.06	1.00	1.04	0.96	0.97	0.94	0.92	0.94	1.06	0.98	0.88	0.98	0.92
Bayes	1.24	1.26	1.15	1.45	1.17	1.22	0.99	1.22	1.29	1.20	1.19	1.97	2.29	1.40	1.95	2.16	1.32	1.38	1.35	2.53	1.40	1.81
Neural	1.14	1.03	1.00	1.00	1.03	1.01	1.02	1.00	1.02	1.00	1.02	1.04	1.04	1.03	1.05	1.04	1.05	1.05	1.03	1.02	1.03	1.03
SVM	1.36	1.09	0.89	1.15	0.97	0.97	1.08	0.89	0.95	0.92	0.92	1.07	1.02	1.02	1.05	0.99	1.02	1.07	1.01	0.95	1.06	1.01
SL	1.18	1.09	1.06	1.05	1.11	1.09	1.03	1.10	1.05	1.06	1.01	1.00	0.96	0.97	0.95	0.93	0.96	1.03	0.96	0.90	0.98	0.93
RQL	1.46	1.43	1.36	1.38	1.43	1.40	1.01	1.39	1.24	1.42	1.28	1.08	1.10	1.06	1.00	1.33	1.07	1.10	1.14	1.11	1.16	0.99

Table 12: Monte Carlo standard error of Study 5

Methods	Simple scenario				Medium scenario				Complex scenario			
	Bias		Standard deviation		Bias		Standard deviation		Bias		Standard deviation	
n	300	1000	300	1000	300	1000	300.00	1000	300	1000	300	1000
Logit	0.21	0.05	0.15	0.04	0.17	0.07	0.12	0.05	0.06	0.03	0.04	0.02
RF	0.07	0.03	0.05	0.02	0.12	0.06	0.09	0.04	0.06	0.03	0.04	0.02
Bayes	0.48	0.10	0.34	0.07	0.21	0.12	0.15	0.09	0.09	0.04	0.06	0.03
Neural	0.08	0.04	0.06	0.03	0.13	0.07	0.09	0.05	0.06	0.03	0.04	0.02
SVM	0.08	0.03	0.06	0.02	0.13	0.07	0.10	0.05	0.06	0.03	0.04	0.02
SL	0.08	0.04	0.05	0.03	0.13	0.07	0.09	0.05	0.06	0.03	0.04	0.02
RQL	0.17	0.07	0.12	0.05	0.24	0.07	0.17	0.05	0.07	0.04	0.05	0.03

Appendix 3 – Performance evaluation across scenarios and number of folds for the SuperLearner

We evaluated the influence of the number of folds (no cross-fitting, 2-, 3-, 4-, and 5-fold cross-fitting) on the performance of the SuperLearner in each scenario. The results were similar for $n = 300$ (Table 13) and $n = 1000$ (Table 14).

In the simple scenario, the estimated bias remained low across different numbers of folds, suggesting that the choice of folds did not strongly impact estimation accuracy in this case. In contrast, the medium and complex scenarios exhibited increasing bias as the number of folds increased, particularly for ψ_{10}^* and ψ_{20}^*. For example, in the medium scenario, bias for ψ_{10}^* increased from 0.47 (no cross-fitting) to 0.72 (5 folds), while in the complex scenario, it increased from 0.32 (no cross-fitting) to 0.46 (5 folds).

Standard deviations remained relatively stable in the simple scenario, fluctuating between 0.20 and 0.26 across folds. However, in the medium and complex scenarios, SD increased as the number of folds grew. For instance, in the medium scenario, SD for ψ_{11}^* grew from 0.80 (no cross-fitting) to 1.06 (5 folds), and in the complex scenario, SD for ψ_{11}^* increased from 0.41 (no cross-fitting) to 0.60 (5 folds).

In the simple scenario, RMSE remained consistently low, reinforcing the earlier finding that the number of folds had little impact in low-complexity settings. However, in the medium and complex scenarios, RMSE increased as the number of folds grew, with the most pronounced rise in the medium scenario. For example, in the medium scenario, RMSE for ψ_{20}^* increased from 0.39 (no cross-fitting) to 0.77 (5 folds), and in the complex scenario, RMSE for ψ_{21}^* rose from 0.26 (no cross-fitting) to 0.35 (5 folds).

Table 13: Performance evaluation across scenarios and number of folds for the SuperLearner, $n = 300$

Folds	Simple scenario				Medium scenario				Complex scenario			
	First time		Second time		First time		Second time		First time		Second time	
	ψ^*_{10}	ψ^*_{11}	ψ^*_{20}	ψ^*_{21}	ψ^*_{10}	ψ^*_{11}	ψ^*_{20}	ψ^*_{21}	ψ^*_{10}	ψ^*_{11}	ψ^*_{20}	ψ^*_{21}
	Bias				Bias				Bias			
no cross-fitting	0.00	0.03	-0.02	0.04	0.47	0.04	0.24	0.24	0.32	0.02	0.18	-0.01
2	-0.01	0.05	-0.04	0.06	0.68	0.04	0.45	0.25	0.45	0.03	0.26	-0.01
3	0.00	0.06	-0.03	0.06	0.73	0.00	0.49	0.25	0.45	0.04	0.29	-0.01
4	0.00	0.03	-0.03	0.06	0.71	-0.01	0.54	0.25	0.46	0.07	0.31	-0.01
5	0.01	0.04	-0.03	0.06	0.72	0.05	0.54	0.28	0.46	0.09	0.34	-0.01
	Standard deviation				Standard deviation				Standard deviation			
no cross-fitting	0.20	0.25	0.17	0.15	0.47	0.80	0.31	0.41	0.30	0.41	0.26	0.26
2	0.21	0.25	0.18	0.15	0.45	0.84	0.31	0.35	0.28	0.43	0.27	0.24
3	0.21	0.24	0.18	0.15	0.51	0.91	0.39	0.40	0.31	0.49	0.32	0.27
4	0.21	0.25	0.18	0.17	0.51	0.99	0.48	0.49	0.34	0.53	0.38	0.31
5	0.21	0.26	0.18	0.18	0.57	1.06	0.56	0.53	0.36	0.60	0.43	0.35
	RMSE				RMSE				RMSE			
no cross-fitting	0.20	0.25	0.17	0.15	0.67	0.80	0.39	0.48	0.43	0.42	0.32	0.26
2	0.21	0.25	0.18	0.16	0.82	0.84	0.54	0.43	0.53	0.43	0.37	0.24
3	0.21	0.25	0.18	0.17	0.89	0.91	0.62	0.47	0.55	0.49	0.43	0.27
4	0.21	0.26	0.18	0.18	0.87	0.99	0.72	0.54	0.57	0.53	0.49	0.31
5	0.21	0.26	0.18	0.19	0.91	1.06	0.77	0.60	0.59	0.60	0.55	0.35

Table 14: Performance evaluation across scenarios and number of folds for the SuperLearner, $n =$ 1,000

Folds	Simple scenario				Medium scenario				Complex scenario			
	First time		Second time		First time		Second time		First time		Second time	
	ψ^*_{10}	ψ^*_{11}	ψ^*_{20}	ψ^*_{21}	ψ^*_{10}	ψ^*_{11}	ψ^*_{20}	ψ^*_{21}	ψ^*_{10}	ψ^*_{11}	ψ^*_{20}	ψ^*_{21}
	Bias				Bias				Bias			
no cross-fitting	-0.01	0.01	0.00	0.01	0.15	0.18	0.03	0.29	0.14	0.07	0.12	0.01
2	0.00	0.01	-0.01	0.02	0.27	0.12	0.15	0.26	0.22	0.04	0.15	0.00
3	-0.01	0.02	-0.01	0.02	0.35	0.11	0.19	0.27	0.25	0.04	0.15	0.01
4	0.00	0.02	-0.01	0.02	0.43	0.11	0.24	0.25	0.29	0.03	0.18	0.00
5	0.00	0.01	-0.01	0.03	0.51	0.07	0.29	0.24	0.32	0.02	0.19	-0.02
	Standard deviation				Standard deviation				Standard deviation			
no cross-fitting	0.11	0.13	0.09	0.08	0.26	0.53	0.20	0.42	0.19	0.29	0.16	0.19
2	0.11	0.13	0.09	0.08	0.25	0.47	0.18	0.30	0.16	0.26	0.15	0.16
3	0.11	0.14	0.09	0.09	0.29	0.51	0.23	0.33	0.18	0.30	0.17	0.17
4	0.11	0.14	0.09	0.09	0.32	0.57	0.27	0.34	0.21	0.32	0.22	0.20
5	0.11	0.15	0.10	0.10	0.36	0.62	0.34	0.37	0.23	0.37	0.26	0.22
	RMSE				RMSE				RMSE			
no cross-fitting	0.11	0.14	0.09	0.08	0.30	0.56	0.20	0.51	0.23	0.30	0.20	0.19
2	0.11	0.13	0.09	0.08	0.37	0.49	0.24	0.40	0.28	0.27	0.21	0.16
3	0.11	0.14	0.09	0.09	0.45	0.53	0.30	0.43	0.31	0.30	0.23	0.17
4	0.11	0.14	0.09	0.09	0.53	0.58	0.36	0.42	0.36	0.32	0.28	0.20
5	0.11	0.15	0.10	0.10	0.63	0.62	0.44	0.44	0.40	0.37	0.32	0.22

Appendix 4 – Bayesian information criterion (BIC) for the model distribution choice in the real data analysis

We present the comparison of various survival time models based on the Bayesian Information Criterion (BIC). The models compared include Gaussian, Weibull, Exponential, Lognormal, and Log-logistic distributions. As shown in Table 15, the Log-logistic distribution provided the lowest BIC value for both the Logit and SuperLearner (SL) models, indicating it as the best fitting model for survival time in our analysis.

Table 15: BIC

Model	Logit	SL
Gaussian	3630.61	3540.90
Weibull	3411.74	3321.38
Exponential	3430.40	3336.65
Lognormal	3412.40	3321.27
Loglogistic	3399.61	3308.14
